# The effects of protonation of *meso*-tetraarylporphyrins bearing *N*,*N*-diethylamino groups and the resulting hyperporphyrins on fluorescence and ROS production

**DOI:** 10.1039/d6ra09445g

**Published:** 2026-11-10

**Authors:** Martina Mušković Lukić, Martin Lončarić, Melisa Ciglarić, Ivana Lužaić, Dejana Vujnović, Nela Malatesti

**Affiliations:** a Faculty of Biotechnology and Drug Development, University of Rijeka Radmile Matejčić 2 Rijeka 51000 Croatia nela.malatesti@biotech.uniri.hr; b Laboratory for Photonics and Quantum Optics, Division of Experimental Physics Ruđer Bošković Institute, Bijenička cesta 54 Zagreb 10000 Croatia

## Abstract

Hyperporphyrins formed by protonation of free-base porphyrins with electron-donating groups show strong absorption in the far-red region of the EM spectrum, which could be interesting for applications such as diagnostics and photodynamic therapy (PDT). Red wavelengths penetrate deepest into tissue and are used in PDT to excite photosensitizers, generating singlet oxygen and other reactive oxygen species (ROS) that destroy tumor cells. However, hyperporphyrins and their potential ROS production remain underexplored. Therefore, new porphyrins with varying numbers and arrangements of 4-(*N*,*N*-dimethylamino)phenyl *meso*-substituents, as well as previously known symmetric porphyrin (B_4_), were synthesized to investigate their behavior under conditions of varying acidity. The formation of hyperporphyrins upon titration with methanesulfonic acid (MSA) in dimethyl sulfoxide (DMSO) or a mixture of DMSO and dichloromethane was confirmed by ^1^H NMR and UV/vis spectroscopy. Singlet oxygen (^1^O_2_)/ROS production was evaluated by monitoring the photodegradation of 1,3-diphenylisobenzofuran during 10 minutes of red-light irradiation (675 nm) of the porphyrin without, and with varying amounts of added MSA; the results showed that the stronger the hyperporphyrin effect, the greater the suppression of production of ^1^O_2_ and other ROS. With a very similar trend, protonation of the porphyrins resulted first in a strong decrease in fluorescence, and then in a recovery of fluorescence in accordance with the reduction of the hyperporphyrin effect due to further protonation. The final emission spectra of fully protonated porphyrins showed different shifts compared to the nonprotonated porphyrin, depending on the structure. Two porphyrins with the weakest hyperporphyrin effect (*trans*-A_2_B_2_ and *cis*-A_2_B_2_) showed the highest ^1^O_2_/ROS production-upon complete protonation.

## Abstract 2

Protonation of porphyrins with 4-(*N*,*N*-diethylamino)phenyl meso-substituents leads to a strong decrease in fluorescence and ROS production. Both are restored with complete protonation and the disappearance of the hyperporphyrin effect.

## Graphical abstract

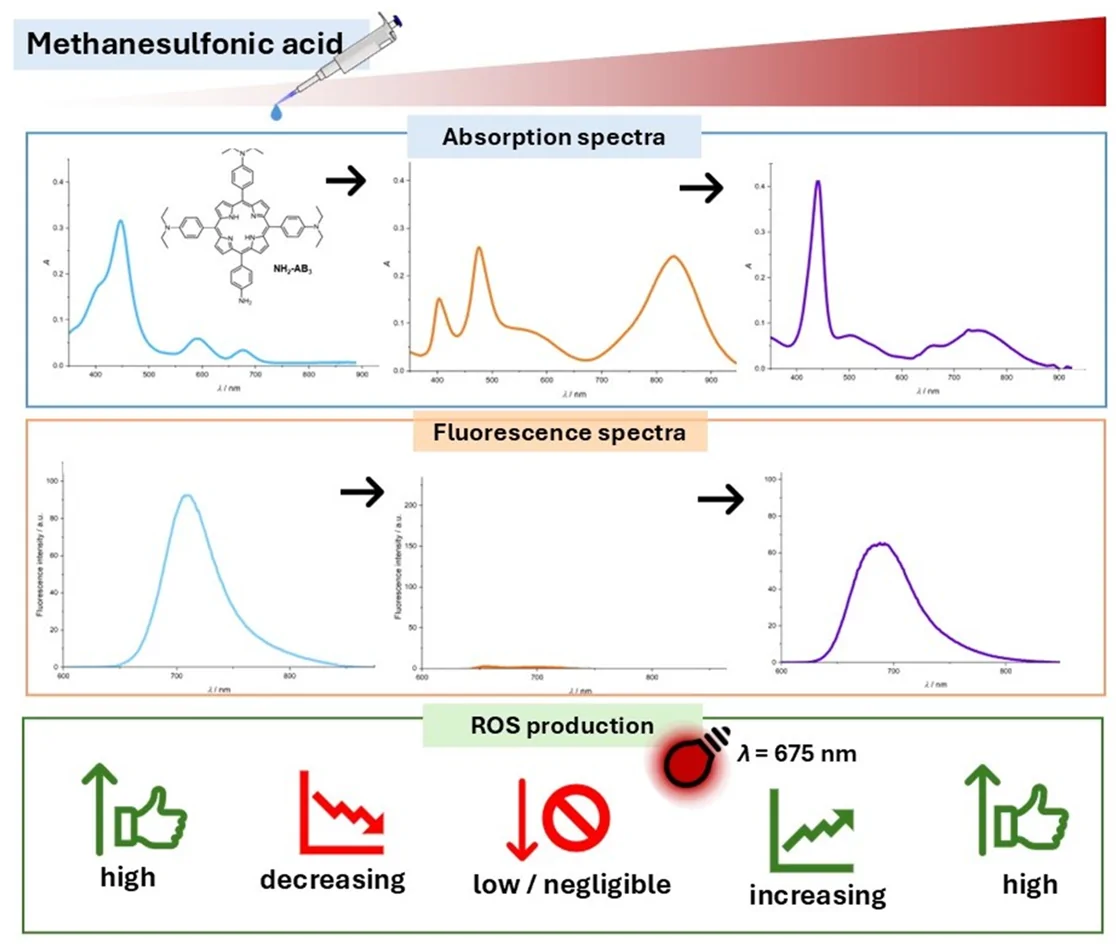

## 1. Introduction

Porphyrins continue to be the focus of many studies and research, thanks to biological importance, their notable optical and electronic properties, available synthetic pathways, and the possibilities for modifying their structure and shape to achieve the adjustment of desired characteristics depending on the intended use.^1–10^ Among many different applications, porphyrins are especially interesting as photosensitizers (PSs) for antitumor and antimicrobial photodynamic therapy (PDT), an approach that involves oxygen and irradiation with visible light on targeted cells and tissues that have accumulated the PS in order to initiate damage through oxidative stress.^11–16^ A photosensitizer excited by light enters into a triplet excited state (^3^PS*), from which, when returning to the ground state, energy can be transferred to molecular oxygen (^3^O_2_), generating highly reactive and cytotoxic singlet oxygen (^1^O_2_). This process in PDT is also known as type II. Returning to the ground state from the ^3^PS* can also occur through a type I process, which involves electron-transfer mediators and the formation of various reactive oxygen species (ROS). Although the type II process is considered the most important for PDT, its strict dependence on oxygen means that the contribution of type I may be favorable under conditions of lower oxygen availability.^17,18^ Besides that, stronger absorption in the red and near-infrared range is considered a desirable property of PSs for use in PDT to treat humans and animals, because those wavelengths penetrate tissue more deeply.^19,20^ Therefore, one of the main limitations of porphyrins in PDT applications is their characteristic absorption spectrum, which exhibits relatively weak absorption in the red region of the electromagnetic spectrum (EMS).^12^

The so-called normal absorption spectra of free-base porphyrins and metalloporphyrins can be described using Gouterman's four-orbital model. According to this model, the Soret band (typically observed in the 400–440 nm region) and Q bands (observed in the 500–650 nm region) arise from electronic transitions involving two nearly degenerate highest occupied molecular orbitals (HOMOs) and two degenerate lowest unoccupied molecular orbitals (LUMOs).^21,22^ Compared to normal porphyrin spectra, hyperporphyrin spectra show bathochromic shifts of the bands, broadening and splitting of the Soret band, and the appearance of additional bands, resulting in significantly higher absorption in the red part of the EMS. This happens because some other orbitals get closer to the porphyrin's four frontier molecular orbitals, so the new transitions require lower energy.^23^ Hyperporphyrin spectra were first described for certain metalloporphyrins, also by Gouterman, with such, either filled p-orbitals and metal-to-ligand, or empty d-orbitals and ligand-to-metal charge transfer transitions.^24^ However, free-base tetraarylporphyrins with aryl orbitals closer to the porphyrin's MOs and the formation of hyperporphyrin through ligand-to-ligand charge transfer have been attracting more and more interest lately, especially *meso*-porphyrins with electron-donating groups such as amino from *p*-aminophenyl or *p*-dimethylamino substituents described by Wamser and his group.^23,25^

Protonation and deprotonation of porphyrins are well studied, and a study using density functional theory (DFT) showed that protonation of the two inner nitrogen atoms leads to distortion from the planar geometry and the adoption of a saddle-like geometry by the porphyrin.^26^ Typically, these inner nitrogens are very weakly basic and require a very strong acid to be protonated. However, in porphyrins with aniline *meso*-substituents, it has been shown that as the number of amino groups increases, the basicity of the inner nitrogens also increases, and it has also been shown that the inner nitrogens are protonated first, followed by protonation of the peripheral groups.^25^ Even before protonation, symmetric *meso*-tetra(*p*-aminophenyl)porphyrin (TAPP) has stronger absorption in the red region of the EMS, with strongly redshifted Soret and Q bands compared with those of tetraphenylporphyrin (TPP). This is due to unusually high energy HOMO interacting with MOs belonging to the aminophenyl group, as shown by time-dependent density-functional theory (TDDFT) calculations. In the same study, TDDFT calculations showed that lower LUMOs (due to mixing with *meso*-aryl character) are mostly responsible for the hyperporphyrin effect and the even stronger Q-band redshifts of the diprotonated form (TAPP diacid).^27^

Due to their much higher absorption in the red part of the EMS, hyperporphyrins appear promising for use in PDT. However, features relevant to potential biomedical applications, such as emission properties and production of singlet oxygen (^1^O_2_) and other types of ROS, have so far been very sparsely described in the literature. A recent article described the synthesis of an iminium-linked hyperporphyrin covalent organic framework, which not only showed the expected shift and higher absorption in the red part of the spectrum, but was also effective against cancer in a mouse model in both normoxia and hypoxia thanks to the high production of superoxide radical anion (O_2_˙^−^) through type I PDT.^28^ Another paper, also published in 2026, confirmed hemin (d-type hyperporphyrin) as a weak ROS producer, but tin protoporphyrin IX (p-type hyperporphyrin) showed significant singlet oxygen production in the study on the effect of metal coordination on protein binding and photosensitization.^29^

When considering hyperporphyrins whose effect is based on the protonation of porphyrins with electron-donating amino groups as PSs for PDT, one needs to keep in mind the pH required for their protonation and the compatibility of such conditions with biological systems. It has been suggested that likely unfavorable ratio could be one of the reasons for their insufficiently explored potential for PDT.^23^ On the other hand, pH levels vary for different organs in the human body, so for example, even though many factors can impact the pH of skin, normal pH range for skin tends to be acidic, 4.1 to 5.8.^30^ Furthermore, tumor microenvironment (TME) is known to be weakly acidic (pH in the range 6.0–6.8),^31^ although intracellular pH, for example for melanoma cells, is around 7.4.^32^

In the previously mentioned study, the authors calculated apparent p*K*_a_ values to indicate relative basicity, among others, for the TAPP diacid, which shows a strong hyperporphyrin effect, and the reported value was 4.57.^25^ The literature value for p*K*_a_ of the conjugate acid for *N*,*N*-diethylaniline is 6.61 at room temperature, so it is a much stronger base than aniline (p*K*_a_ = 4.6) and even *N*,*N*-dimethylaniline (p*K*_a_ = 5.05).^33^ This suggests that replacing aminophenyl groups with *N*,*N*-diethylaminophenyl groups at the *meso*-positions of the porphyrin could further increase the basicity of the inner nitrogen atoms; consequently, a higher proportion of diprotonated species—which exhibit a strong hyperporphyrin effect—could be expected, for instance, in the TME.

All this motivated us to synthesize *meso*-tetra[4-(*N*,*N*-diethylamino)phenyl]porphyrin (B_4_), and previously unreported porphyrins with combinations of 4-(*N*,*N*-diethylamino)phenyl and 4-acetamidophenyl substituents (AB_3_, *trans*-A_2_B_2_ and *cis*-A_2_B_2_), as well as the amino derivative of the AB_3_ porphyrin, to study the impact of protonation and the resulting hyperporphyrins on fluorescence and especially on ^1^O_2_/ROS production, which is crucial to the efficacy of PDT, and to gain better insight into the possible implications for biomedical and some other potential applications.

## 2. Experimental

### 2.1 General

All solvents and reagents used in porphyrin synthesis and spectroscopic analyses were commercially available and used without further purification, unless stated otherwise. The chemicals were purchased from Fisher Chemicals (Germany), VWR Chemicals (USA), and Sigma-Aldrich (Germany). For the spectroscopic analyses, the solvents used were dimethysulfoxide (DMSO) and dichloromethane (DCM) from BDP Prolabo, while titrations were performed with methanesulfonic acid (MSA) purchased from Sigma Aldrich (USA). Reactions were monitored by thin-layer chromatography (TLC) on 0.20 mm silica gel plates 60 Å with fluorescent indicator UV254 from Macherey-Nagel (Germany), while crude products were purified by column chromatography on silica gel 60 Å, 0.04–0.06 mm, also from Macherey-Nagel (Germany), with dichloromethane : methanol (DCM : MeOH) mixtures in varying ratios as eluent.

^1^H and ^13^C NMR analyses were performed at the Laboratory for the NMR spectroscopy, Department of Chemistry, Faculty of Science, University of Zagreb, using Bruker Avance DPX 600 and Bruker Avance Neo 600 spectrometers. ^1^H NMR spectra were recorded at 600 MHz, and ^13^C NMR at 151 MHz. Tetramethylsilane (TMS) was used as an internal standard, and the chemical shifts are expressed in *δ* (parts per million, ppm) relative to TMS (*δ* = 0 ppm), while coupling constants (*J*) are given in Hertz (Hz). All ^1^H and ^13^C NMR Attached Proton Test (APT) spectra of free-base porphyrins (without addition of MSA) were recorded in CDCl_3_, (see Fig. S1–S10 in SI). ^13^C assignments were made based on heteronuclear single quantum coherence (HSQC, 2D ^1^H/^13^C APT) experiment, which was performed on porphyrin AB_3_ in CDCl_3_ (Fig. S4). ^1^H NMR spectra of porphyrin titrations with MSA were recorded either in pure DMSO-*d*_6_, (for porphyrins *cis*- and *trans*-A_2_B_2_) or solvent mixtures of CDCl_3_ and DMSO-*d*_6_ in ratios 1 : 1 (for AB_3_ and NH_2_-AB_3_) and 2 : 1 (B_4_).

Mass spectra of all new porphyrins (Fig. S11–S22) dissolved in MeOH were recorded using a high-resolution mass spectrometer (HRMS) 6546 LC/Q-TOF equipped with a high-performance liquid chromatography 1290 Infinity II HPLC at the Laboratory for bioanalytics at the Ruđer Bošković Institute, Zagreb. Spectra were recorded using positive ionization in resolution mode, and data were collected every second in the mass range of *m*/*z* 100–1000 Da.

### 2.2 Synthesis

Porphyrins B_4_, AB_3_, *trans*-A_2_B_2_ and *cis*-A_2_B_2_ were synthetized according to the Adler–Longo method using two different aldehydes. 4-acetamidobenzaldehyde (0.90 g, 5.5 mmol) and 4-diethylaminobenzaldehyde (2.39 g, 13.5 mmol) were dissolved in propionic acid (70 mL), and freshly distilled pyrrole (1.60 ml, 0.02 mol) was gradually added over 10 minutes. After all the pyrrole had been added, the reaction mixture was heated under reflux for 45 minutes while being exposed to light and air to allow the porphyrinogen to oxidize into porphyrin. Upon completion of the reaction, solvent was removed *in vacuo*, and the reaction mixture was purified by column chromatography with DCM : MeOH mixture (90 : 1) as eluent.

#### 2.2.1 5,10,15,20-tetra[4-(*N*,*N*-diethylamino)phenyl]porphyrin (B_4_)

Porphyrin was isolated as the first fraction and additionally purified with another column chromatography with 130 : 1 DCM : MeOH as eluent. After the precipitation in *n*-hexane, porphyrin was isolated as a dark brown to green solid (150 mg, 3%). ^1^H NMR (CDCl_3_, 600 MHz) *δ*/ppm 8.96 (s, 8H, βH), 8.10 (d, 8H, *J* = 8.4 Hz, Ph(2,6-B)-H), 7.09 (d, *J* = 8.4 Hz, 8H, Ph(3,5-B)-H), 3.66 (q, 16H, *J* = 7.2 Hz, NCH_2_CH_3_), 1.43 (t, 24H, *J* = 7.2 Hz, NCH_2_CH_3_), −2.50 (br s, 2H, P–NH). The obtained spectrum is in agreement with literature data.^34^

#### 2.2.2 5-[4-(*N*-acetamido)phenyl]-10,15,20-tri[4-(*N*,*N*-diethylamino)phenyl]porphyrin (AB_3_)

Isolated as the second fraction in the first column chromatography, porphyrin was additionally purified through another column chromatography, using 130 : 1 DCM : MeOH as an eluent, and isolated as a dark brown to green solid after the precipitation in *n*-hexane (102 mg, 2.1%). ^1^H NMR (CDCl_3_, 600 MHz) *δ*/ppm 9.00–8.81 (m, 8H, βH), 8.19–8.01 (m, 8H, overlapping 2 × Ph(2,6-A)-H + 6 × Ph(2,6-B)-H), 7.84–7.74 (m, 2H, Ph(3,5-A)-H), 7.42 (br s, 1H, CONH), 7.14–6.99 (m, 6H, Ph(3,5-B)-H), 3.79–3.54 (m, 12H, NCH_2_CH_3_), 2.26 (s, 3H, COCH_3_), 1.52–1.30 (m, 18H, NCH_2_CH_3_), −2.53 (br s, 2H, P–NH); ^13^C NMR (CDCl_3_, 151 MHz) *δ*/ppm 168.5, 147.3, 138.7, 137.3, 136.2, 136.1, 135.2, 129.6, 129.4, 121.3, 120.8, 118.2, 117.9, 110.0, 44.6, 24.8, 12.9; HRMS: *m*/*z* calcd for C_58_H_60_N_8_O [M + 3H]^3+^ 295.8369, found 295.8362.

#### 2.2.3 5,15-di[4-(*N*-acetamido)phenyl]-10,20-di[4-(*N*,*N*-diethylamino)phenyl]porphyrin (*trans*-A_2_B_2_)

In the first column chromatography, this porphyrin was isolated as the third fraction. It was then further purified with the second column chromatography applying 130 : 1 DCM : MeOH as an eluent, and isolated after precipitation in *n*-hexane, as a dark brown to green solid (56 mg, 1.2%). ^1^H NMR (CDCl_3_, 600 MHz) *δ*/ppm 9.03–8.95 (m, 8H, βH), 8.22–8.15 (m, 4H, Ph(2,6-A)-H), 8.08–8.03 (m, 4H, Ph(3,5-A)-H), 7.90–7.82 (m, 4H, Ph(2,6-B)-H), 7.50 (s, 2H, CONH), 7.09–7.03 (m, 4H, Ph(3,5-B)-H), 3.71–3.60 (m, 8H, NCH_2_CH_3_), 2.35 (s, 6H, COCH_3_), 1.47–1.37 (m, 12H, NCH_2_CH_3_), −2.62 (br s, 2H, P–NH); ^13^C NMR (CDCl_3_, 151 MHz) *δ*/ppm 168.6, 147.3, 138.6, 137.4, 136.1, 135.2, 129.1, 121.1, 119.0, 117.9, 110.0, 44.6, 24.9, 12.9; HRMS: *m*/*z* calcd for C_56_H_54_N_8_O_2_ [M + 3H]^3+^ 291.1529, found 291.1528.

#### 2.2.4 5,10-di[4-(*N*-acetamido)phenyl]-15,20-di[4-(*N*,*N*-diethylamino)phenyl]porphyrin (*cis*-A_2_B_2_)

Porphyrin was isolated as the fourth fraction in the first column chromatography. It was additionally purified with another column chromatography (130 : 1 DCM : MeOH) and precipitated in *n*-hexane. Porphyrin *cis*-A_2_B_2_ was isolated as a dark brown to green solid (34 mg, 0.7%). ^1^H NMR (CDCl_3_, 600 MHz) *δ*/ppm 8.98–8.73 (m, 8H, βH), 8.06–8.03 (m, 8H, overlapping 4 × Ph(2,6-A)-H + 4 × Ph(3,5-A)-H), 7.78–7.65 (m, 4H, Ph(2,6-B)-H), 7.43 (s, 2H, CONH), 7.08–6.97 (m, 4H, Ph(3,5-B)-H), 3.68–3.53 (m, 8H, NCH_2_CH_3_), 2.27 (s, 6H, COCH_3_), 1.44–1.30 (m, 12H, NCH_2_CH_3_), −2.62 (br s, 2H, P–NH); ^13^C NMR (CDCl_3_, 151 MHz) *δ*/ppm 168.5, 147.3, 138.4, 137.4, 136.2, 135.1, 129.3, 121.6, 118.5, 117.9, 110.0, 44.6, 24.8, 12.9; HRMS: *m*/*z* calcd for C_56_H_54_N_8_O_2_ [M + 3H]^3+^ 291.1529, found 291.1529.

#### 2.2.5 Synthesis of the 5-(4-aminophenyl)-10,15,20-tri[4-(*N*,*N*-diethylamino)phenyl]porphyrin (NH_2_-AB_3_)

Porphyrin AB_3_ (101 mg, 0.12 mmol) was dissolved in 18% aqueous HCl solution (60 mL) and heated under the reflux for two hours under constant stirring, protected from light. Upon completed hydrolysis, confirmed by TLC chromatography, the solvent was removed *in vacuo*. The resulting solid was neutralized by dissolving in a 9 : 1 DCM : triethylamine (TEA) solution and stirred for 30 minutes at room temperature. The reaction mixture was then washed with distilled water (3 × 100 mL) and brine (1 × 100 mL). The organic layer was dried over anhydrous sodium sulfate (Na_2_SO_4_), which was then removed by filtration, followed by the solvent removal *in vacuo*. The porphyrin product was further purified by column chromatography on silica gel using DCM : MeOH : TEA (130 : 1 : 0.1) followed by the precipitation in *n*-hexane. The pure product, NH_2_-AB_3_, was isolated as a dark green-brown solid (86 mg, 85%). ^1^H NMR (CDCl_3_, 600 MHz) *δ*/ppm 9.02–8.87 (m, 8H, βH), 8.13–8.08 (m, 6H, Ph(2,6-B)-H), 8.06–8.01 (m, 2H, Ph(2,6-Am)-H), 7.12–7.04 (m, 8H, overlapping 6 × Ph(3,5-B)-H + 2 × Ph(3,5-Am)-H), 3.99 (br s, 2H, NH_2_), 3.66 (q, 12H, *J* = 7.0 Hz, NCH_2_CH_3_), 1.43 (t, 18H, *J* = 7.0 Hz, NCH_2_CH_3_), −2.53 (br s, 2H, P–NH); ^13^C NMR (CDCl_3_, 151 MHz) *δ*/ppm 147.3, 145.8, 136.2, 136.1, 135.7, 133.0, 129.7, 129.6, 120.8, 120.6, 119.6, 113.4, 110.0, 44.6, 12.9; HRMS: *m*/*z* calcd for C_56_H_58_N_8_ [M + H]^+^ 843.4857, found 843.4833.

### 2.3 Spectroscopic analyses

UV-vis spectra were measured on an Agilent Cary 60 UV-vis spectrophotometer (USA) in quartz cuvette (Hellma Analytics, Germany) over a wavelength range of 350 to 950 nm. Porphyrins were prepared in pure DMSO (*cis*-A_2_B_2_ and *trans*-A_2_B_2_) or in DCM : DMSO solvent mixture in 2 : 1 ratio (B_4_) or 1 : 1 ratio (AB_3_ and NH_2_-AB_3_). For titrations, porphyrins were tested in concentration of ∼6 µM and titrated with MSA that was diluted in H_2_O or concentrated and added gradually in amounts ranging from 5 nmol to 3 mmol. All spectra are reported as molar absorption coefficient (*ε*) over a wavelength (*λ*) (Fig. S29–S33).

Fluorescence measurements were performed in quartz cuvettes (Hellma Analytics, Germany) on an Agilent Cary Eclipse spectrophotometer (USA). Spectra were collected in the emission range of 500 to 950 nm with the excitation wavelength set at 440 or 445 nm, and with both excitation and emission slits set to 5 nm. All stock solutions (6 mM) were prepared either in pure DMSO (*cis*-A_2_B_2_ and *trans*-A_2_B_2_) or in 1 : 1 DCM : DMSO solvent mixture (B_4_, AB_3_ and NH_2_-AB_3_) and further diluted in DMSO to final concentration. The acid used for spectroscopic titrations, MSA, either in concentrated form or diluted in H_2_O, was added to the porphyrin solution in amounts ranging from 20 nmol to a maximum of 6 mmol.

### 2.4 Singlet oxygen/ROS production

Singlet oxygen (^1^O_2_) and reactive oxygen species (ROS) production was determined by measuring the decrease of fluorescence resulting from the photodegradation of 1,3-diphenylisobenzofuran (DPBF). Porphyrins were prepared as 6 mM stock solutions in DMSO or solvent mixtures. For B_4_, a 2 : 1 DCM : DMSO mixture was used; for AB_3_ and NH_2_-AB_3_, a 1 : 1 DCM : DMSO mixture was used, whereas *cis*-A_2_B_2_ and *trans*-A_2_B_2_ stock solutions were prepared in DMSO. All stock solutions were further diluted with DMSO to a final porphyrin concentration of 2 µM. A DPBF solution was prepared in DMSO at a final concentration of 10 µM. In addition to the studied porphyrins, ^1^O_2_/ROS production was evaluated for 5,10,15,20-(3-pyridyl)porphyrin(Py-B_4_) under the same conditions as tested porphyrins and the control.

The photodegradation of DPBF was measured, after mixing with porphyrins in 1 : 1 ratio, in the presence of each porphyrin alone and after the addition of 30 µmol, 300 µmol, or 3 mmol of MSA, added only as concentrated acid. Controls included measurements of the photodegradation of DPBF in DMSO alone and in the presence of MSA at the same concentrations used in the porphyrin-containing samples.

The photodegradation of DPBF was monitored by measuring the loss of fluorescence intensity at *λ*_emm_ = 460 nm, with *λ*_ex_ = 410 nm starting with the time zero and after every 30 or 60 s of irradiation with red light (*λ* = 675 nm, 41 mW cm^−2^) for a total irradiation time of 10 min, corresponding to a total light dose of 24.6 J cm^−2^. All measurements were performed in duplicate.

The results were presented in two ways: first, as the time-integrated DPBF photodegradation, calculated as the area under the curve (AUC), following the calculations previously reported.^35^ The second way presents the calculated kinetic rate constant (*k*) of DPBF photodegradation with the correction of the absorbance of the porphyrin at the irradiation wavelength by applying the formula adapted and modified according to the:^36^
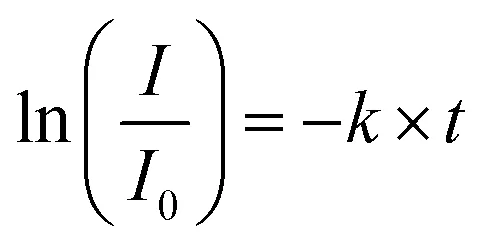

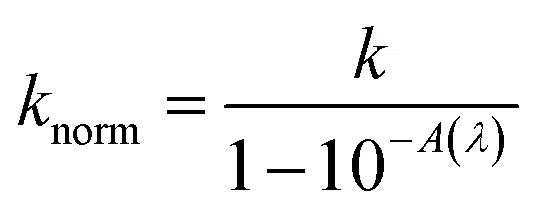
where (*I*/*I*_0_) is the ratio of the fluorescence at the measured time point with the fluorescence at the timepoint zero, *k* - kinetic rate constant of DPBF photodegradation, *t* is time, *k*_norm_ is absorption normalized constant and *A*(*λ*) is absorption of porphyrin at the irradiation source wavelength.

### 2.5 Light source for singlet oxygen/ROS production measurements

The light source used in this study to measure the production of singlet oxygen and reactive oxygen species (ROS) was specifically designed and constructed at a wavelength of 675 nm to match the maxima of the last, *Q*_*x*_(0–0), band in the absorption spectra of the prepared porphyrins. For this purpose, a high-brightness LED with a central emission wavelength of 675 nm was selected. The LED is powered by a constant-current driver, and the thermal management system incorporates a heat spreader and a heat sink to ensure stable wavelength and optical power characteristics during sample irradiation. The source is designed for use with an irradiation chamber that is shielded from the ambient light and equipped with a cuvette holder. The use of an optical dome and light-concentrating optics ensures uniform illumination of the aperture covering the sample-filled section of the cuvette. Spectral and radiometric characterization was performed using a fiber-optic spectrometer and a radiometrically calibrated filtered photodiode, yielding the following results: *λ* = 675 nm, Δ*λ*_FWHM_ = 22 nm, and a fluence rate of 41 mW cm^−2^. The source spectrum and a photograph of the setup are shown in Fig. S35 and S36. The light source was designed and characterized at the Laboratory for Photonics and Quantum Optics and fabricated at the workshop of the Division of Physical Chemistry at the Ruđer Bošković Institute.

To determine the number of the absorbed photons for each porphyrin after illumination during the DPBF photodegradation experiment (Table S3), a formula that connects the optical properties of a porphyrin (*A*(*λ*)-absorption at the irradiation wavelength) with the wavelength (*λ*), power (*P*), and a time period of irradiation (Δ*t*) was used, following the calculations previously reported.^37^

## 3. Results and discussion

### 3.1 Synthesis and characterization of new porphyrins

New *meso*-tetraarylporphyrins with combinations of 4-(*N*-acetamido)phenyl or 4-aminophenyl (= group A) with 4-(*N*,*N*-diethylamino)phenyl (= group B) as *meso* substituents were prepared by a modified Adler–Longo method using 4-acetamidobenzaldehyde, 4-diethylaminobenzaldehyde and pyrrole in boiling propionic acid, followed by acid hydrolysis of the acetamido-group according to the procedure used for the synthesis of analogous (pyrid-3-yl)porphyrins in our previous research.^38^ Unlike pyridylporphyrins, where the two porphyrin isomers from Adler–Longo reaction mixture could not be separated, in our new series of porphyrins, after multiple purifications by column chromatography, pure *trans*-A_2_B_2_ and *cis*-A_2_B_2_ were successfully obtained, as well as B_4_ and AB_3_ that was used in hydrolysis to produce NH_2_-AB_3_ (Scheme 1). Previously reported^34^ porphyrin B_4_ was confirmed by ^1^H NMR, while the structures of new porphyrins were analyzed and confirmed by ^1^H and ^13^C NMR, and mass spectra (see Fig. S1–S22 in SI). In the mass spectra of all the porphyrins, peaks for all three [M + H]^+^, [M + 2H]^2+^ and [M + 3H]^3+^ ions can be seen, and interestingly, below each main peak there are two peaks corresponding to the loss of two protons. Such signals were not observed in the mass spectra of our pyridylporphyrins, so they might be specific to hyperporphyrins; however, we have not been able to find similar examples in the literature.

**Scheme 1:**
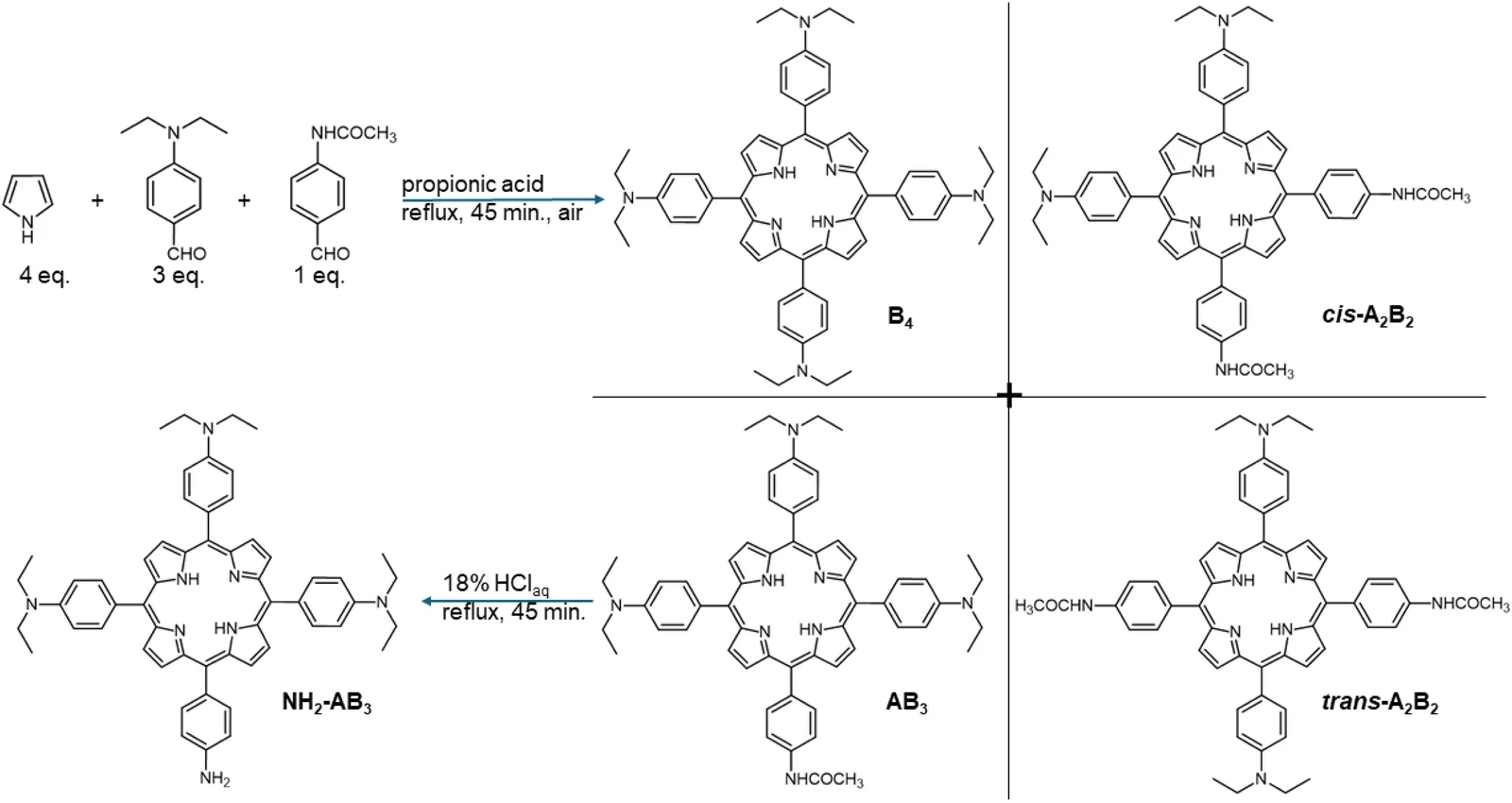
Synthetic routes to porphyrins B_4_, AB_3_, *trans*-A_2_B_2_, *cis*-A_2_B_2_ and NH_2_-AB_3_ and their structures.

### 3.2. ^1^H NMR spectroscopic study of the porphyrins' protonated forms

Protonated forms of the prepared porphyrins were studied by ^1^H NMR through acid titration with methanesulfonic acid (MSA). Two porphyrin isomers, *cis*- and *trans*-A_2_B_2_ were dissolved in DMSO-*d*_6_, while the other porphyrins needed to be dissolved in CDCl_3_ : DMSO-*d*_6_ mixtures (1 : 1 for AB_3_ and NH_2_-AB_3_, and 2 : 1 for B_4_) due to insufficient solubility in pure DMSO. Symmetric B_4_ is insoluble in many solvents, similar to previously reported *meso*-tetra[4-(*N*,*N*-dimethylamino)phenyl]porphyrin,^39^ and due to low solubility in DMSO-*d*_6_, a lower initial concentration was prepared for B_4_ (2 mM), as opposed to 6 mM for all the other porphyrins, in the same starting volume of the sample (500 µL). The first addition of MSA to B_4_ was 2 µmol, while 5 µmol for all other porphyrins, and the third, fourth and fifth (the last) additions of MSA were the same for all five porphyrins −10, 150 and 750 µmol respectively. However, B_4_ could not be completely dissolved at the early protonation stages, which may explain the very small changes in the spectra after the first addition of acid, and the poorly resolved peaks and some additional peaks in the spectra, especially in the aliphatic region (Fig. S23b).

In the assignments of ^1^H NMR spectra, for the internal (*i.e.* those attached to the pyrrolic nitrogens), and different aromatic protons, abbreviations shown on the representative structures, were used (Fig. 1).

**Fig. 1:**
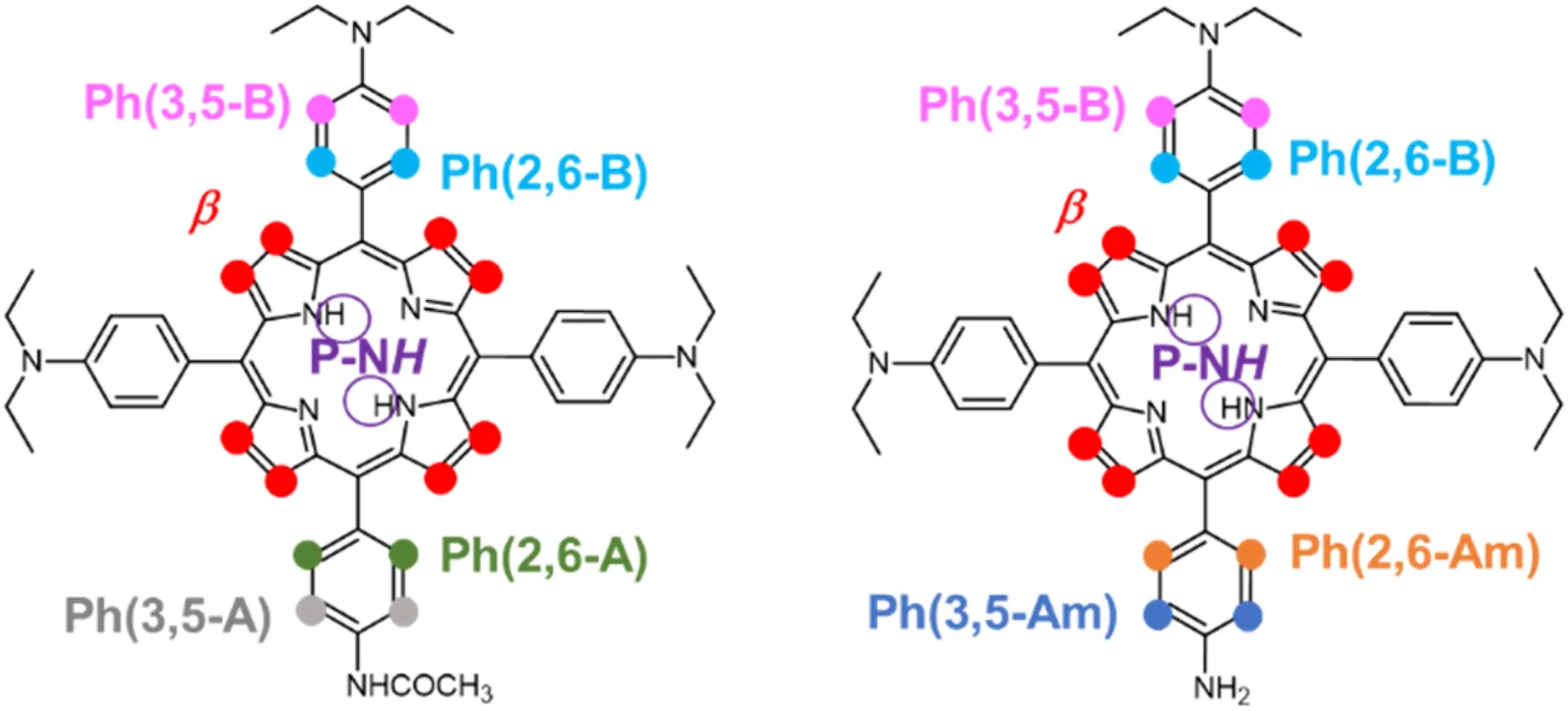
Structures of representative porphyrins AB_3_ (left) and NH_2_-AB_3_ (right) with notations used for hydrogens in ^1^H NMR spectra.

As in the previously reported NMR study of hyperporphyrins with combination of pyrid-4-yl and 4-aminophenyl as *meso-*substituents,^40^ also in our case there are two clearly distinguishable stages of porphyrin protonation. In the stacked spectra (shown in Fig. 2 and S23–S27), 2nd and 3rd row spectra can be assigned to diprotonation of the porphyrins and strong hyperporphyrin effect in the first stage (leading to B_4_H_2_^2+^, AB_3_H_2_^2+^, -A_2_B_2_H_2_^2+^, *cis*-A_2_B_2_H_2_^2+^ and NH_2_-AB_3_H_2_^2+^), while the 4th and 5th row spectra point to the second stage, and they present shifts characterizing two or one remaining electron-donating (amino) group available for charge transfer, and complete protonation of the porphyrins (resulting in formation of B_4_H_6_^6+^, AB_3_H_5_^5+^, trans- and *cis*-A_2_B_2_H_4_^4+^ and NH_2_-AB_3_H_6_^6+^ respectively), where hyperporphyrin effect is no longer present.

**Fig. 2:**
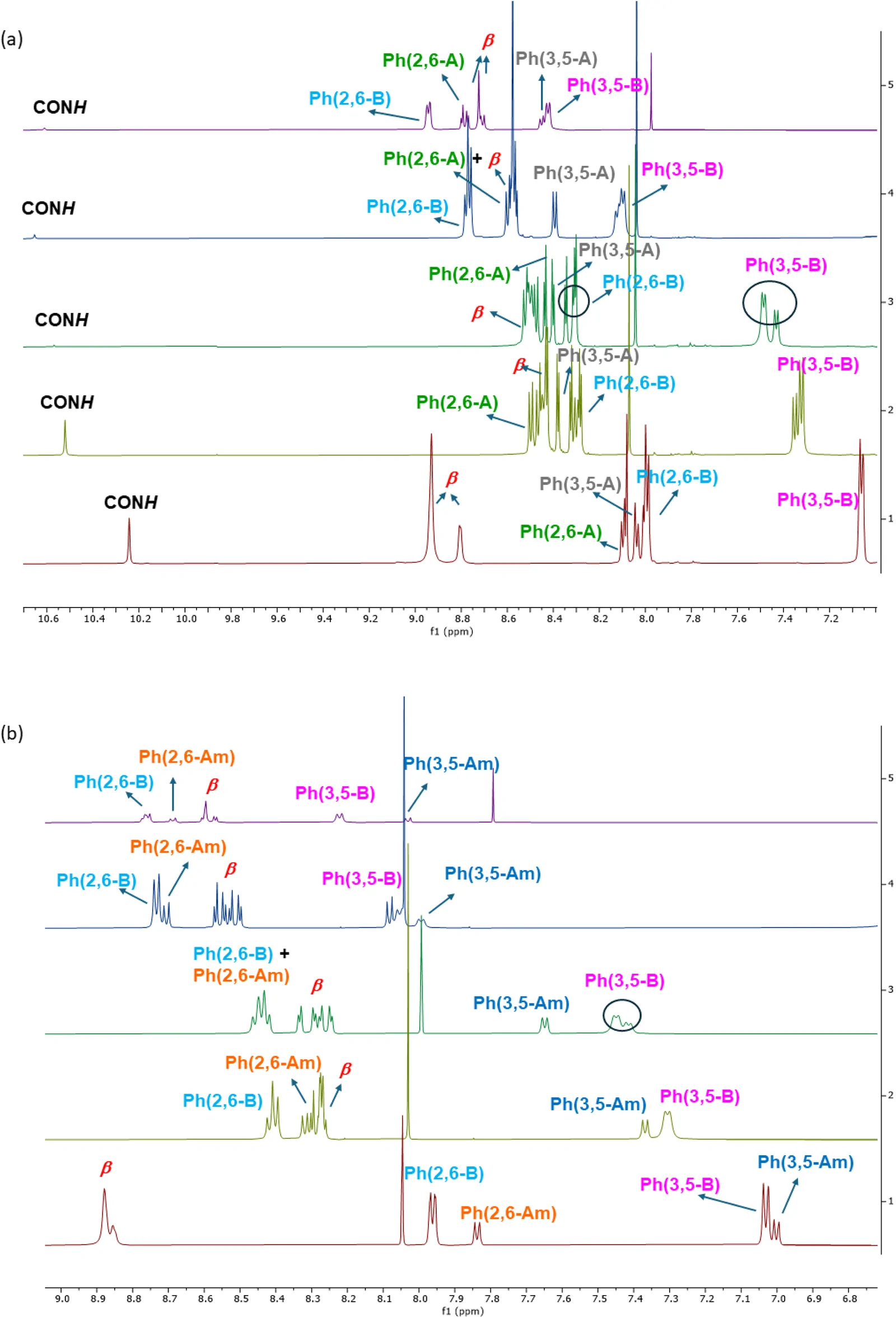
^1^H NMR spectra in aromatic region for (a) AB_3_ and (b) NH_2_-AB_3_ porphyrins (6 mM) recorded in CDCl_3_ : DMSO-*d*_6_ (1 : 1) after titrations with MSA at room temperature. Starting volume of the porphyrin solution was 500 µL, and the volumes of MSA consecutively added were: 0 µL (1st row/bottom spectra), 5 µL (2nd row), 10 µL (3rd), 150 µL (4th) and 750 µL (5th).

Previous spectroscopic studies of porphyrins with one or more 4-aminophenyl or 4-(*N*,*N*-diethylamino)phenyl substituents have found that the first two protonations occur on the inner (pyrrole) nitrogens and that there is no evidence of a monoprotonated intermediate.^25,39–41^ In those ^1^H NMR studies, for some of the porphyrins, a downfield shift of approximately ∼3.5 ppm could be detected for the inner pyrrole protons (P–NH) during and after deprotonation.^39,40^ In our study, the P–NH signal present in the proton NMR spectra for all porphyrins between −2.50 and −2.60 ppm before acid titrations, disappears already after the first addition of MSA. New P–NH signals could not be detected in spectra; only after the last MSA addition and complete protonation of the porphyrins P–NH signals could be observed, although as very small peaks between −0.25 and −0.70 ppm (Fig. S28), similar to those reported by Wang and Wamser.^40^

All the aryl protons show a continuous downfield shift throughout titration stages, but as expected, the most pronounced shifts are those for Ph(3,5-B), especially in the second stage of the protonation when the peripheral groups are also protonated and therefore no longer electron-donating. The overall shift, going from unprotonated to fully protonated porphyrin, for Ph(3,5-B) protons is ∼1.3 ppm for porphyrins AB_3_ (Fig. 2a), and *trans*- and *cis*-A_2_B_2_ (Fig. S25a and S26a), and somewhat less (∼1.2 ppm) for B_4_ (Fig. S23a) and NH_2_-AB_3_ (Fig. 2b). Overall shifts for Ph(2,6-B) protons are ∼0.9 ppm for AB_3_, *trans*- and *cis*-A_2_B_2_, and again slightly less for B_4_ and NH_2_-AB_3_ (∼0.75 ppm). Shift for the Ph(2,6-Am) protons was 0.85 ppm, thus, between those two values, but for the Ph(3,5-Am) it was 1.05 ppm (Fig. 2b), which is lower than for all other porphyrins' Ph(3,5-B) protons, probably due to the free amino group being weaker electron-donating compared to the tertiary amine (−NEt_2_). Overall shifts for the aryl protons of the substituent with the acetamido-group were significantly lower, ∼0.65 ppm for Ph(2,6-A) and ∼0.4 ppm for Ph(3,5-A) protons, and the signal for the amide proton (CONH) gradually decreased and shifted downfield (Fig. 2a, S25a and S26a).

Interestingly, for acetamido AB_3_ and NH_2_-AB_3_, at the hyperporphyrin stage, Ph(3,5-B) and Ph(2,6-B) protons (only visible for AB_3_) appear as two signals (circled in Fig. 2), possibly due to their structure with two of the B-rings being *cis*, and one *trans* to the A-ring. A similar pattern was previously reported for A_3_PyP.^40^

The pyrrole *β* protons for all the porphyrins exhibited an upfield shift in the first stage, and then a downfield shift in the second stage of the protonations, resulting with a small upfield shift for the fully protonated porphyrin compared with the signal before the acid addition, which is in line with previously reported data indicating a diminished ring current effect during hyperporphyrin formation.^40^

In the aliphatic region, signals for the protons of the *N*-ethyl groups for all the porphyrins shift downfield, with more pronounced shifts for the protons closer to nitrogen (NC*H*_2_CH_3_) (Fig. S23–S27). As previously observed for *N*-methyl groups,^39^ separate signals for ethyl protons without and with nitrogen protonation as well as N^+^H protons could not be observed due to the fast exchange. Furthermore, the COCH_3_ signal shows a small downfield shift, and, like aryl Ph(3,5-B) and Ph(2,6-B) protons, appears as two signals at the hyperporphyrin stage for AB_3_ (Fig. S24), *trans*-A_2_B_2_ (Fig. S25) and *cis*-A_2_B_2_ (Fig. S26). Finally, the signal of the free amino group for the NH_2_-AB_3_ disappears already after the first addition of MSA (Fig. S27), presumably also due to the fast exchange with the acid.

### 3.3 Changes in UV/vis absorption and fluorescence spectra of the porphyrins during acid titration

For comparing the spectroscopic data collected from the analysis of porphyrins B_4_, AB_3_, *trans*-A_2_B_2_, *cis*-A_2_B_2_ and NH_2_-AB_3_, it is important to note the possible types of hyperporphyrins and the number of resonance forms for each (Scheme S1 and Table S1). Wang and Wamser described and explained this in great detail in their study of porphyrins with combinations of 4-aminophenyl *meso*-substituent, as an example of an electron-donating group, and pyrid-4-yl as an electron-withdrawing group.^25^ They found that the pyridyl nitrogens are protonated first, then pyrrole, and finally the amino-group nitrogens, or the pyridyl groups at the same time as the inner (pyrrole) nitrogens when there are two or more anilines at the porphyrin's *meso*-positions. In our case, electron-donating 4-(*N*,*N*-diethylamino)phenyl group is in combinations with 4-(*N*-acetamido)phenyl, which cannot contribute to hyperporphyrin through electron-donation due to the inductive electron-withdrawing effect of the carbonyl group, or with one 4-aminophenyl, which is also electron-donating and hyperporphyrin contributor, albeit slightly weaker than the tertiary amine. Therefore, B_4_ and NH_2_-AB_3_ can have four resonance forms for each hyperporphyrin Type B and Type C when diprotonated on the inner nitrogens (B_4_H_2_^2+^ and NH_2_-AB_3_H_2_^2+^), and they can also show secondary hyperporphyrin spectrum when they are tetraprotonated (B_4_H_4_^4+^ and NH_2_-AB_3_H_4_^4+^), that is when they have two inner nitrogens protonated as well as two peripheral diethylamino groups, but two aminophenyl groups remain available for charge-transfer interactions. When all six nitrogens are protonated, *i.e.*B_4_H_6_^6+^ and NH_2_-AB_3_H_6_^6+^ are formed, their spectra return to normal. Porphyrin AB_3_ can also have primary (for AB_3_H_2_^2+^) and secondary hyperporphyrin spectra (for AB_3_H_4_^4+^), with both Type B and Type C hyperporphyrin, though primary with fewer resonant forms than B_4_H_4_^4+^ and NH_2_-AB_3_H_4_^4+^, and with five protonations its spectrum returns to normal. Isomers *trans*-A_2_B_2_ and *cis*-A_2_B_2_ when diprotonated form hyperporphyrin, however, *cis*-A_2_B_2_H_2_^2+^ can form both Type B and Type C hyperporphyrin, while *trans*-A_2_B_2_H_2_^2+^ only Type B, thus is expected to show the weakest hyperporphyrin effect at this stage. The triprotonated form of both isomers still has one electron-donating group available for charge transfer, so a small hyperporphyrin effect could be anticipated for Type B, which has only one resonant form, and both isomers return to normal spectra after reaching four protonations (Scheme S1 and Table S1).

The changes in the absorption spectra of all the porphyrins upon titration with MSA are shown in Fig. S29–S32. For B_4_, protonation with MSA results in the formation of B_4_H_2_^2+^ due to the protonation of the inner pyrrole nitrogens, as evidenced by the splitting of the Soret band from 447 nm into two peaks at 403 and 478 nm. Moreover, a characteristic strong peak at 850 nm is observed, indicating the formation of a hyperporphyrin specie. Upon the addition of MSA in the µmol range, presumably B_4_H_4_^4+^ is mostly formed, which is evidenced by the hypsochromic shift of the peak at 850 nm to 765 nm and the merging of the split Soret bands into a single peak at 433 nm. The fully protonated porphyrin spectrum is characterized by an intense Soret band at 441 nm and the formation of a band at 654 nm.

Similar changes were observed for the porphyrin NH_2_-AB_3_. Upon formation of NH_2_-AB_3_H_2_^2+^ by diprotonation with MSA, the Soret band splits into peaks at 400 and 473 nm, and a strong peak at 843 nm. Upon further protonation and formation of NH_2_-AB_3_H_4_^4+^, the strong peak at 843 nm undergoes a hypsochromic and hypochromic shift to 780 nm, while the Soret band recovers as a single band. Complete protonation of NH_2_-AB_3_ is indicated by the formation of a peak at 652 nm and a strong Soret band at 442 nm.

It is worth mentioning that high molar absorption coefficient calculated for diprotonated NH_2_-AB_3_ at 843 nm (99 900 M^−1^ cm^−1^) drops almost by half in its secondary hyperporphyrin spectrum (NH_2_-AB_3_H_4_^4+^) at 781 nm (55 000 M^−1^ cm^−1^), and similar happens for B_4_ (from 62 500 M^−1^ cm^−1^ at 850 nm to 33 100 M^−1^ cm^−1^ at 768 nm) and AB_3_ (from 117 000 M^−1^ cm^−1^ at 828 nm to 63 100 M^−1^ cm^−1^ at 772 nm) (Table 1). Unexpectedly, B_4_ showed the lowest absorption values compared to the other two porphyrins, even though it has the highest number of electron-donating groups in its structure. However, the aforementioned solubility issue with this porphyrin and the fact that more DCM had to be used in the mixture with DMSO to prepare the solution for the measurements, resulting in a different solvent composition from that used for the other two porphyrins, must be taken into account.

**Table 1:** Summarized absorption (*λ* and *ε*) and fluorescence (*λ*) properties of porphyrins B_4_, AB_3_, *trans*-A_2_B_2_, *cis*-A_2_B_2_ and NH_2_-AB_3_ in DMSO or DCM : DMSO mixtures at room temperature and upon addition of acid reaching different protonation states of the porphyrins

Porphyrin/solvent	Added MSA/main protonation state	*A* (*λ*/nm (*ε* × 10^3^ M^−1^ cm^−1^))	Fluorescence (*λ*/ nm)
B_4_ DMSO : DCM (1 : 2)	No MSA	447 (88.2); 588 (16.9); 675 (9.5)	
	+ 1.4 µmol B_4_H_2_^2+^	403 (59.5); 478 (75.3), 574 (28.3), 850 (62.5)	710
	+ 872 µmol B_4_H_4_^4+^	433 (74.5), 502 (28.6); 768 (33.1)	—
	+ 2.2 mmol B_4_H_6_^6+^	441 (183.1); 654 (22.9)	685
AB_3_ DMSO : DCM (1 : 1)	No MSA	443 (138.0); 584 (24.2); 672 (13.0)	706
	+ 1.1 µmol AB_3_H_2_^2+^	426 (82.3); 488 (91.4); 589 (34.7), 828 (117.0)	—
	+ 844 µmol AB_3_H_4_^4+^	445 (124.0); 772 (63.0)	—
	+ 6.15 mmol AB_3_H_5_^5+^	449 (216.7); 668 (51.8)	707
*trans*-A_2_B_2_ DMSO	No MSA	416 (134.8); 581 (17.5); 665 (10.0)	703
	+ 2.2 µmol *trans*-A_2_B_2_H_2_^2+^	442 (118.7); 515 (51.5); 797 (66.7)	—
	+ 620 µmol *trans-*A_2_B_2_H_3_^3+^	450 (130.9); 517 (31.8); 759 (51.1)	721
	+ 5.4 mmol *trans*-A_2_B_2_H_4_^4+^	458 (186.2); 679 (38.0)	723
*cis*-A_2_B_2_ DMSO	No MSA	419 (103.7); 581 (21.9); 668 (14.6)	698
	+ 2 µmol *cis*-A_2_B_2_H_2_^2+^	440 (98.2); 796 (67.8)	—
	+ 705 µmol *cis-*A_2_B_2_H_3_^3+^	451 (117.5); 767 (50.7)	718
	+ 6 mmol *cis*-A_2_B_2_H_4_^4+^	493 (151.0); 681 (36.5)	719
NH_2_-AB_3_ DMSO : DCM (1 : 1)	No MSA	445 (124.7); 588 (18.8); 675 (9.2)	710
	+ 1.3 µmol NH_2_-AB_3_H_2_^2+^	400 (67.1); 473 (111.0); 843 (99.9)	—
	+ 852 µmol NH_2_-AB_3_H_4_^4+^	425 (108.9), 496 (45.4), 781 (55.0)	—
	+ 6 mmol NH_2_-AB_3_H_6_^6+^	442 (283.9); 652 (30.3)	685

For AB_3_, the nonprotonated porphyrin is characterized by a Soret band at 433 nm and two Q bands at 584 and 672 nm. Interestingly, differences in the absorption spectrum were observed when AB_3_ was measured in a DCM : DMSO (1 : 1) mixture (Fig. S30a) and in pure DMSO (Fig. 3). In DMSO, the Soret band split into two bands at 414 and 445 nm. These spectral changes may be associated with the higher polarity and coordinating ability of DMSO compared to DCM, which may influence the electronic structure and transition energies of the porphyrin, resulting in the observed changes in the Soret region.^42^ In addition, during sample preparation, AB_3_ was found to be more readily soluble in DCM than in DMSO. The lower solubility of AB_3_ in DMSO may therefore favor intermolecular interactions and aggregation, which could also contribute to the observed spectral changes.^43^ Upon addition of MSA, the absorption spectrum of AB_3_ undergoes pronounced changes, including the formation of a strong, broad band at 828 nm and further splitting of the Soret band into two bands at 426 and 488 nm. These spectral changes are consistent with protonation of the inner pyrrolic nitrogens and the formation of the diprotonated species, AB_3_H_2_^2+^, as observed for the two previously discussed porphyrins. Further addition of MSA results in a hypsochromic shift of the band from 828 to 772 nm, accompanied by a decrease in its intensity, with the aforementioned molar absorption coefficient decrease. At the same time, the two Soret bands merge into a single, narrower band at 445 nm. These changes can be attributed to further protonation of two *N*,*N*-diethylaniline groups in addition to the pyrrolic nitrogens, presumably resulting in the formation of AB_3_H_4_^4+^. Upon further addition of MSA, protonation of the remaining unprotonated *N*,*N*-diethylaniline group leads to the formation of fully protonated specie, AB_3_H_5_^5+^. This is accompanied by the formation of a strong, narrow Soret band at 449 nm, with a molar absorption coefficient of 219 000 M^−1^ cm^−1^, as well as the appearance of a new Q band at 668 nm with a molar absorption coefficient of 51 800 M^−1^ cm^−1^.

**Fig. 3:**
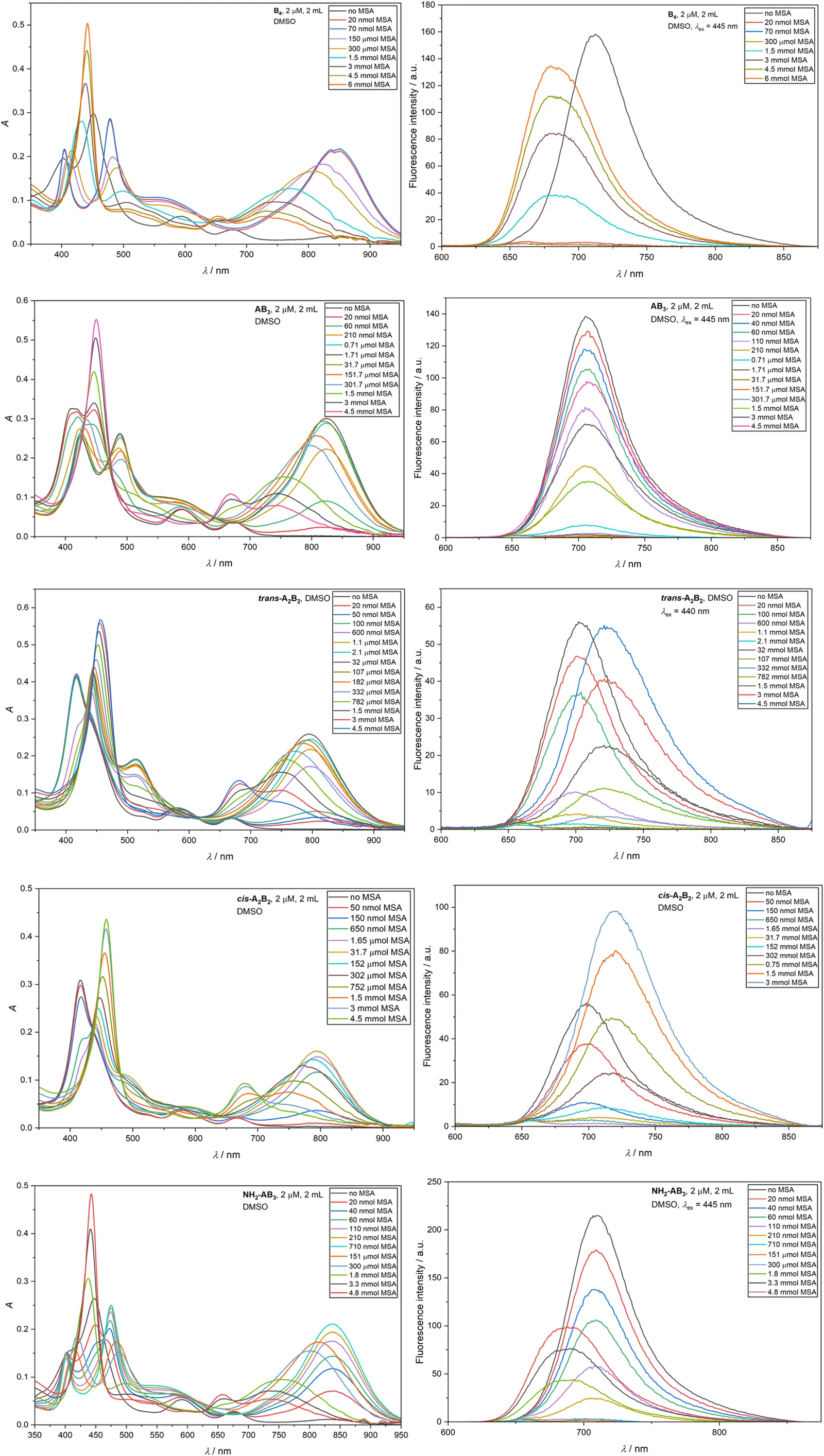
Absorption (left) and corresponding fluorescence (right) spectra of porphyrins B_4_, AB_3_, *trans*-A_2_B_2_, *cis*-A_2_B_2_, and NH_2_-AB_3_ (all in 2 µM concentrations) measured in DMSO and after addition of MSA at room temperature.

The two porphyrins bearing two *N*,*N*-diethylaniline substituents, *trans*-A_2_B_2_ and *cis*-A_2_B_2_, exhibit characteristic absorption spectra with a strong Soret band and two distinct Q bands at 416, 581 and 665 nm for the *trans* isomer and at 419, 581 and 668 nm for the *cis* isomer. The formation of a strong, broad band at 797 nm for *trans*-A_2_B_2_ and 796 nm for *cis*-A_2_B_2_, accompanied by a bathochromic shift of the Soret band from 416 to 442 nm and from 419 to 440 nm, respectively, is consistent with protonation of the inner pyrrolic nitrogens and formation of *trans*-A_2_B_2_H_2_^2+^ and *cis*-A_2_B_2_H_2_^2+^ upon addition of MSA. Further addition of MSA presumably results also in protonation of one *N*,*N*-diethylaniline substituent and leads to the formation of *trans*-A_2_B_2_H_3_^3+^ and *cis*-A_2_B_2_H_3_^3+^. This process is accompanied by a further bathochromic shift of the Soret band to 450 nm and an increase in its molar absorption coefficient, from 118 700 to 130 900 M^−1^ cm^−1^ for the *trans* isomer and from 98 200 to 117 000 M^−1^ cm^−1^ for the *cis* isomer. At the same time, the broad band in the region of 800 nm undergoes a hypsochromic and hypochromic shift, moving to 759 nm for the *trans* isomer and 767 nm for the *cis* isomer. Finally, protonation of the remaining *N*,*N*-diethylaniline substituent results in the fully protonated species, *trans*-A_2_B_2_H_4_^4+^ and *cis*-A_2_B_2_H_4_^4+^. This is accompanied by a further bathochromic shift and increase in the intensity of the Soret band, with a substantially larger spectral shift observed for the *cis* isomer. In the case of the *cis* isomer, the Soret maximum shifts from 450 to 493 nm, whereas the *trans* isomer exhibits a comparatively smaller shift of ∼10 nm. The formation of the fully protonated species is also supported by the appearance of a new Q band at approximately 680 nm, with maxima at 679 nm for the *trans* isomer and 681 nm for the *cis* isomer.

It is important to note that the number of *N*,*N*-diethylaminophenyl *meso*-substituents influences the basicity of the investigated porphyrins, and therefore the formation of hyperporphyrin species. Porphyrins bearing a larger number of electron-donating substituents, namely B_4_ and NH_2_-AB_3_, exhibited pronounced hyperporphyrin features upon protonation, characterized by a strong and broad band in the region of 850 nm and the largest bathochromic shifts observed among the investigated porphyrins. These spectral changes were already observed upon addition of relatively small amounts of MSA (60–100 nmol). In the case of AB_3_, an intense band at 828 nm was observed, with the characteristic hyperporphyrin spectral features becoming apparent after the addition of approximately 300 nmol of MSA. In contrast, the *trans*-A_2_B_2_ and *cis*-A_2_B_2_ porphyrins, containing two electron-donating *N*,*N*-diethylaminophenyl substituents, exhibited the corresponding hyperporphyrin effects only after the addition of approximately 1 µmol of MSA.

A similar trend was observed for the fully protonated species. The position of the lowest-energy absorption band in the 650–700 nm region varied systematically with the number of electron-donating substituents, with maxima observed at 652, 654, 668, 679 and 681 nm for NH_2_-AB_3_H_6_^6+^, B_4_H_6_^6+^, AB_3_H_5_^5+^, *trans*-A_2_B_2_H_4_^4+^ and *cis*-A_2_B_2_H_4_^4+^, respectively.

The fluorescence spectra of all tested porphyrins, together with their corresponding absorption spectra, are presented in Fig. 3. For all the porphyrins, protonation and the consequent formation of hyperporphyrin species resulted in the complete loss of fluorescence. There seems to be only one study in the literature that examined the impact of pH and the hyperporphyrin effect on the emission of *meso*-tetraphenylporphyrins, and it supports our finding and suggests that a stronger drop in fluorescence is related to a stronger hyperporphyrin effect.^44^ Moreover, in our case, upon further addition of MSA until complete protonation, a recovery of fluorescence was observed for all the porphyrins.

Due to differences in the basicity of the porphyrins, which are related to the number of 4-(*N*,*N*-diethylamino)phenyl and aminophenyl electron-donating groups, the amount of MSA required for fluorescence “recovery” varied among the investigated compounds. For B_4_ and NH_2_-AB_3_, fluorescence “recovery” was observed only after the amount of added MSA exceeded 1 mmol. Furthermore, for both porphyrins, fluorescence recovery was accompanied by a hypsochromic shift of approximately 25 nm (Table 1 and Fig. S34). In the case of AB_3_, fluorescence recovery was observed after the addition of more than 500 µmol of MSA, accompanied by broadening of the fluorescence band without a significant shift in the maximum of the fluorescence band (Table 1 and Fig. S34).

For *trans*-A_2_B_2_ and *cis*-A_2_B_2_, fluorescence recovery was observed after the addition of 332 and 152 µmol of MSA, respectively. Interestingly, in both cases, fluorescence recovery was accompanied by a bathochromic shift of approximately 20 nm (Table 1 and Fig. S34).

### 3.4 ROS production by DPBF photodegradation

In this work, DPBF photodegradation was selected as a method to evaluate ROS generation for several reasons. First, DPBF is soluble and stable in polar aprotic solvents such as DMSO,^45^ which was used in all the acid titration experiments on the porphyrins. Second, DPBF photodegradation is a widely employed method for measuring ^1^O_2_ generation, through the [4 + 2] cycloaddition reaction with ^1^O_2_ and other ROS, resulting in products that do not interfere with the spectroscopic monitoring performed during the irradiation conditions in this study (*λ* = 657 nm).^46^ Moreover, DPBF does not exhibit appreciable absorption or emission at the irradiation wavelength, minimizing potential interference with the photophysical measurements. Finally, although DPBF is highly reactive towards singlet oxygen and is therefore frequently employed as a singlet oxygen scavenger, it is not completely selective for ^1^O_2_. In fact, DPBF can also undergo reactions with other ROS, such as those associated with type I photochemical pathways, so DPBF photodegradation provides an indication of the overall ROS generation rather than an unambiguous measure of singlet oxygen production alone.^47,48^ This latter characteristic was particularly advantageous for investigating the influence of the hyperporphyrin effect on overall ^1^O_2_/ROS generation, which could indicate the potential efficacy of the tested porphyrins as PSs in PDT in the environments with different acidity. At the same time, it is necessary to consider the potential role of DMSO as a scavenger of certain ROS, mainly hydroxyl radicals when interpreting the results of DPBF photodegradation in this solvent.^49^ DMSO reacts with hydroxyl radicals to form methane sulfinic acid^49^ and with hypochlorite to form dimethyl sulfone.^50^ Therefore, any contribution of hydroxyl radicals to the total ROS production could be masked by the scavenging effect of DMSO.

The irradiation wavelength of the LED-based light setup used for ROS production in this study was specifically adjusted to match the wavelength of the last Q band of the porphyrins in their nonprotonated form. Moreover, the porphyrins' absorbance at 675 nm stayed very similar throughout the study upon titration with MSA, therefore also in their protonated forms. Only when 3 mmol of acid were added, that is, when the porphyrins were in their fully protonated form, did the absorption increase noticeably in all the porphyrins tested. The number of photons absorbed by porphyrins and their protonated species during irradiation with red light (675 nm, 41 mW cm^−2^, 24.6 J cm^−2^) was also calculated and shows relatively close values (Table S3).

When comparing ROS production in the porphyrins' nonprotonated state, it can be noticed that the number of electron-donating groups in the porphyrin structure is related to higher production of ^1^O_2_/ROS. Porphyrins B_4_ and NH_2_-AB_3_, with four aminophenyl *meso*-substituents, showed the highest ^1^O_2_/ROS production with the kinetic constant (*k*) of DPBF photodegradation of 1.68 × 10^−1^ ± 2.4 × 10^−3^ and 1.20 × 10^−1^ ± 2.5 × 10^−3^, respectively, while *trans*-A_2_B_2_ and *cis*-A_2_B_2_ showed the lowest ROS production with the *k* values of 5.15 × 10^−2^ ± 2.0 × 10^−4^ and 6.76 × 10^−2^ ± 1.0 × 10^−4^, respectively (Table 2 and Fig. S38). Upon protonation by the addition of 30 µmol of MSA, which induces the strong hyperporphyrin effect (seen as strong absorption maxima at ∼790–800 nm) (Fig. 4, absorption spectra, orange line) and results in the formation of species with the inner pyrrole nitrogens protonated (B_4_H_2_^2+^, AB_3_H_2_^2+^, *trans*-A_2_B_2_H_2_^2+^, *cis*-A_2_B_2_H_2_^2+^, and NH_2_-AB_3_H_2_^2+^), almost complete suppression of DPBF photodegradation was observed for all tested porphyrins. Although the observed ROS production upon protonation of inner pyrroles is low, a small shift in the order of DPBF photodegradation intensity among porphyrins was observed, where *trans*-A_2_B_2_H_2_^2+^and *cis*-A_2_B_2_H_2_^2+^ showed the strongest DPBF photodegradation in comparison to other porphyrins, with the observed kinetic constant of photodegradation 8.34 × 10^−3^ ± 1.23 × 10^−6^ for the *trans* and 1.75 × 10^−2^ ± 3.15 × 10^−5^ for the *cis* isomer. Moreover, the trend continued after the addition of 300 µmol of MSA, where porphyrins B_4_ and NH_2_-AB_3_, are still demonstrating a strong hyperporphyrin effect as B_4_H_4_^4+^ and NH_2_-AB_3_H_4_^4+^ species. Therefore, their impact on DPBF photodegradation is still negligible, while markedly recovering for the *cis*- and *trans*-A_2_B_2_ that underwent full protonation. For all five porphyrins, the kinetic constant in the third stage (+300 µmol MSA) ranked as follows: B_4_ < NH_2_-AB_3_ < AB_3_ < *trans*-A_2_B_2_ < *cis*-A_2_B_2_.

**Table 2:** The kinetic rate constant (*k*) of DPBF photodegradation in DMSO without porphyrins and in the presence of porphyrins and various amounts of MSA after irradiation with red light for 10 minutes (*λ* = 675 nm, 41 mW cm^−2^, light dose 24.6 J cm^−2^) at room temperature

*k*				
	DMSO	+ 30 µmol MSA	+ 300 µmol MSA	+ 3 mmol MSA
B_4_	1.68 × 10^−1^ ± 2.4 × 10^−3^	3.31 × 10^−3^ ± 2.9 × 10^−7^	4.34 × 10^−3^ ± 4.6 × 10^−5^	2.22 × 10^−2^ ± 4.5 × 10^−5^
AB_3_	1.12 × 10^−1^ ± 1.0 × 10^−4^	7.94 × 10^−3^ ± 2.12 × 10^−5^	7.97 × 10^−3^ ± 2.3 × 10^−5^	9.74 × 10^−2^ ± 2.1 × 10^−4^
*trans*-A_2_B_2_	5.15 × 10^−2^ ± 2.0 × 10^−4^	8.34 × 10^−3^ ± 1.23 × 10^−6^	2.18 × 10^−2^ ± 5.3 × 10^−5^	1.99 × 10^−1^ ± 2.9 × 10^−3^
*cis*-A_2_B_2_	6.76 × 10^−2^ ± 1.0 × 10^−4^	1.75 × 10^−2^ ± 3.15 × 10^−5^	4.07 × 10^−2^ ± 7.2 × 10^−4^	2.51 × 10^−1^ ± 1.2 × 10^−3^
NH_2_-AB_3_	1.20 × 10^−1^ ± 2.5 × 10^−3^	7.23 × 10^−3^ ± 9.3 × 10^−6^	6.83 × 10^−3^ ± 1.2 × 10^−5^	6.59 × 10^−2^ ± 5.5 × 10^−5^

**Fig. 4:**
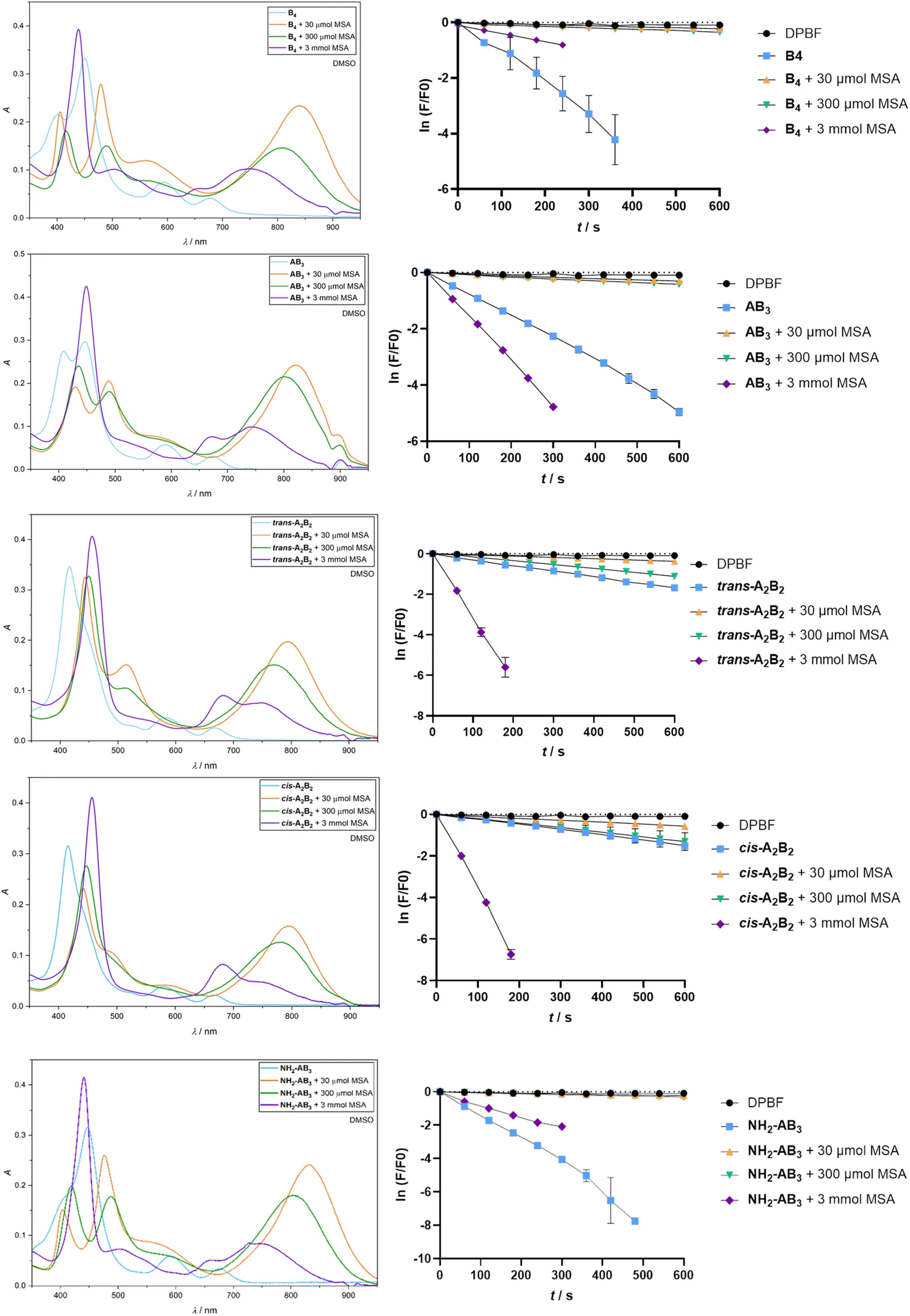
Photodegradation kinetics of DPBF upon ^1^O_2_/ROS production by B_4_, AB_3_, *trans*-A_2_B_2_, *cis*-A_2_B_2_ and NH_2_-AB_3_ (2 µM) in DMSO without or with the addition of 30 µmol, 300 µmol and 3 mmol of MSA (on the right) at room temperature compared with the absorption spectra under the same conditions (on the left). Photodegradation was measured as the decrease in fluorescence after irradiation for 10 min with red light (*λ* = 675 nm, 41 mW cm^−2^). All results are presented as mean (2), with standard deviation in error bars.

These results are consistent with the fluorescence spectra, where “recovery” of fluorescence for *cis*- and *trans*-A_2_B_2_ isomers was observed already after ∼300 µmol MSA had been added, in comparison with other porphyrins where “recovery” of the fluorescence spectra was observed only after ≥1 mmol of MSA had been added (Fig. 3). Therefore, the faster recovery might be due to the smaller number of the 4-(*N*,*N*-diethyl)aminophenyl groups that can contribute to the hyperporphyrin effect compared to B_4_ and AB_3_.

In the last part of the experiment, 3 mmol of MSA were added, so the porphyrins are expected to be mostly fully protonated (B_4_H_6_^6+^, AB_3_H_5_^5+^, *trans-*A_2_B_2_H_4_^4+^, *cis*-A_2_B_2_H_4_^4+^, and NH_2_-AB_3_H_6_^6+^) and hyperporphyrins are no longer present. In this case, the intensity of the DPBF photodegradation, which corresponds to the ^1^O_2_/ROS production, was again recovered (Fig. 4, purple line, Fig. S38). The order of the intensity of DPBF photodegradation among the porphyrins is, however, opposite to the starting, nonprotonated porphyrin trend, and is consistent with the order seen in the recovery pattern, B_4_ < NH_2_-AB_3_ < AB_3_ < *trans*-A_2_B_2_ < *cis*-A_2_B_2_, where the strongest kinetic constants, or ROS production, was detected for *trans*-A_2_B_2_ and *cis*-A_2_B_2_ with *k* of 1.99 × 10^−1^ ± 2.9 × 10^−3^ and 2.51 × 10^−1^ ± 1.2 × 10^−3^, respectively, and the lowest for B_4_ with *k* calculated 2.22 × 10^−2^ ± 4.5 × 10^−5^. It is important to notice that for B_4_ and NH_2_-AB_3_ ROS production of fully protonated porphyrins is smaller when compared with the starting (nonprotonated) porphyrin, for AB_3_ remained similar, whereas for *trans*-A_2_B_2_ and *cis*-A_2_B_2_ it is strongly increased (Table 2 and Fig. S38).

For a comparison, ^1^O_2_/ROS production was determined, under the same conditions, using 5,10,15,20-tetra(pyrid-3-yl)porphyrin (Py-B_4_). This porphyrin can be protonated at pyridyl nitrogens as well as inner (pyrrole) nitrogens, however it does not exhibit the hyperporphyrin effect, as demonstrated by the changes in its absorption spectra (Fig. S37), and previously described in the literature.^40^ In the nonprotonated form, Py-B_4_ showed significantly lower ^1^O_2_/ROS production than all *N*,*N*-diethylamino analogues, which can be mainly attributed to its lower absorption at the irradiation wavelength of 675 nm (Table S3). Upon addition of 30 mmol of MSA, the absorption spectrum of Py-B_4_ (Fig. S37, bottom left) shows only minor changes, mainly seen as the blue-shift of the Q bands. These changes coincide with lower ^1^O_2_/ROS production. Upon further addition of MSA in the amounts of 300 µmol and 3 mmol, the absorption at 675 nm increases (Table S3), as well as an enhanced ^1^O_2_/ROS production (Fig. S37 and S38). These results suggest that, unlike 4-(*N*,*N*-diethylaminophenyl) porphyrins, the observed differences in ^1^O_2_/ROS production of Py-B_4_ and its protonated forms can largely be attributed to the number of photons the porphyrin absorbs at the irradiation wavelength, while protonation itself seems to have only a small negative effect that cannot be attributed to the hyperporphyrin effect.

Singlet oxygen quantum yields (*Φ*_Δ_) of several representative porphyrin-based PSs reported in literature (Table S2) demonstrate that, even though these values can vary considerably depending on the experimental conditions, including the solvent and the method used to determine *Φ*_Δ_,^51,52^ the reported quantum yields for many porphyrin-type PSs generally fall within the range of 0.5–0.8. Although a direct comparison with our systems is not possible due to the experimental conditions, the structural similarities between the porphyrins in this study and previously reported PSs with these *Φ*_Δ_ values (Table S2) could indicate that the singlet oxygen quantum yields of the studied nonprotonated and fully protonated porphyrins may also be in the mentioned range. Several studies in the literature also indicated that hyperporphyrins could be interesting as PSs for PDT due to their high absorption in the far-red region of the EM spectrum and their ability to undergo photosensitized oxidation reactions,^27–29^ however, our results suggest a different perspective. The stronger the hyperporphyrin effect was, the stronger the observed suppression of ^1^O_2_/ROS production, thereby eliminating the possibility of causing cytotoxic effects in cells through PDT. Of note, using wavelengths longer than 800 nm is not useful for PDT because such photons do not have enough energy to excite the PS,^53^ but for example IR808 (MHI-148), a well-known near-infrared heptamethine cyanine dye, can be used both in anticancer photothermal therapy (PTT), in which case is excited by an 808 nm laser, as well as in PDT, when 660–670 nm is used, and it can also be used for fluorescence imaging.^54^ Hyperporphyrins in this study, however, strongly suppress both fluorescence and ^1^O_2_/ROS production. Due to a possible role of DMSO as a scavenger, it cannot be ruled out that hyperporphyrins, as well as other forms of these porphyrins (both the nonprotonated and fully protonated forms), produce hydroxyl radicals, which could be useful to test in future studies using a probe specific for that type of ROS and an appropriate solvent. Nevertheless, considering the probe used, most of the detected total ROS production can be attributed to ^1^O_2_, which is also considered the most important ROS for effective PDT.^17^ Therefore, the observed stronger drop in total ROS production in the case of a stronger hyperporphyrin effect points to a significant reduction in the PDT potential of this type of hyperporphyrins.

Similar to observations with fluorescence, a full recovery of ^1^O_2_/ROS production was observed after complete protonation of the porphyrins, probably due to the disappearance of hyperporphyrin species with charge-transfer interactions. However, the acidity needed to achieve complete protonation would not be suitable for *in vitro*, and especially not for *in vivo* applications. So, nonprotonated porphyrins, with three (AB_3_) and particularly with four (B_4_ and NH_2_-AB_3_) electron-donating aminophenyl groups, seem to be the best candidates for further research as PSs for PDT, at least in terms of ^1^O_2_/ROS production.

## 4. Conclusions

New 5,10,15,20-arylporphyrins with combinations of 4-(*N*,*N*-diethylamino)phenyl and either 4-(*N*-acetamido)phenyl or 4-aminophenyl group as *meso*-substituents were successfully synthesized and characterized, alongside the symmetric porphyrin bearing four 4-(*N*,*N*-diethylamino)phenyl groups. The formation of hyperporphyrins upon protonation was confirmed by ^1^H NMR and UV-vis spectroscopic titrations with MSA. It was also confirmed that the greater the number of stronger electron-donating groups, the greater the basicity of the internal nitrogens and the more significant the hyperporphyrin effect. Hyperporphyrins caused the loss of fluorescence, again with a stronger effect in porphyrins with a larger number of electron-donating groups, but with further protonation in all porphyrins, in accordance with the reduction of the hyperporphyrin effect, fluorescence increased. Interestingly, for the fully protonated porphyrins hypsochromic shift was observed for B_4_H_6_^6+^and NH_2_-AB_3_H_6_^6+^, while bathochromic shift for *trans-*A_2_B_2_H_4_^4+^ and *cis*-A_2_B_2_H_4_^4+^ and apparently no significant shift for AB_3_H_5_^5+^ compared to the emission spectra of the respective nonprotonated porphyrins.

ROS production, which is the most crucial for the efficacy of PDT was evaluated by photodegradation of DPBF, and hyperporphyrins proved to be inefficient ^1^O_2_/ROS producers in all cases. Curiously, isomers *trans* and *cis*-A_2_B_2_ demonstrated excellent ^1^O_2_/ROS production in the fully protonated form, as opposed to very low production by their nonprotonated forms. In contrast, nonprotonated porphyrins with the highest number of electron-donating groups, B_4_ and NH_2_-A_2_B_2_, showed efficient ^1^O_2_/ROS production, higher than their fully protonated forms, and considering also their relatively good absorption at 675 nm, they could be relevant for future PDT studies.

## Author contributions

Martina Mušković Lukić: formal analysis, investigation, methodology, validation, visualization, writing – original draft, writing – review & editing. Martin Lončarić: formal analysis, investigation, methodology, validation, visualization, writing – original draft, writing – review & editing. Melisa Ciglarić: writing, data analysis & visualisation. Ivana Lužaić: writing, data analysis & visualization. Dejana Vujnović: writing, data analysis & visualization. Nela Malatesti: conceptualization, formal analysis, investigation, methodology, project administration, supervision, visualization, writing – original draft, writing – review & editing. All authors read and approved the final manuscript.

## Conflicts of interest

There are no conflicts of interest to declare.

## Supplementary Material

RA-OLF-D6RA09445G-s001

## Data Availability

Data for this article are available at https://urn.nsk.hr/urn:nbn:hr:193:981032. Supplementary information (SI) is available. See DOI: https://doi.org/10.1039/d6ra09445g.
