# Supplementary material for: The effects of protonation of meso-tetraarylporphyrins bearing N,N-diethylamino groups and the resulting hyperporphyrins on fluorescence and ROS production
Source: RSC Adv. 2026 Nov 10. Online ahead of print. doi: 10.1039/d6ra09445g (PMC13750148; doi:10.1039/d6ra09445g)
Supplement: Supplementary file 1 [file RA-OLF-D6RA09445G-s001.pdf]

This journal is © The Royal Society of Chemistry

Supplementary information

for

The effects of protonation of *meso*-tetraarylporphyrins bearing *N,N*-diethylamino groups and the resulting hyperporphyrins on fluorescence and ROS production

Martina Mušković Lukić[a†], Martin Lončarić[b†], Melisa Ciglarić[a], Ivana Lužaić[a], Dejana Vujnović[a], Nela Malatesti[a*]

[a]Faculty of Biotechnology and Drug Development, University of Rijeka, Radmile Matejčić 2, Rijeka 51000, Croatia; [b]Laboratory for Photonics and Quantum Optics, Division of Experimental Physics, Ruđer Bošković Institute, Bijenička cesta 54, Zagreb 10000, Croatia; corresponding author, e-mail: nela.malatesti@biotech.uniri.hr

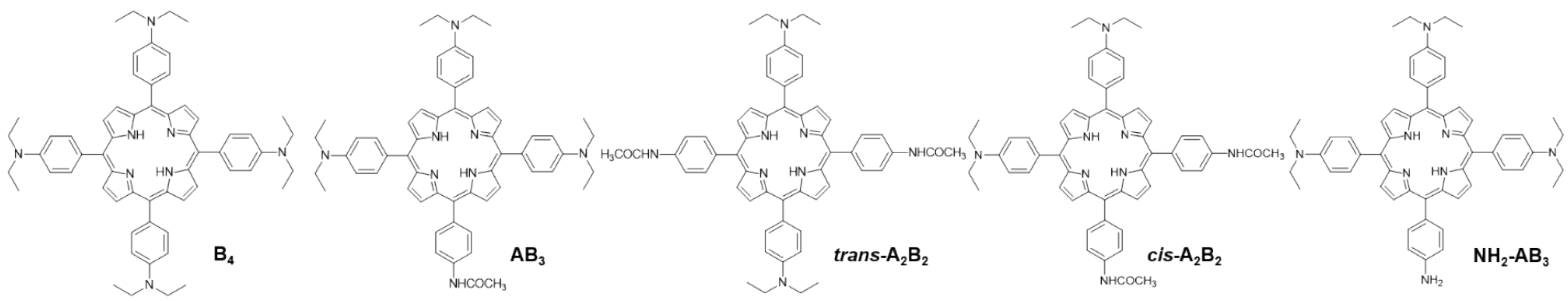

Content:

1. ^{1}H NMR and ^{13}C NMR spectra S2

2. Mass spectra S7

3. ^{1}H NMR spectra of acid titrations S11

4. Hyperporphyrin resonance forms S16

5. UV-Vis spectra of acid titrations S17

6. Fluorescence spectra of acid titrations S25

7. Light source S26

8. $^1O_2$/ROS production S27

1. ^{1}H NMR and ^{13}C NMR spectra

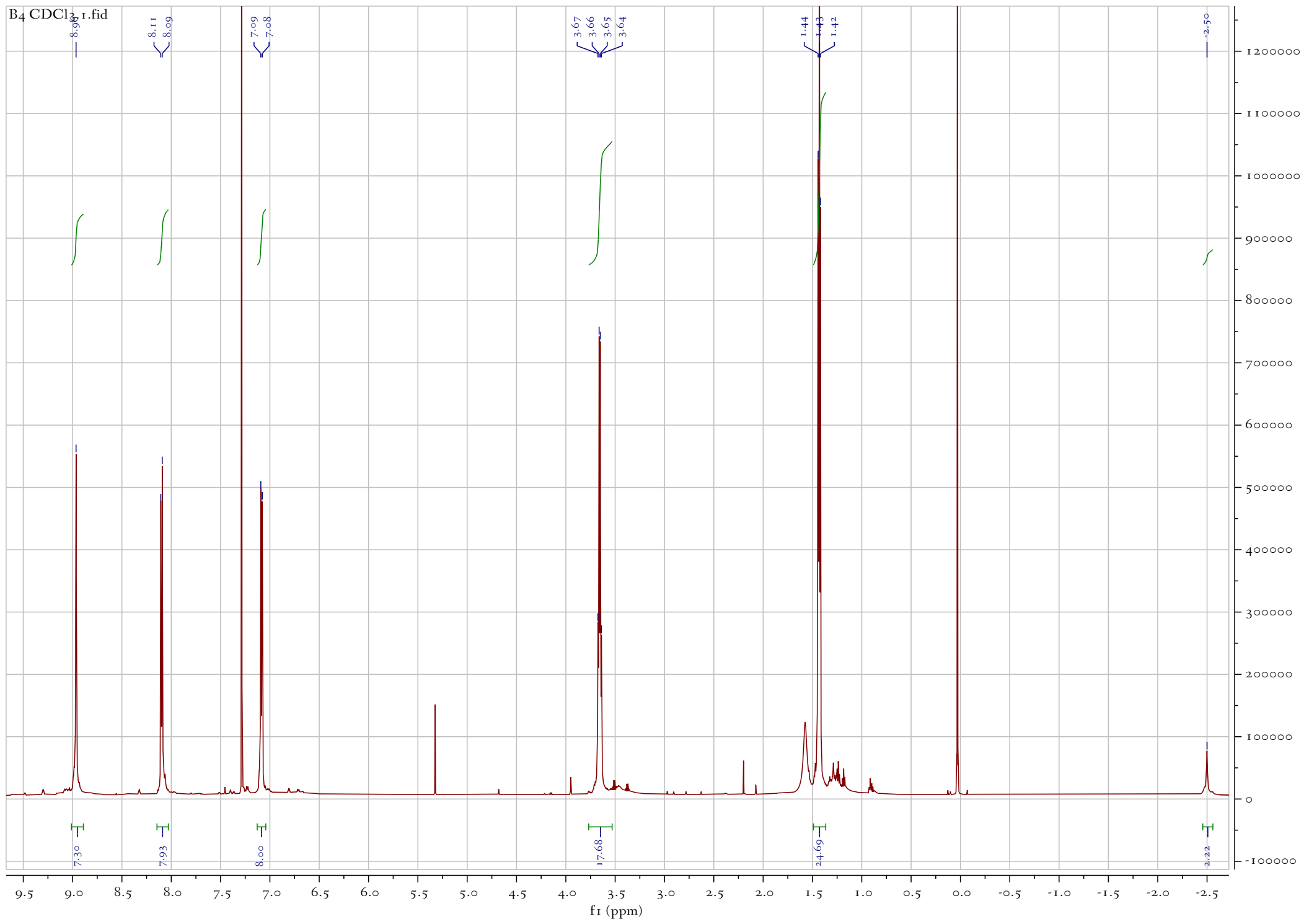

Fig. S1 ^{1}H NMR spectrum of **B_4** in $CDCl_3$ (solvent peaks: water at 1.58 ppm, and hexane at 0.91 and 1.19-1.30 ppm).

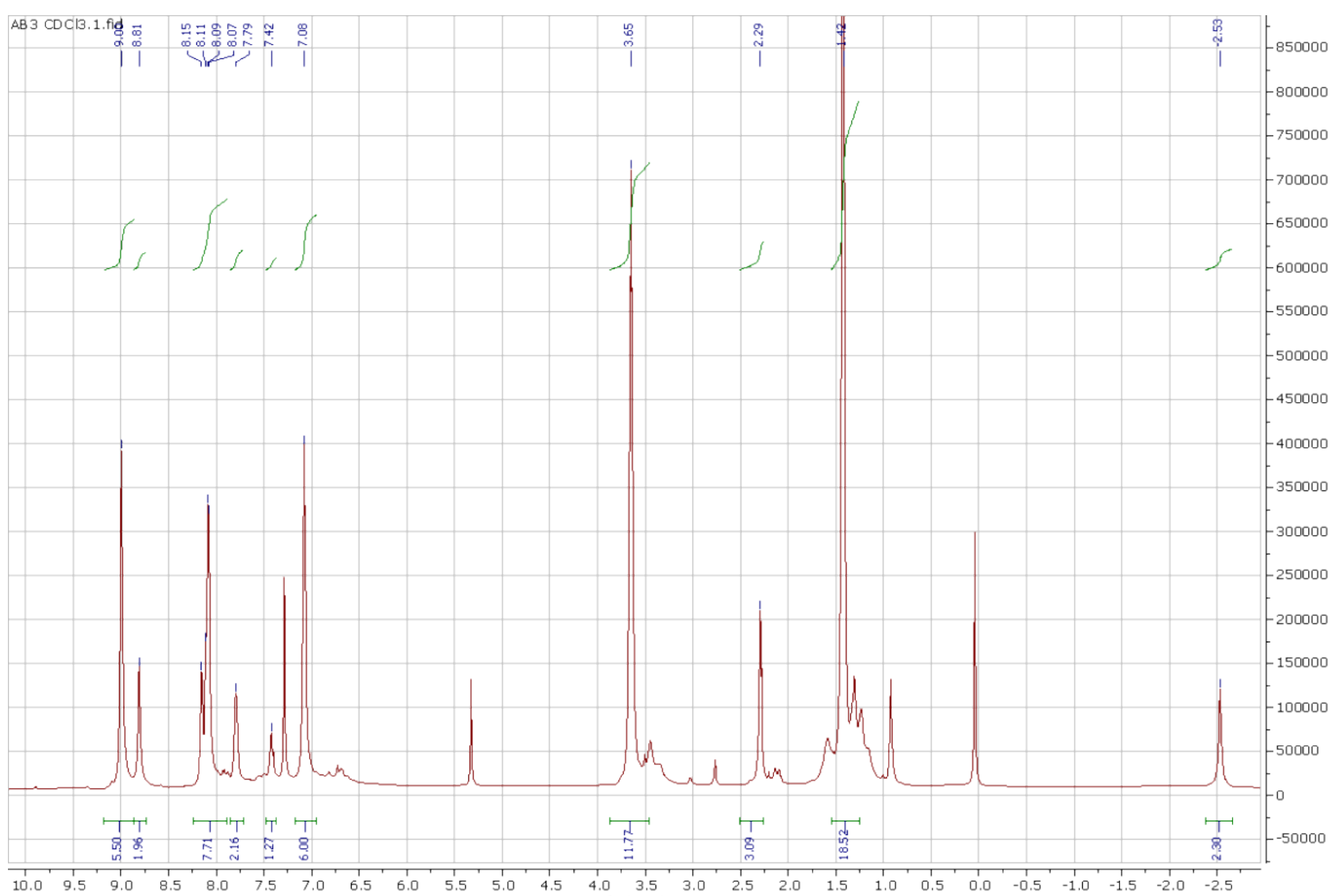

Fig. S2 ^{1}H NMR spectrum of **AB_3** in $CDCl_3$ (solvent peaks: water at 1.55 ppm, and hexane at 0.90 and 1.19-1.29 ppm).

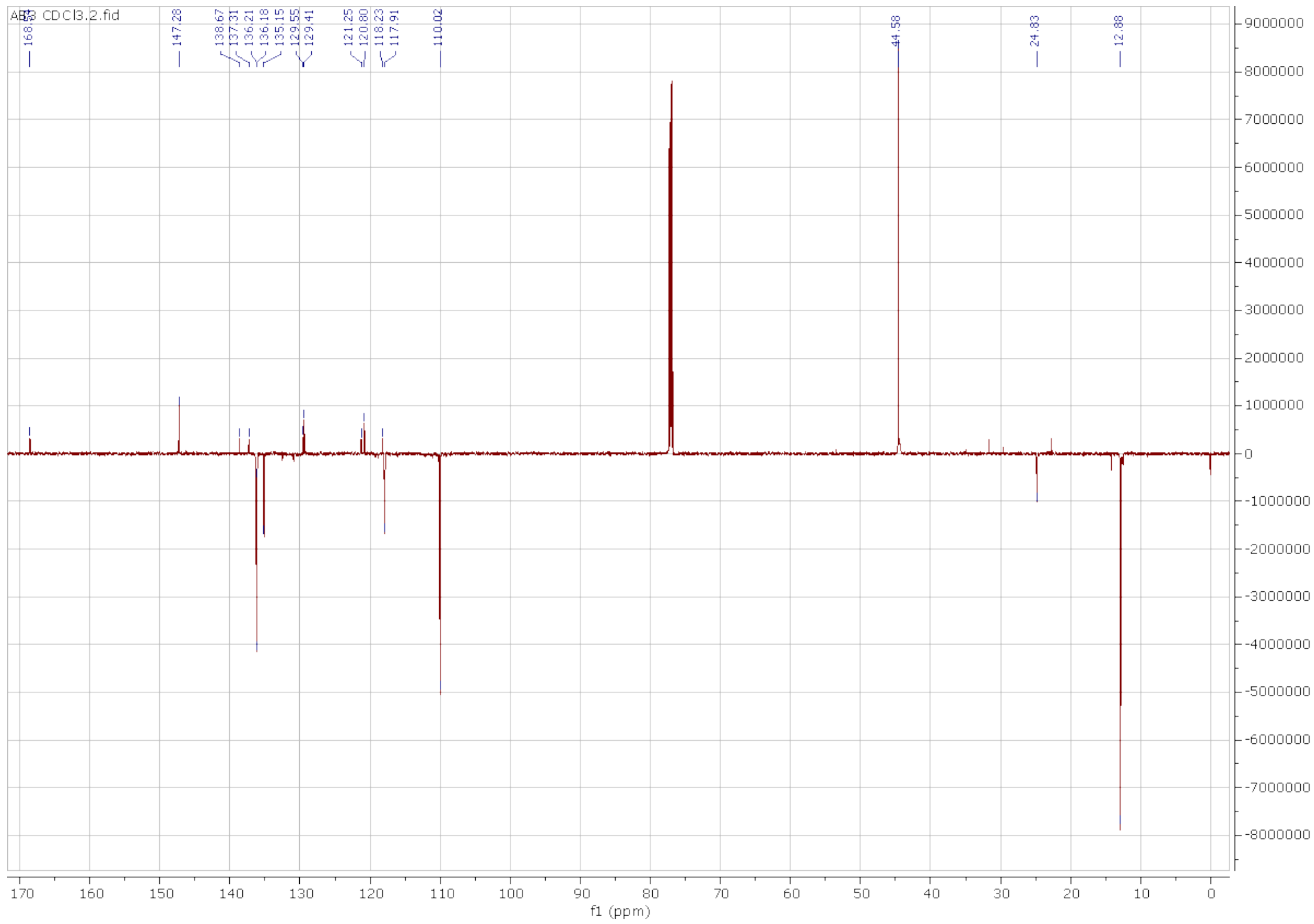

Fig. S3 ^{13}C NMR APT spectrum of $\mathbf{AB_3}$ in $CDCl_3$ (solvent peaks – hexane: 14.3, 22.9, 31.8 ppm).

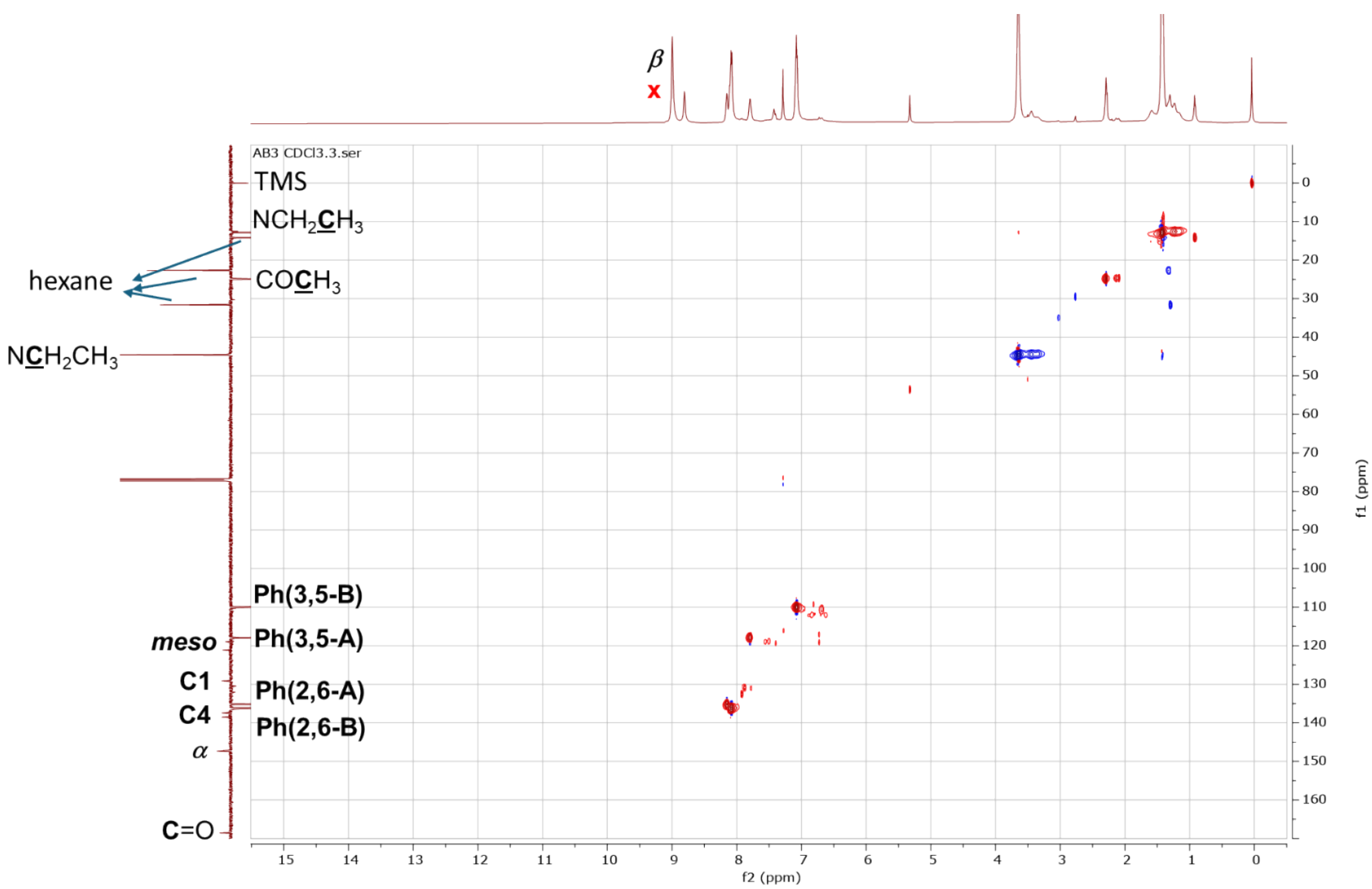

Fig. S4 HSQC (2D $^1H/^{13}C$ APT) NMR spectrum of $\mathbf{AB_3}$ in $CDCl_3$ with the assignments of ^{13}C signals.

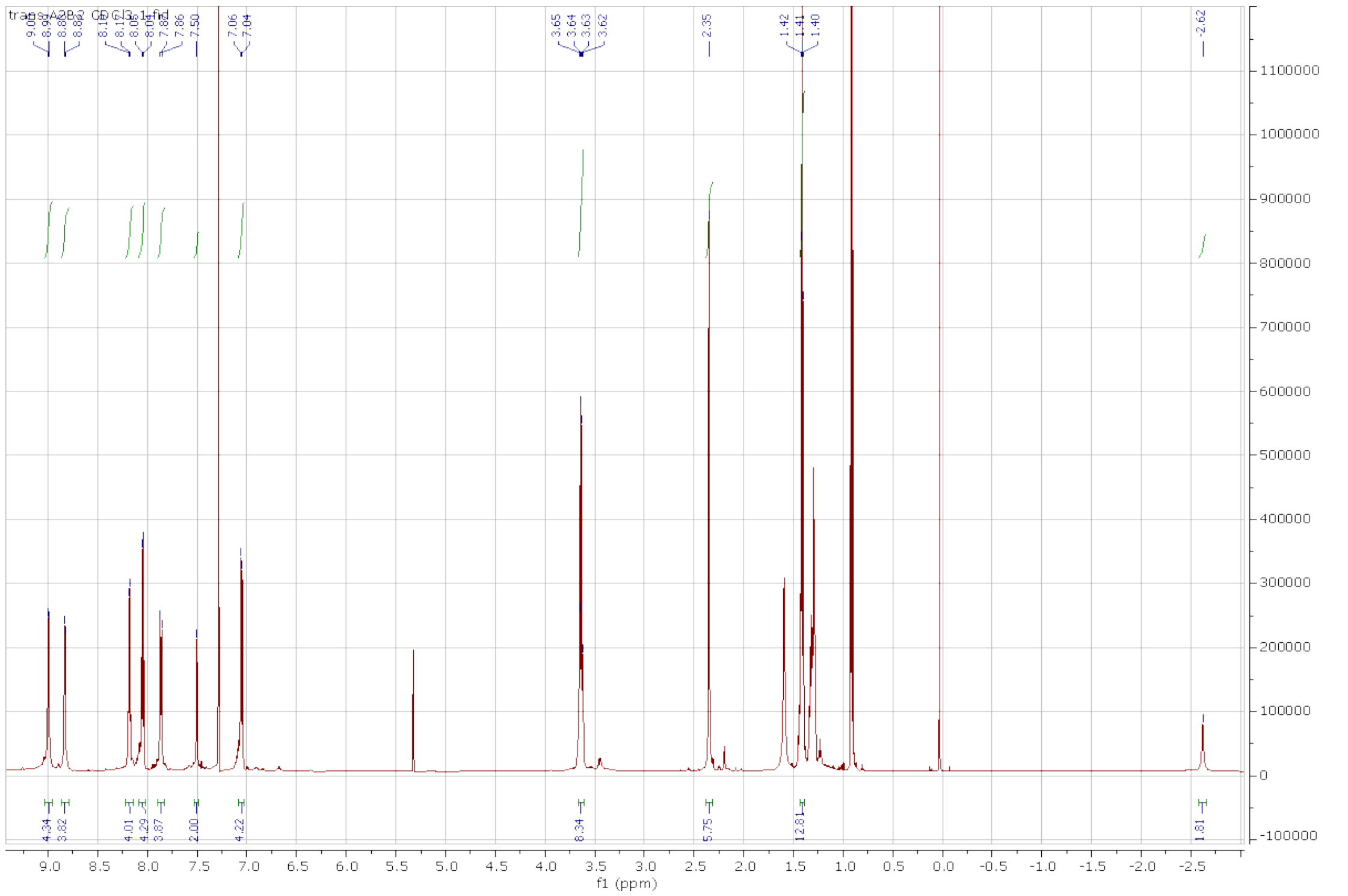

Fig. S5 ^{1}H NMR spectrum of ***trans*-A$_2$B$_2$** in $CDCl_3$ (solvent peaks: water at 1.59 ppm, and hexane at 0.91 and 1.26-1.32 ppm).

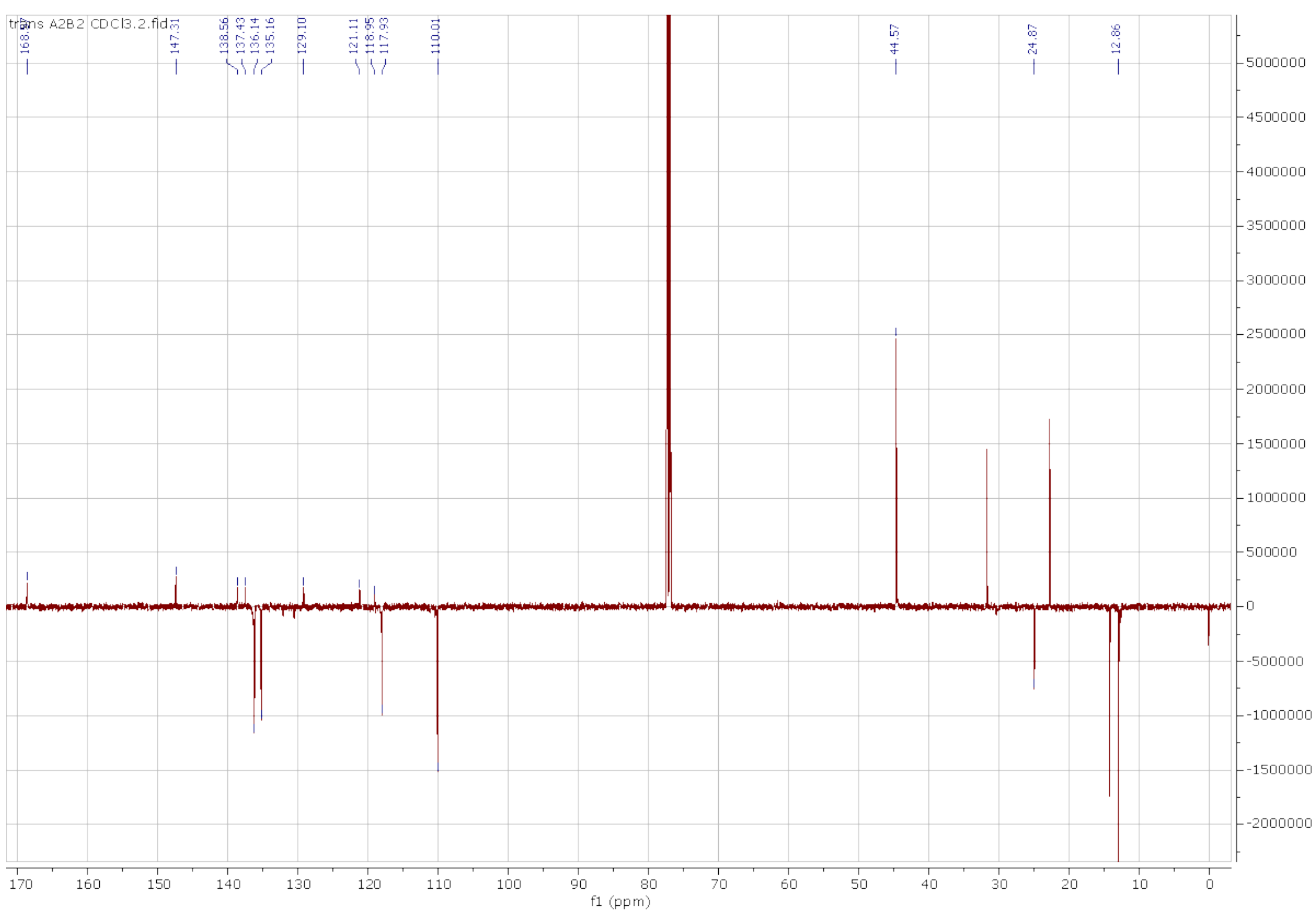

Fig. S6 ^{13}C NMR APT spectrum of ***trans*-A$_2$B$_2$** in $CDCl_3$ (solvent peaks − hexane: 14.2, 22.7, 31.6 ppm).

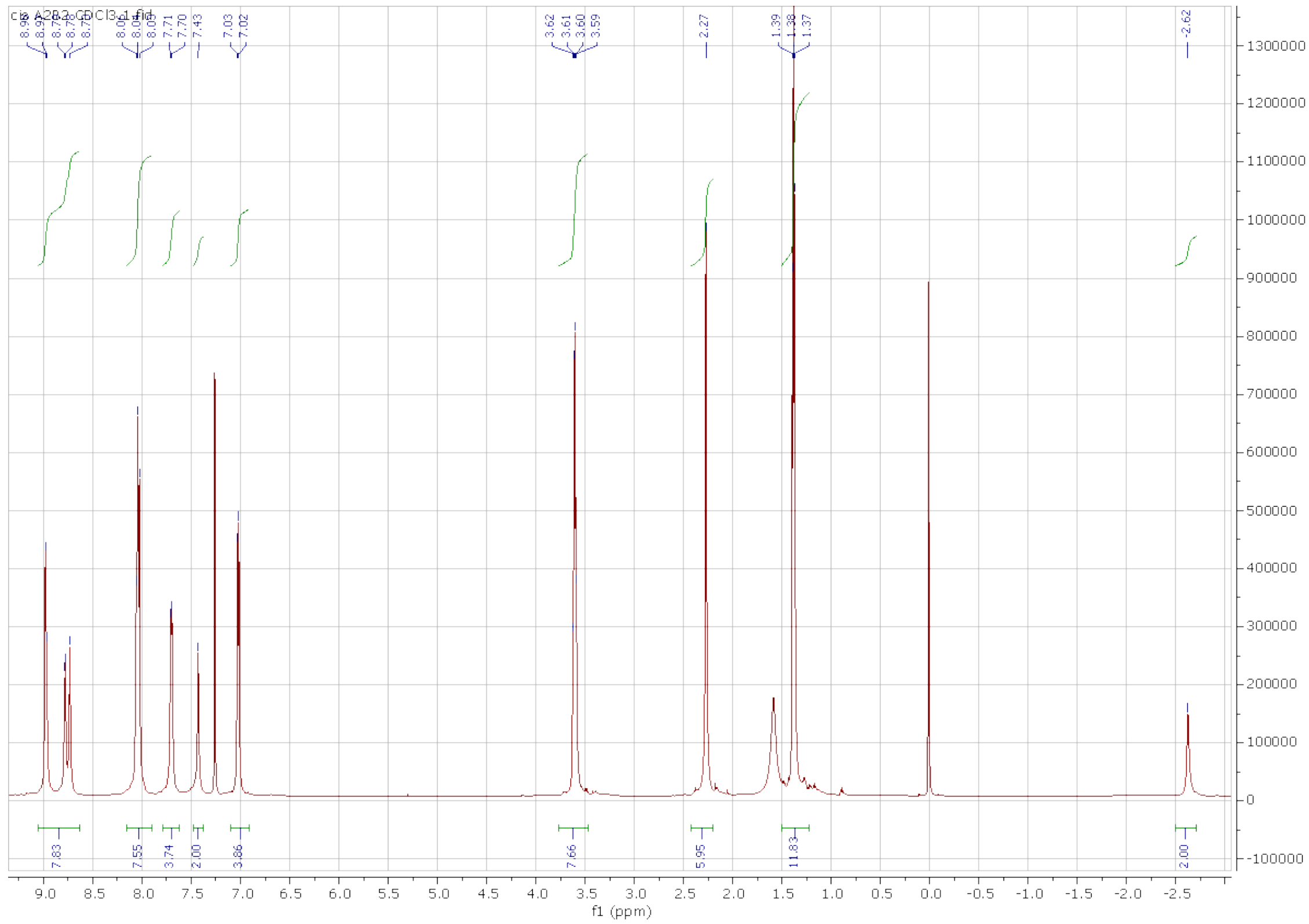

Fig. S7 ^{1}H NMR spectrum of ***cis*-A_2B_2** in $CDCl_3$ (solvent peak: water at 1.59 ppm).

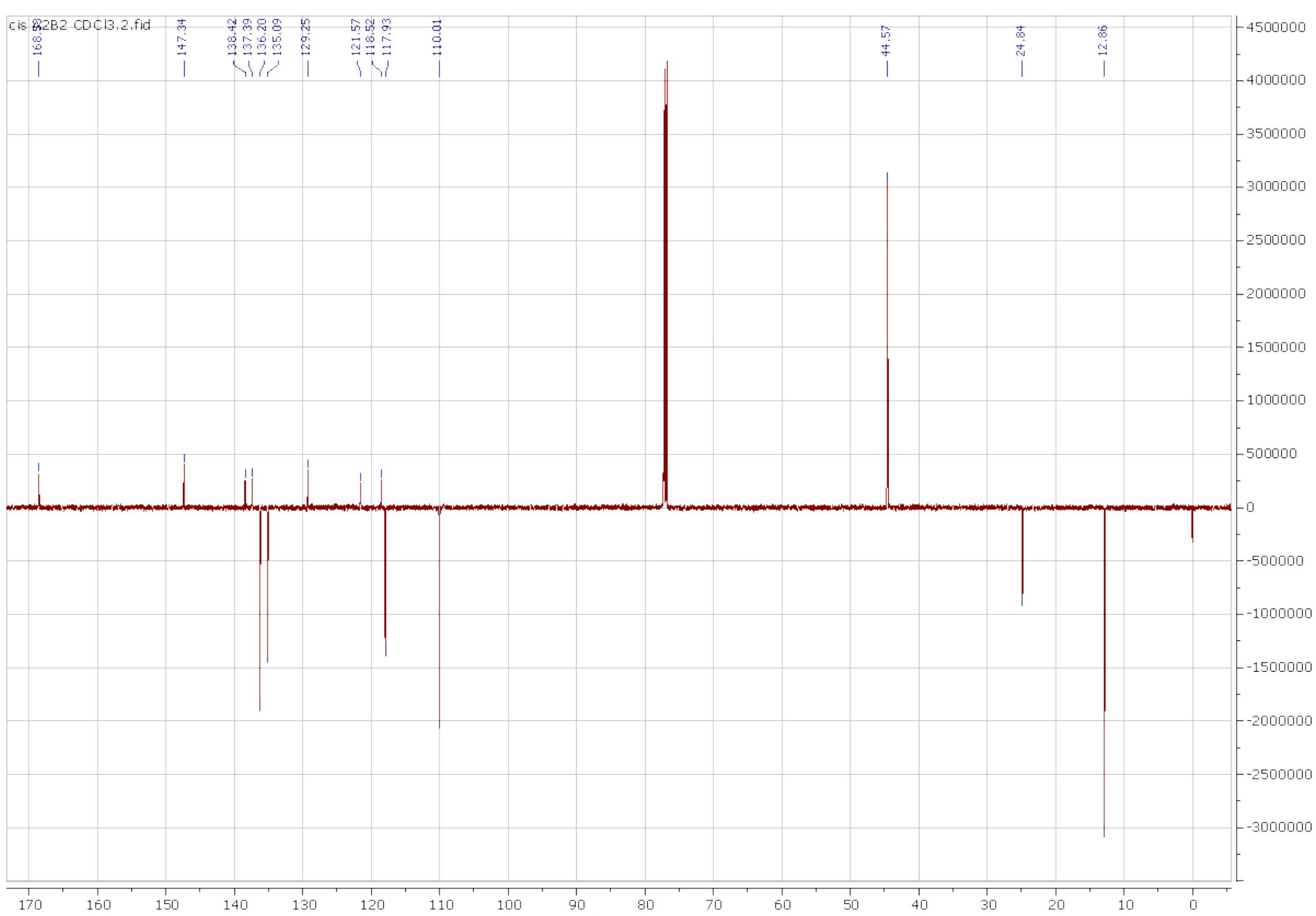

Fig. S8 ^{13}C NMR APT spectrum of ***cis*-A_2B_2** in $CDCl_3$.

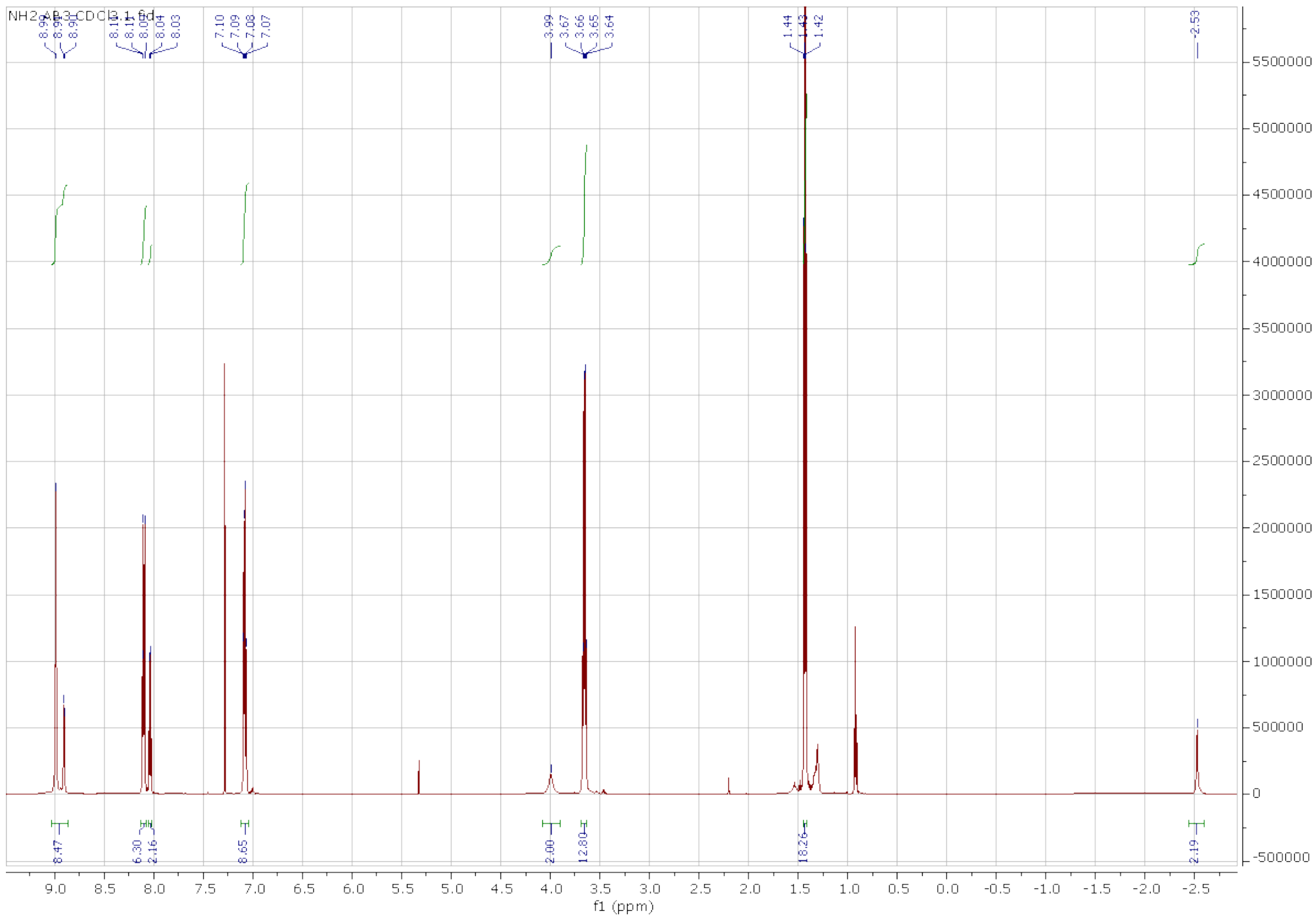

Fig. S9 ^{1}H NMR spectrum of **NH_2-AB_3** in $CDCl_3$ (solvent peaks: water at 1.55 ppm, and hexane at 0.92 and 1.28-1.35 ppm).

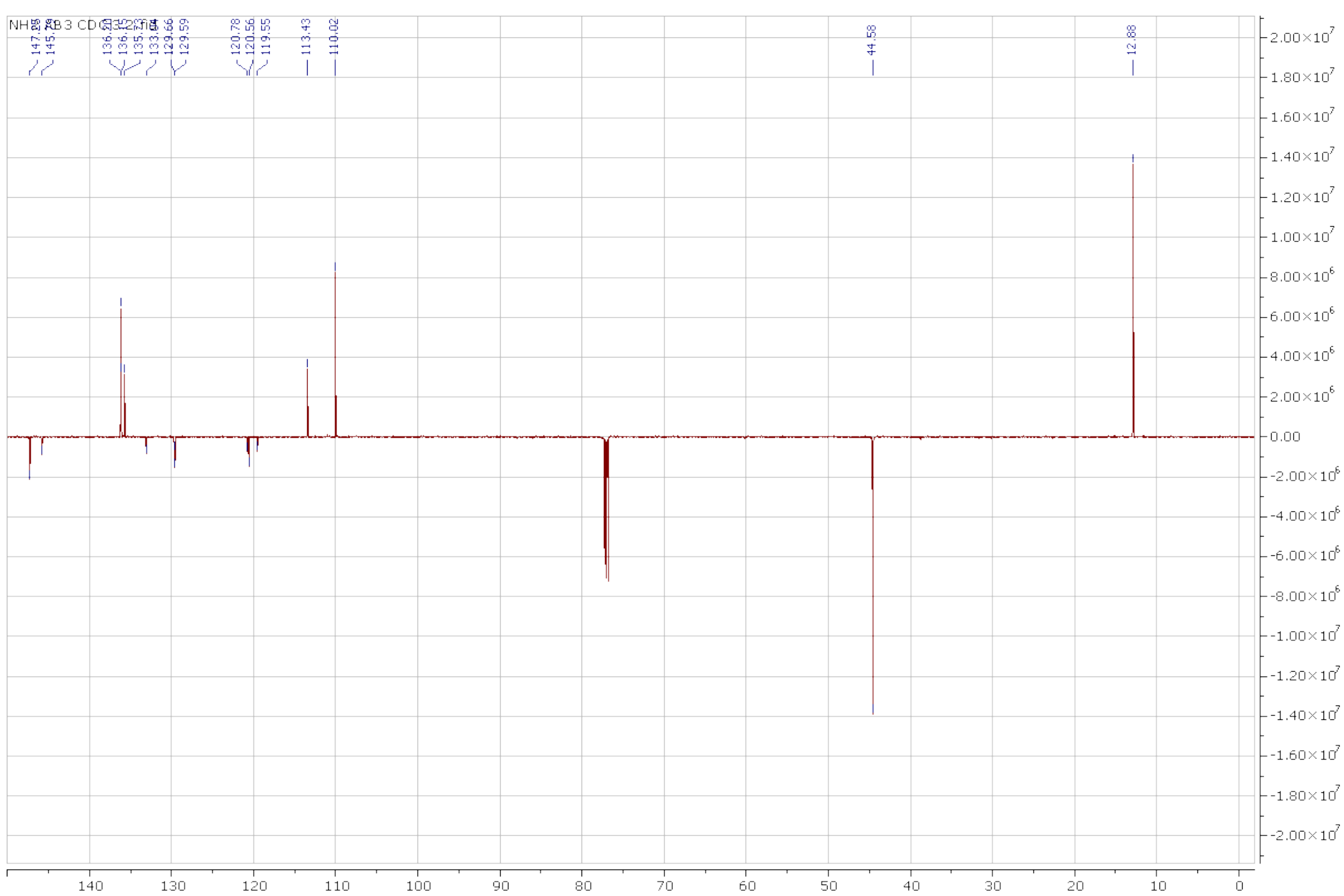

Fig. S10 ^{13}C NMR APT spectrum of **NH_2-AB_3** in $CDCl_3$.

2. Mass spectra

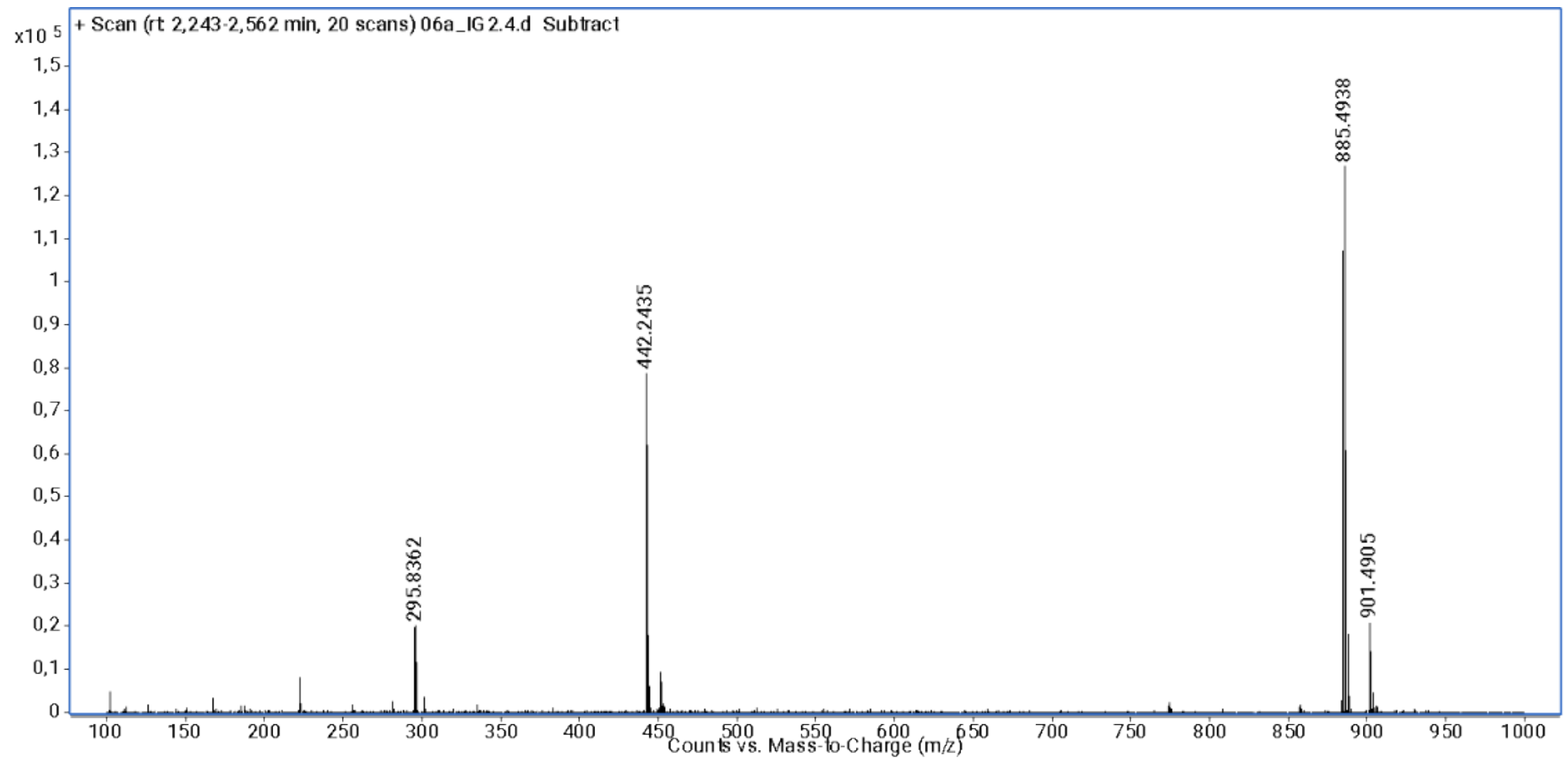

Fig. S11 Mass spectrum of AB_3 in the m/z range 100 – 1000.

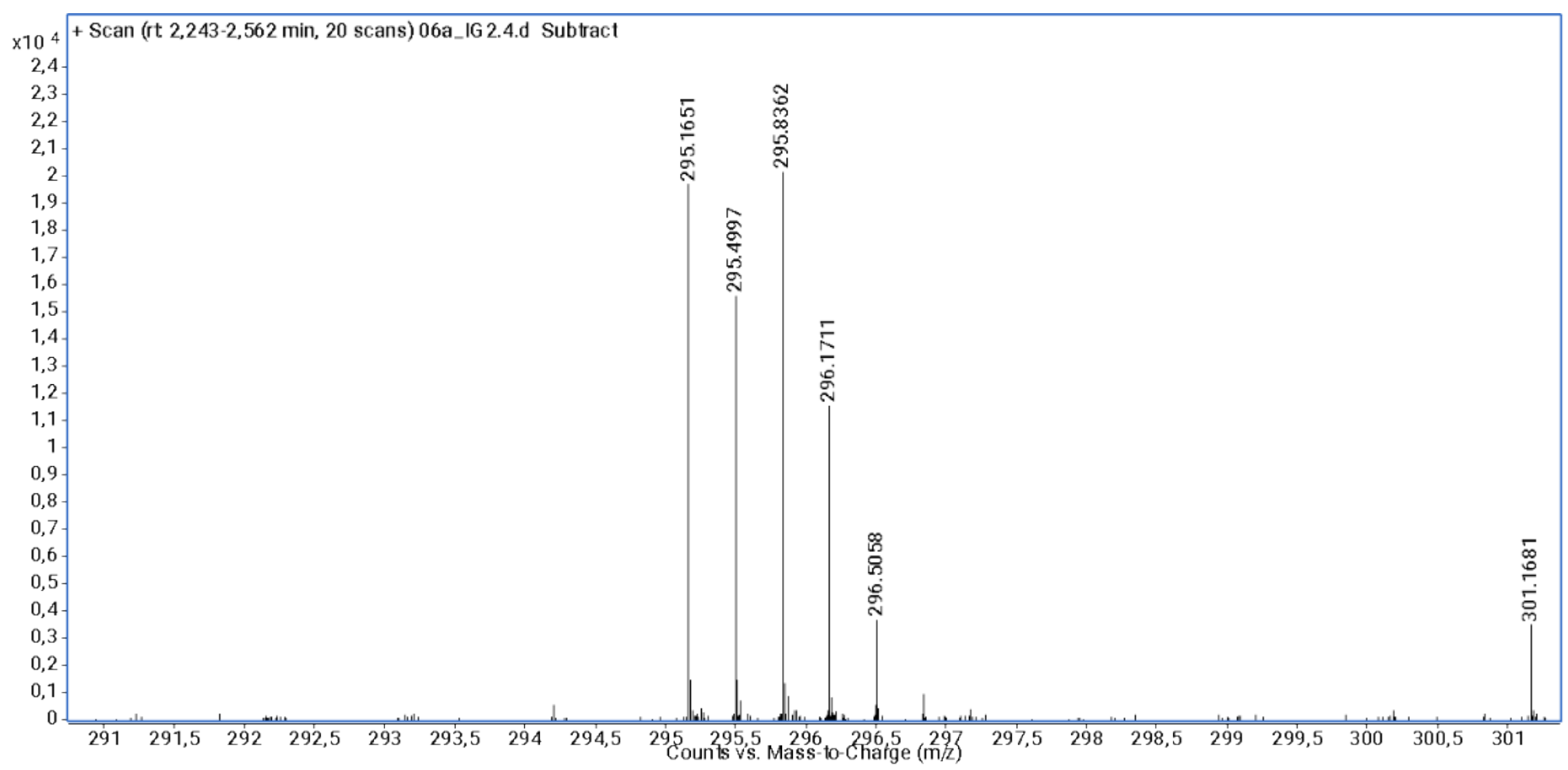

Fig. S12 Enlarged spectra from **Fig. S11** in the area m/z 291–301.

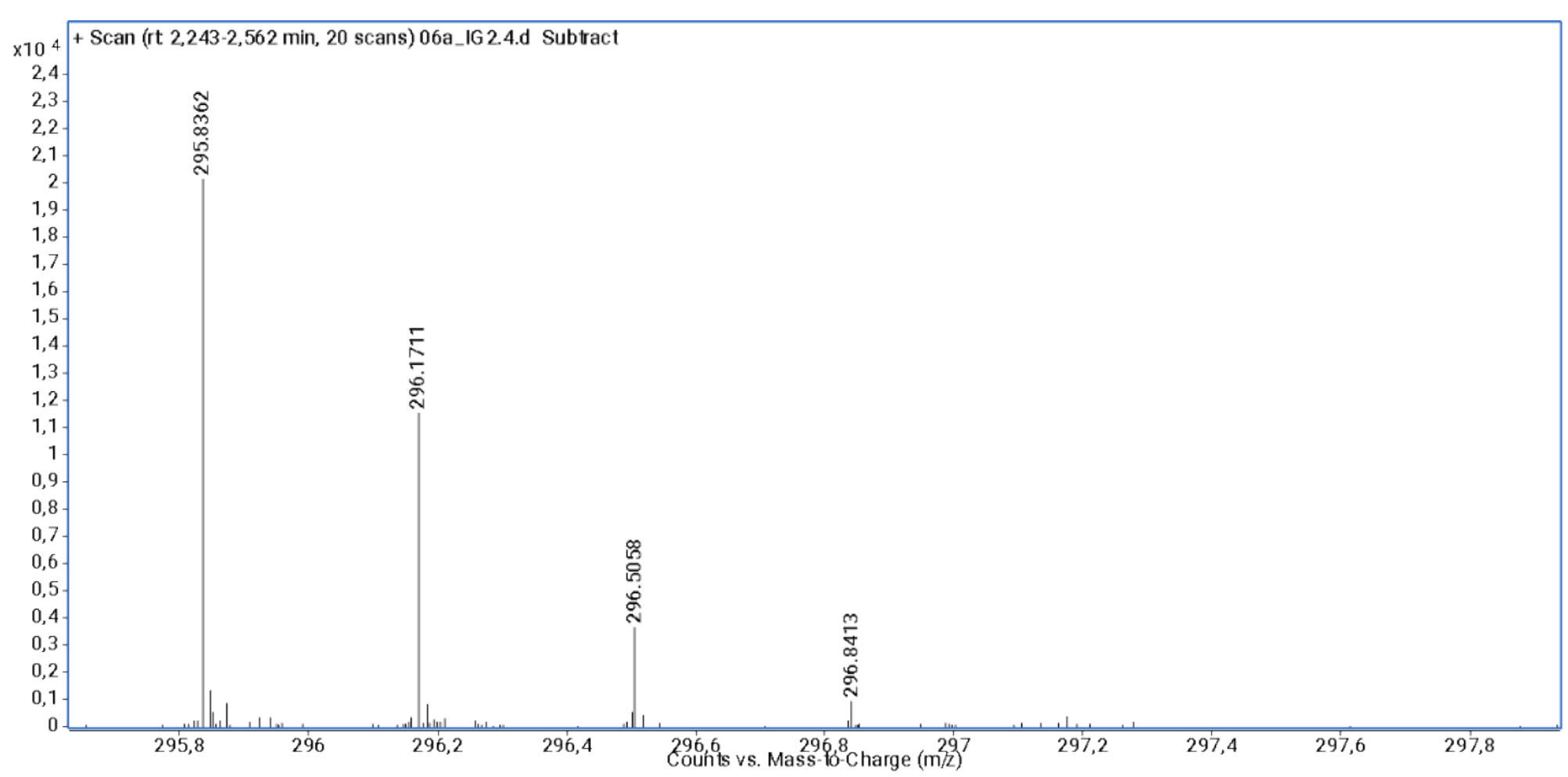

Fig. S13 Enlarged spectra from **Fig. S11** in the area m/z 295.8–297.8.

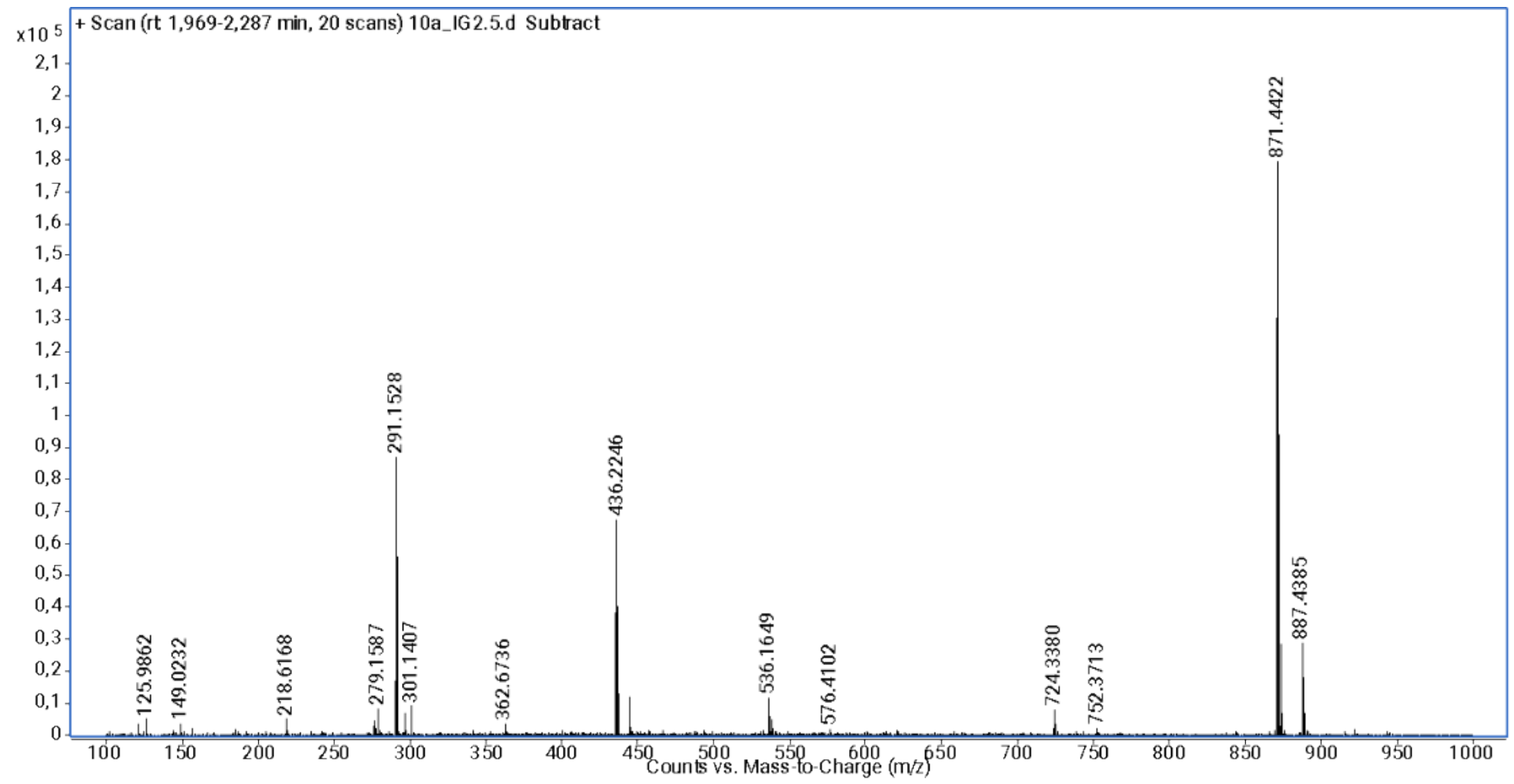

Fig. S14 Mass spectrum of ***trans*-A_2B_2** in the m/z range 100 – 1000.

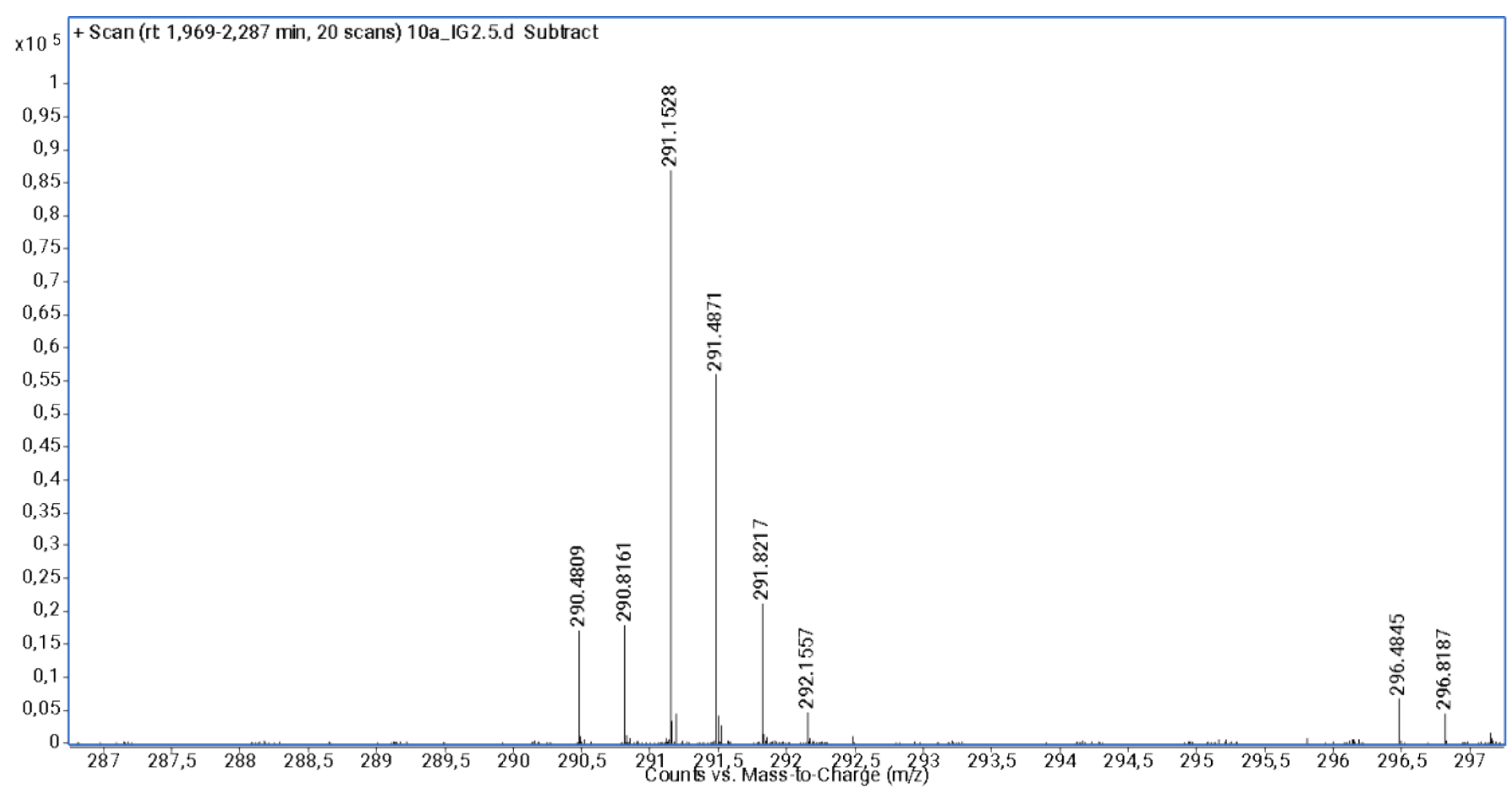

Fig. S15 Enlarged spectra from **Fig. S14** in the area m/z 287 – 297.

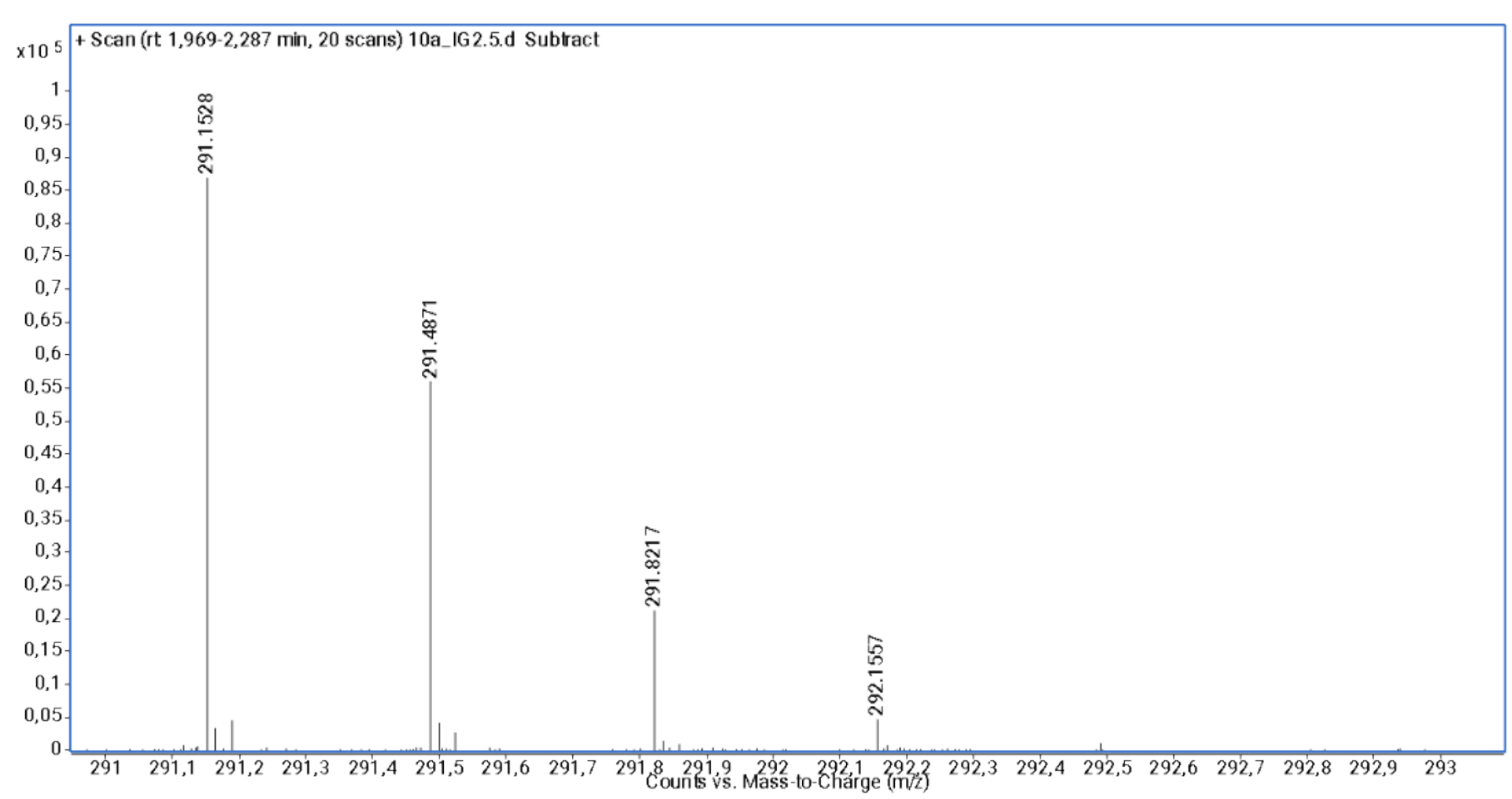

Fig. S16 Enlarged spectra from **Fig. S14** in the area m/z 291 – 293.

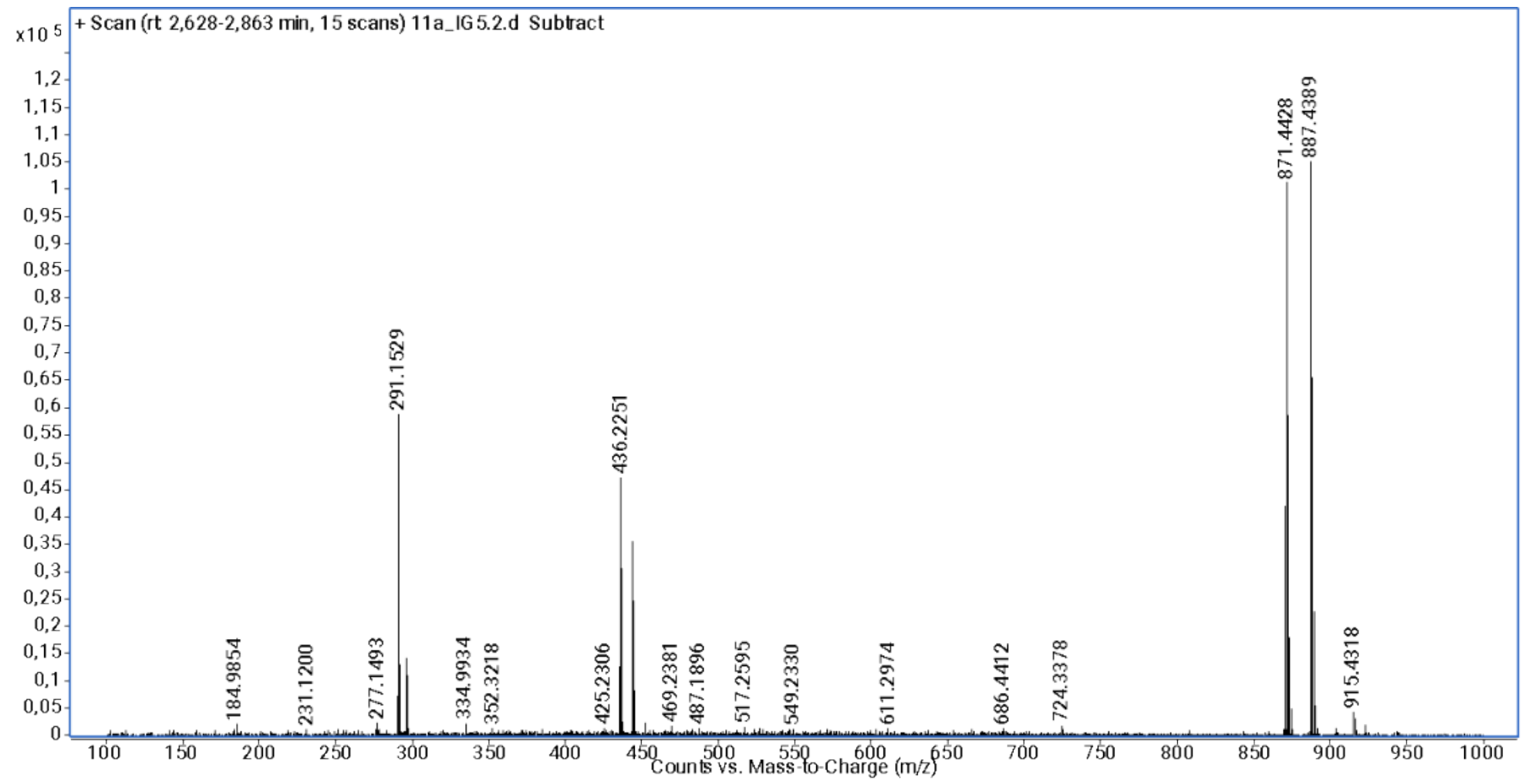

Fig. S17 Mass spectrum of ***cis*-A_2B_2** in the m/z range 100 – 1000.

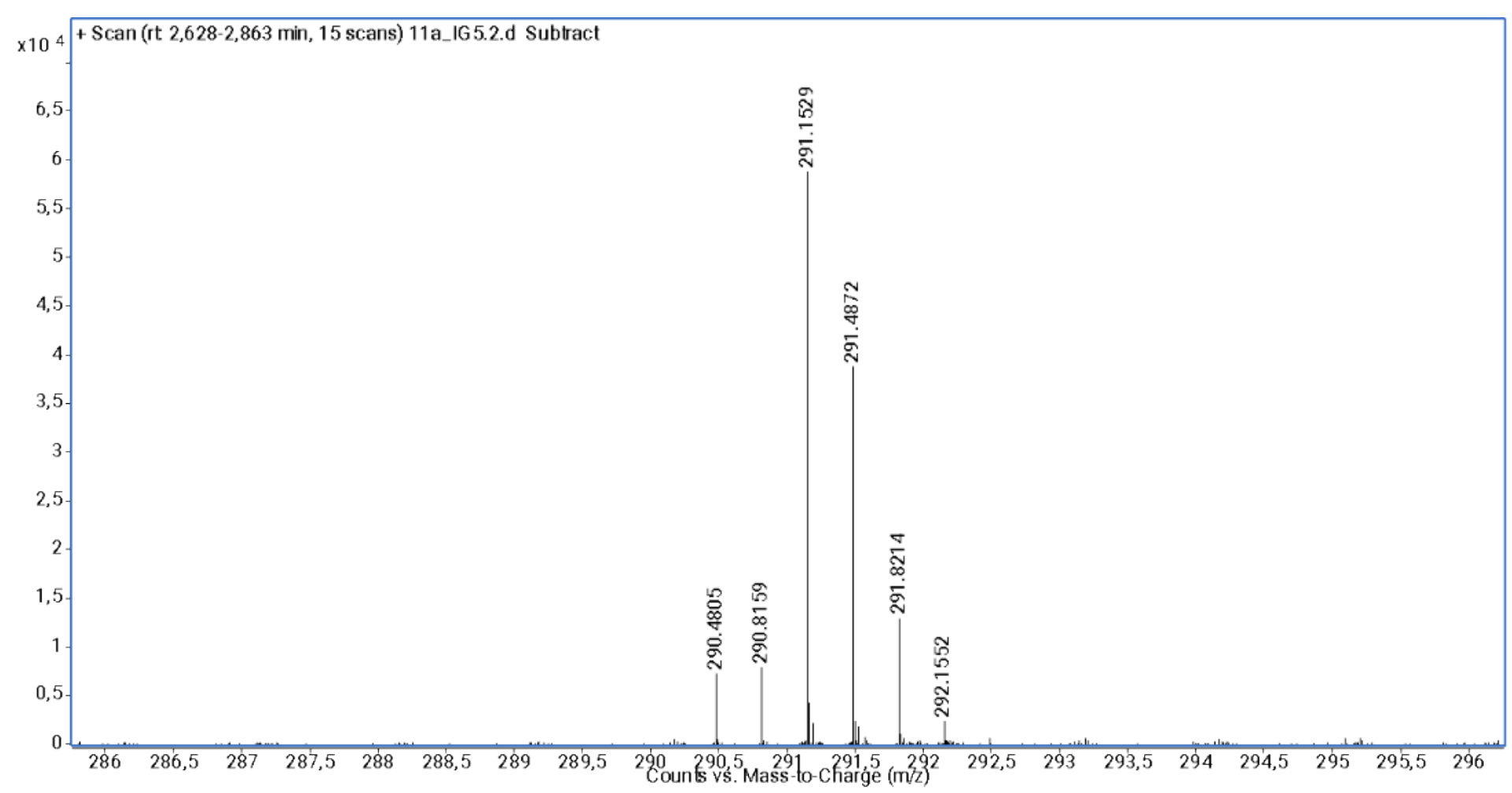

Fig. S18 Enlarged spectra from **Fig. S17** in the area m/z 286 – 296.

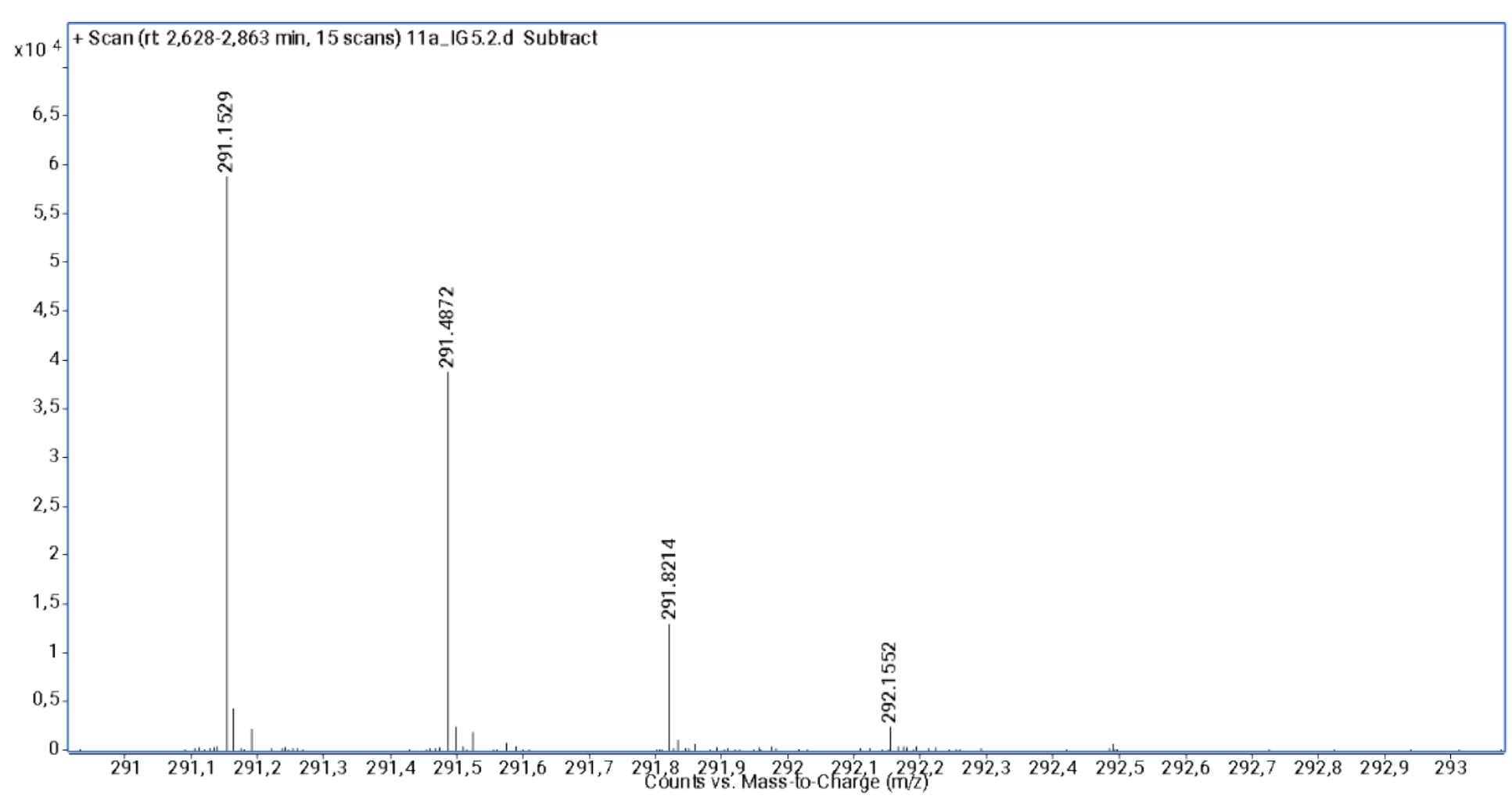

Fig. S19 Enlarged spectra from **Fig. S17** in the area m/z 291 – 293.

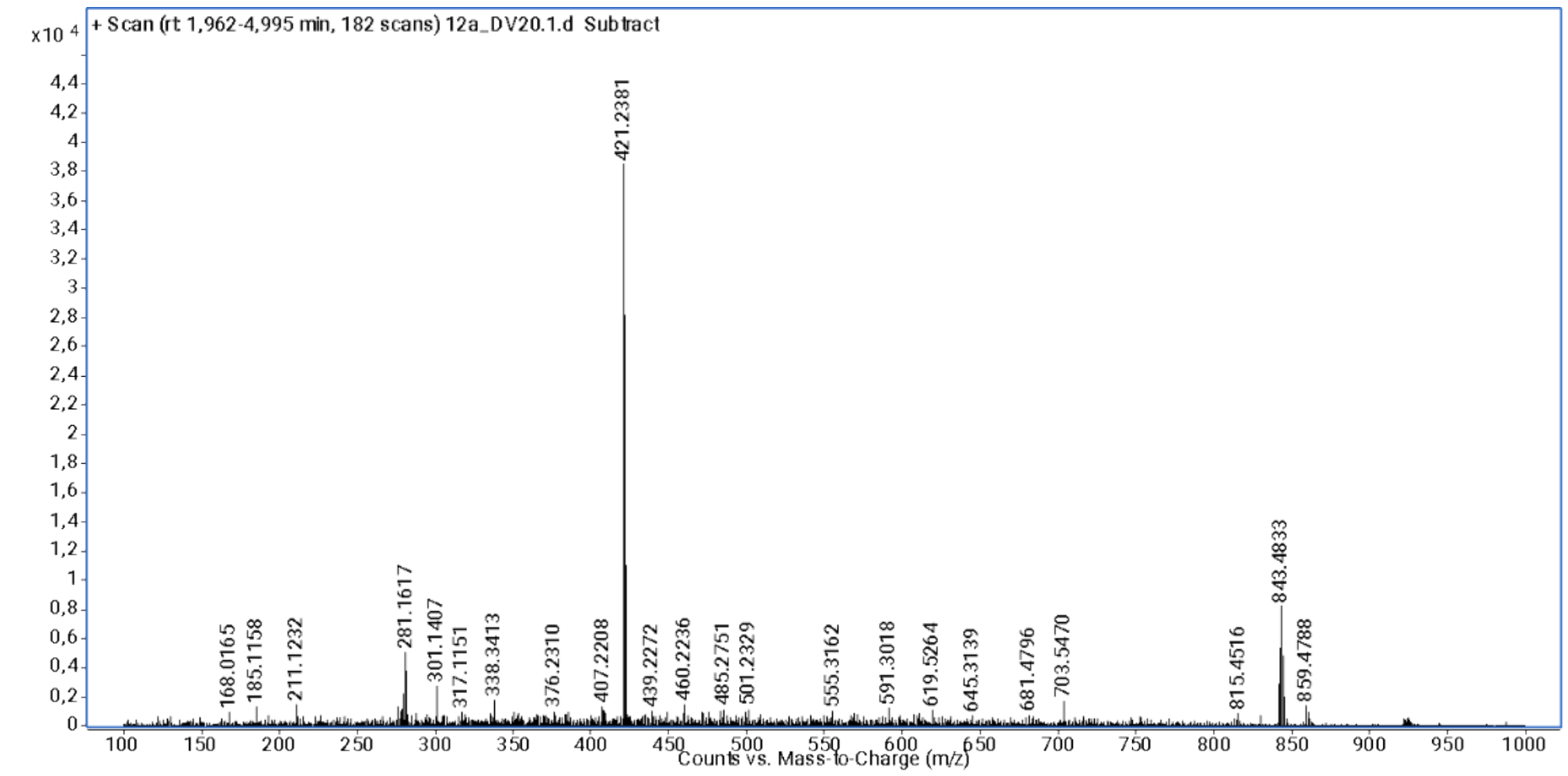

Fig. S20 Mass spectrum of NH_2**-AB**$_3$ in the m/z range 100 – 1000.

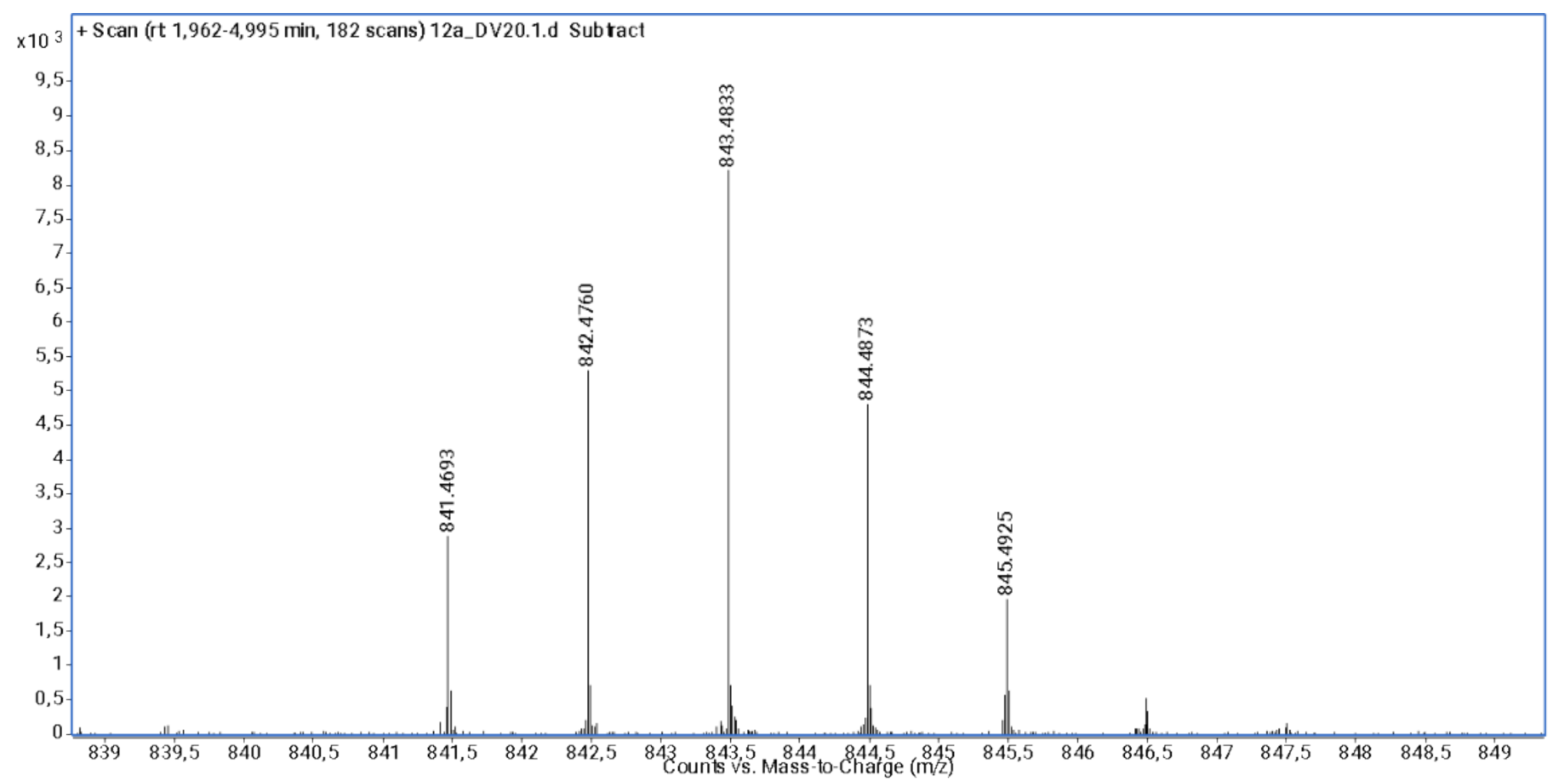

Fig. S21 Enlarged spectra from **Fig. S20** in the area m/z 839 – 849.

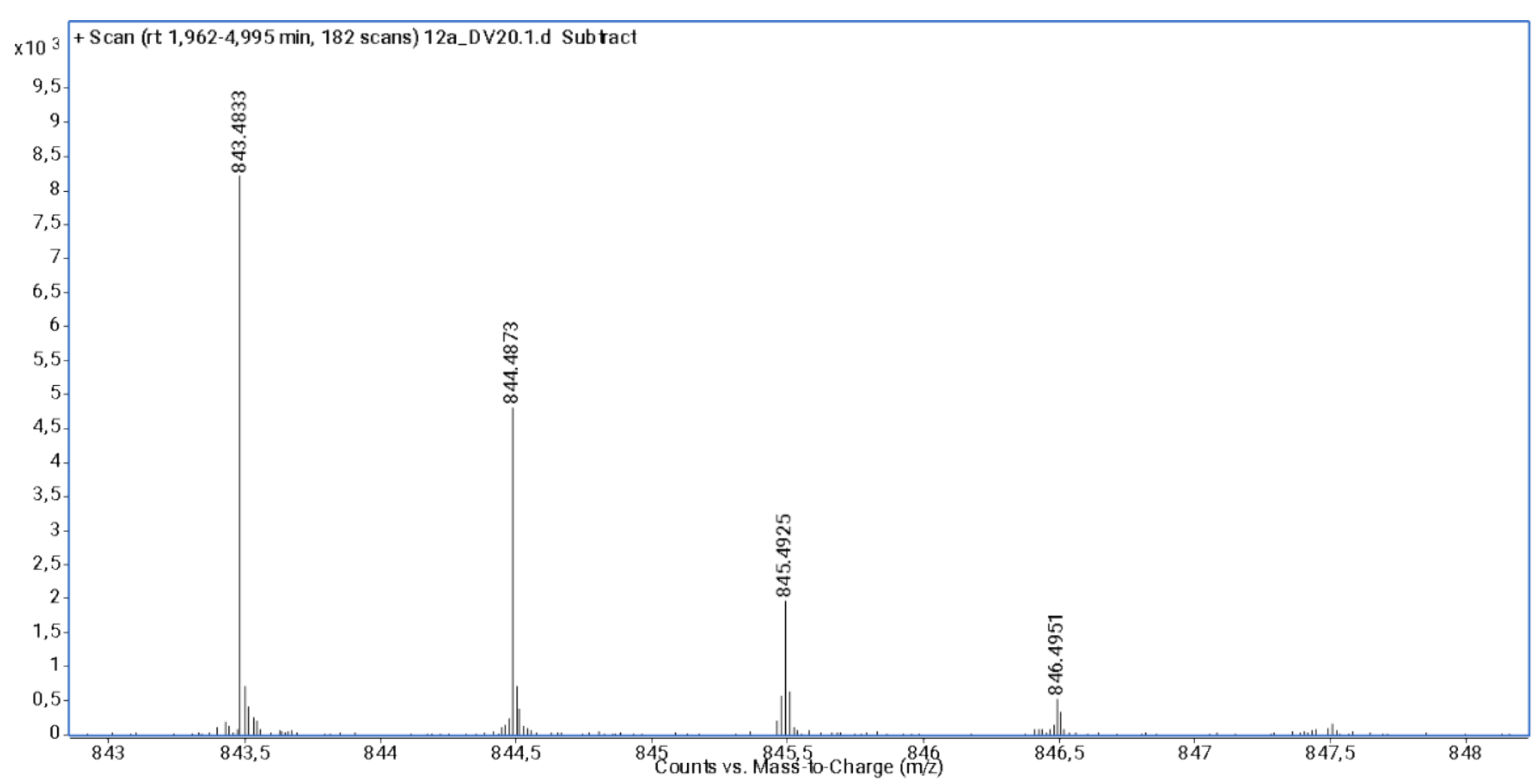

Fig. S22 Enlarged spectra from **Fig. S20** in the area m/z 843 – 848.

3. ^{1}H NMR spectra of acid titrations

(a)

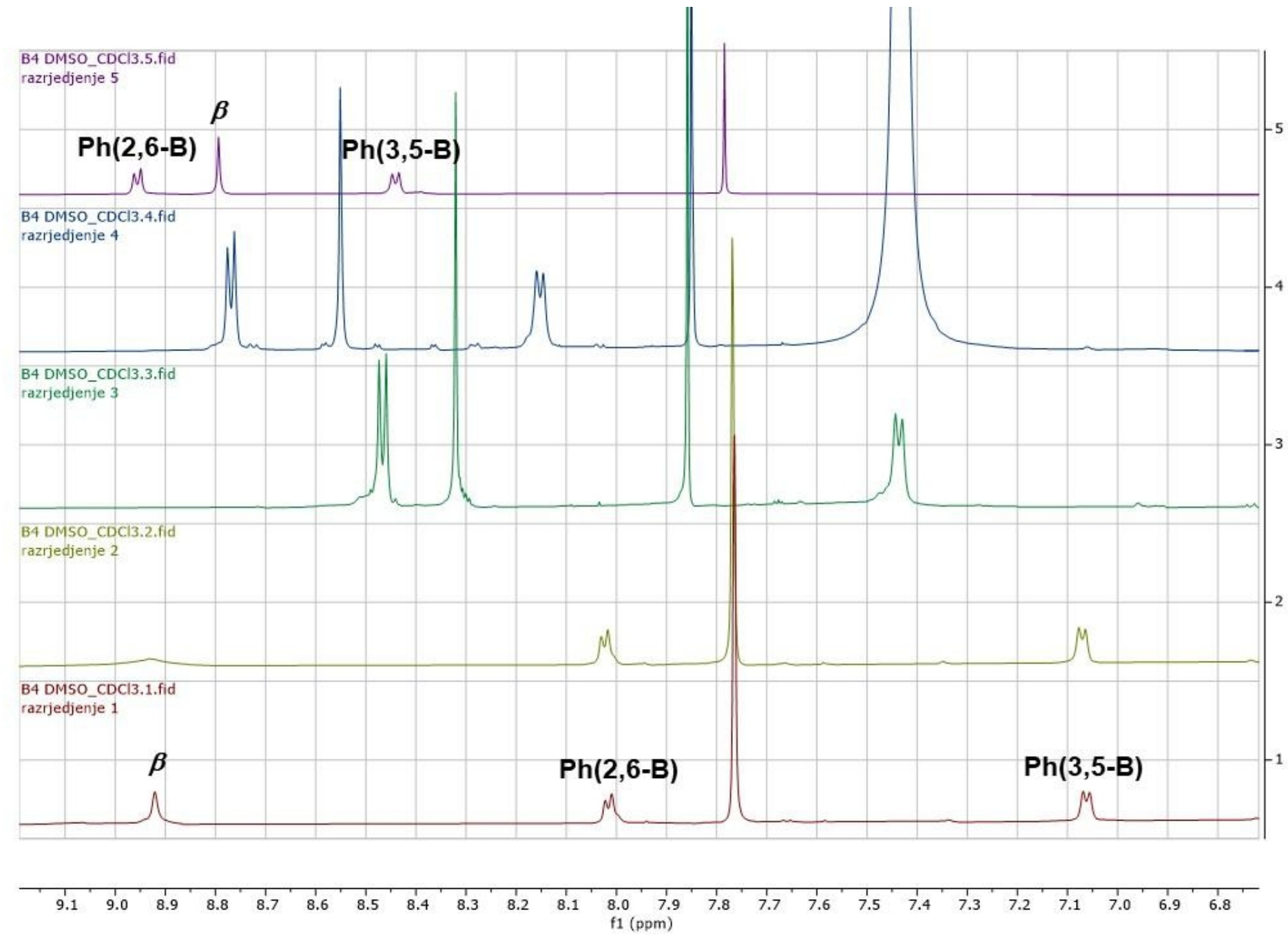

(b)

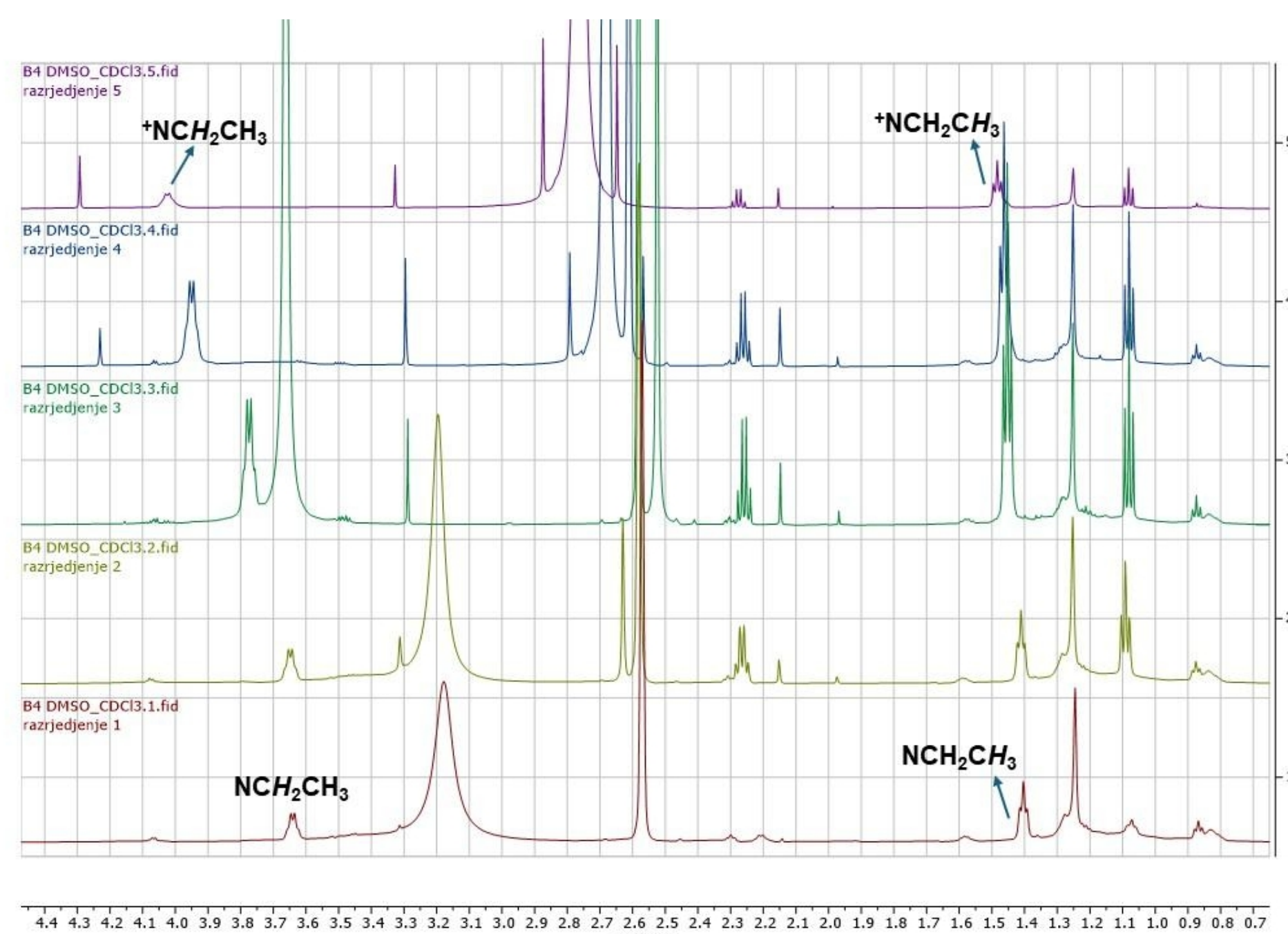

Fig. S23 ^{1}H NMR spectra in (a) aromatic and (b) aliphatic region for **B_4** (2 mM) recorded in $CDCl_3$:DMSO-d_6 (2:1) after titrations with MSA. Starting volume of the porphyrin solution was 500 µL, and the volumes of MSA consecutively added were: 0 µL (1st row/bottom spectra), 2 µL (2nd row), 10 µL (3rd), 150 µL (4th) and 750 µL (5th).

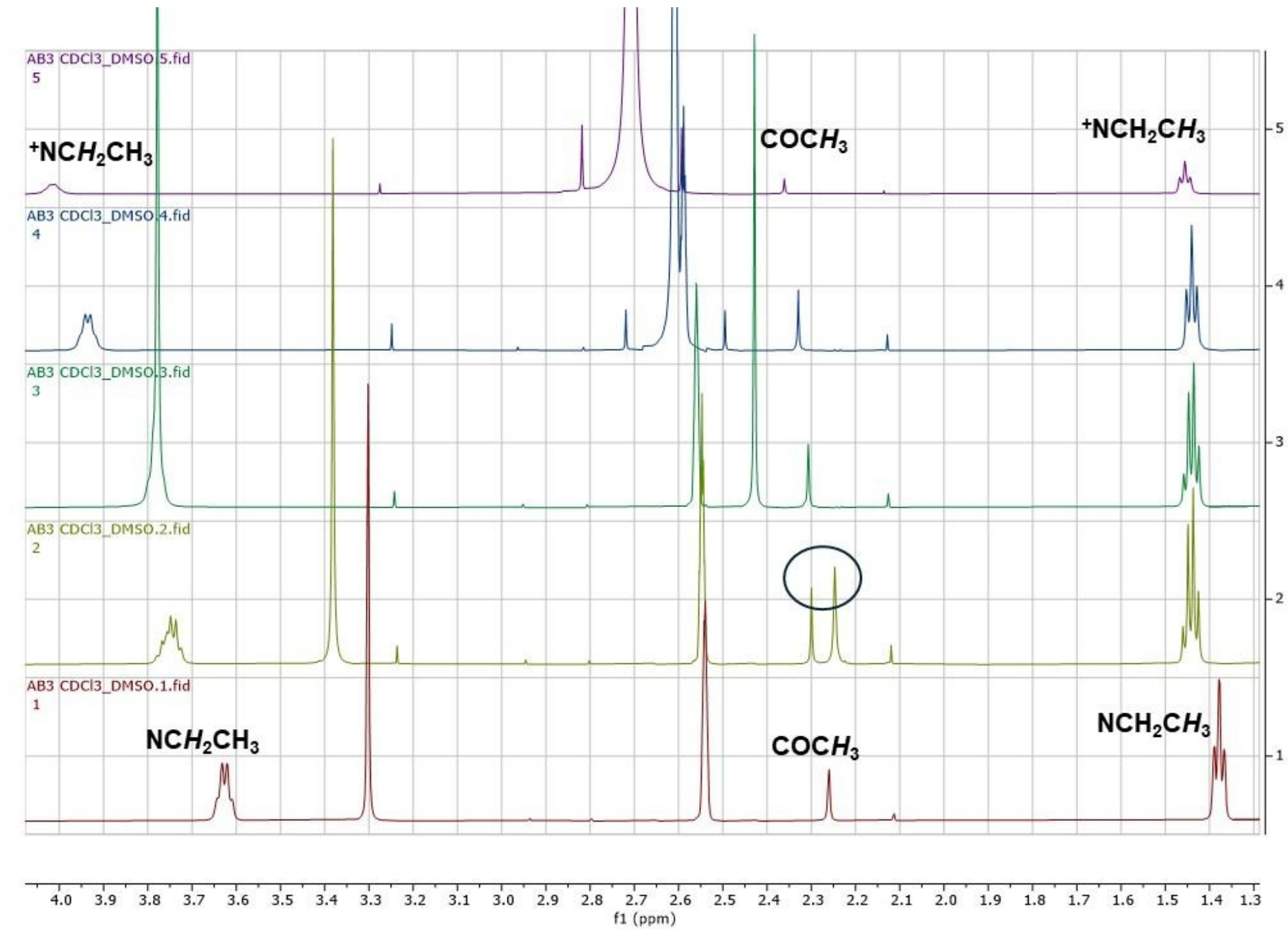

Fig. S24 ^{1}H NMR spectra (aliphatic region) for **AB_3** (6 mM) recorded in $CDCl_3$:DMSO-d_6 (1:1) after titrations with MSA. Starting volume of the porphyrin solution was 500 µL, and the volumes of MSA consecutively added were: 0 µL (1st row), 5 µL (2nd row), 10 µL (3rd), 150 µL (4th) and 750 µL (5th).

(a)

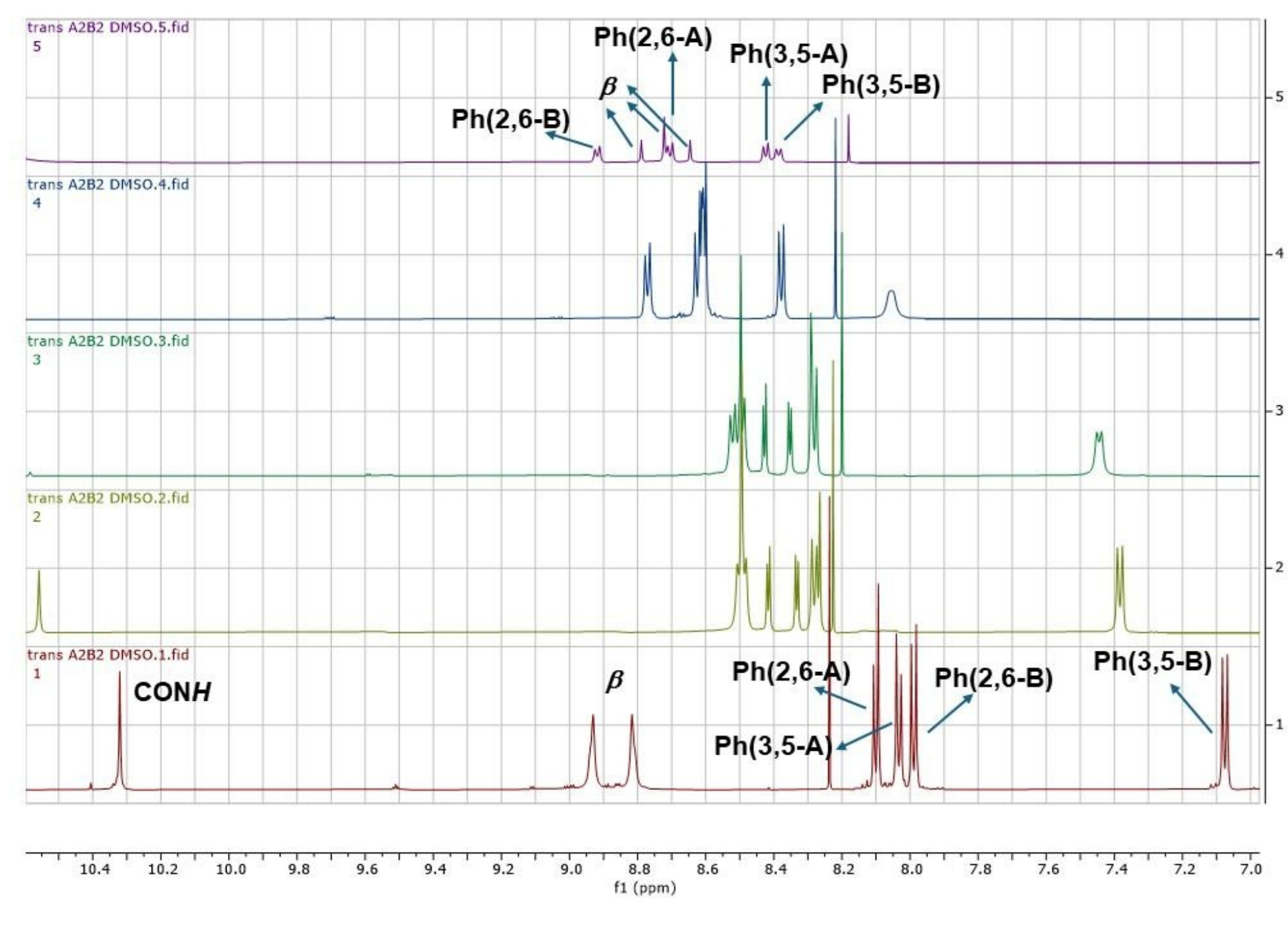

(b)

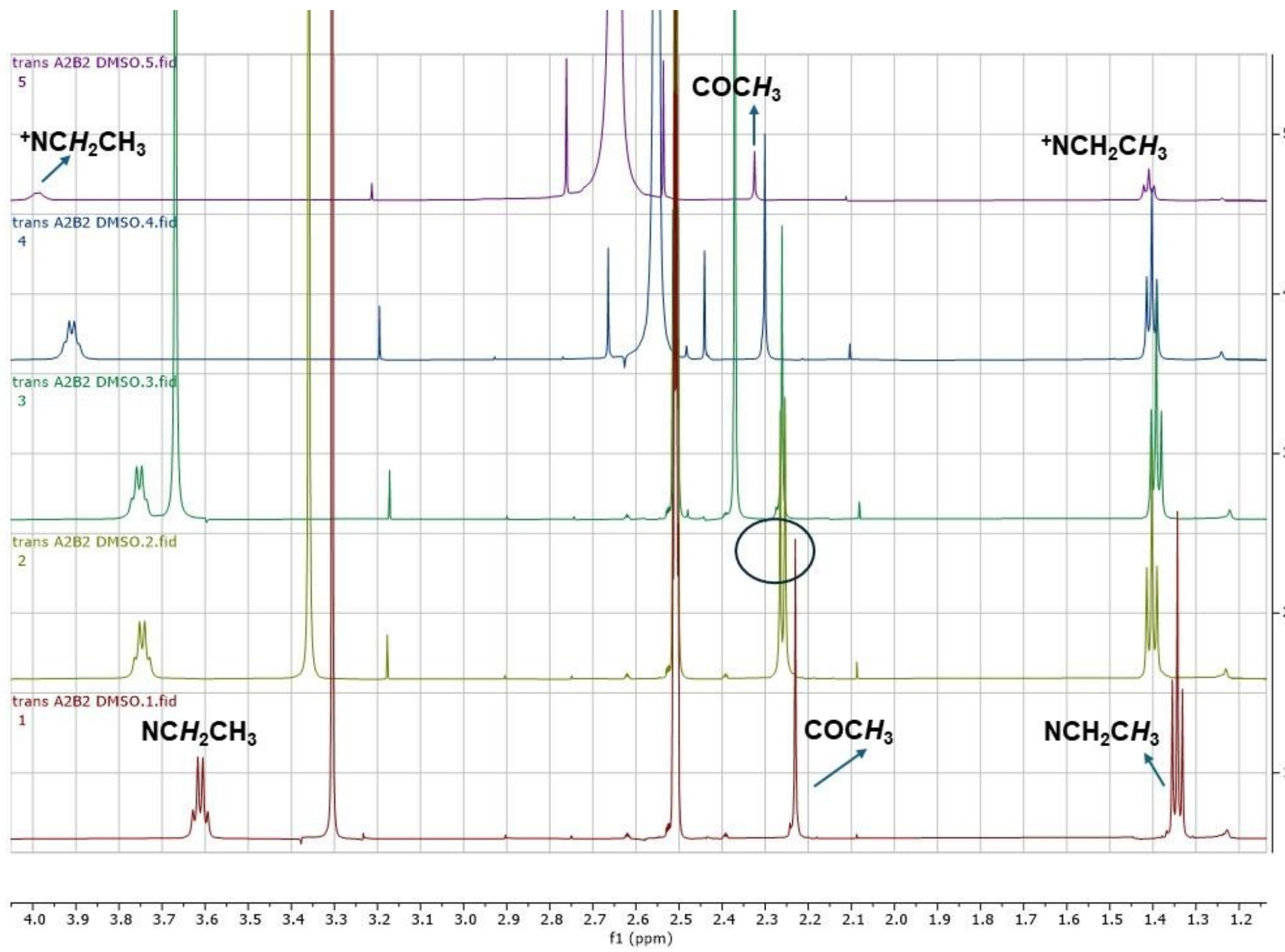

Fig. S25 ^{1}H NMR spectra in (a) aromatic and (b) aliphatic region for ***trans*-A$_2$B$_2$** (6 mM) recorded in DMSO-d$_6$ after titrations with MSA. Starting volume of the porphyrin solution was 500 µL, and the volumes of MSA consecutively added were: 0 µL (1st row), 5 µL (2nd row), 10 µL (3rd), 150 µL (4th) and 750 µL (5th).

(a)

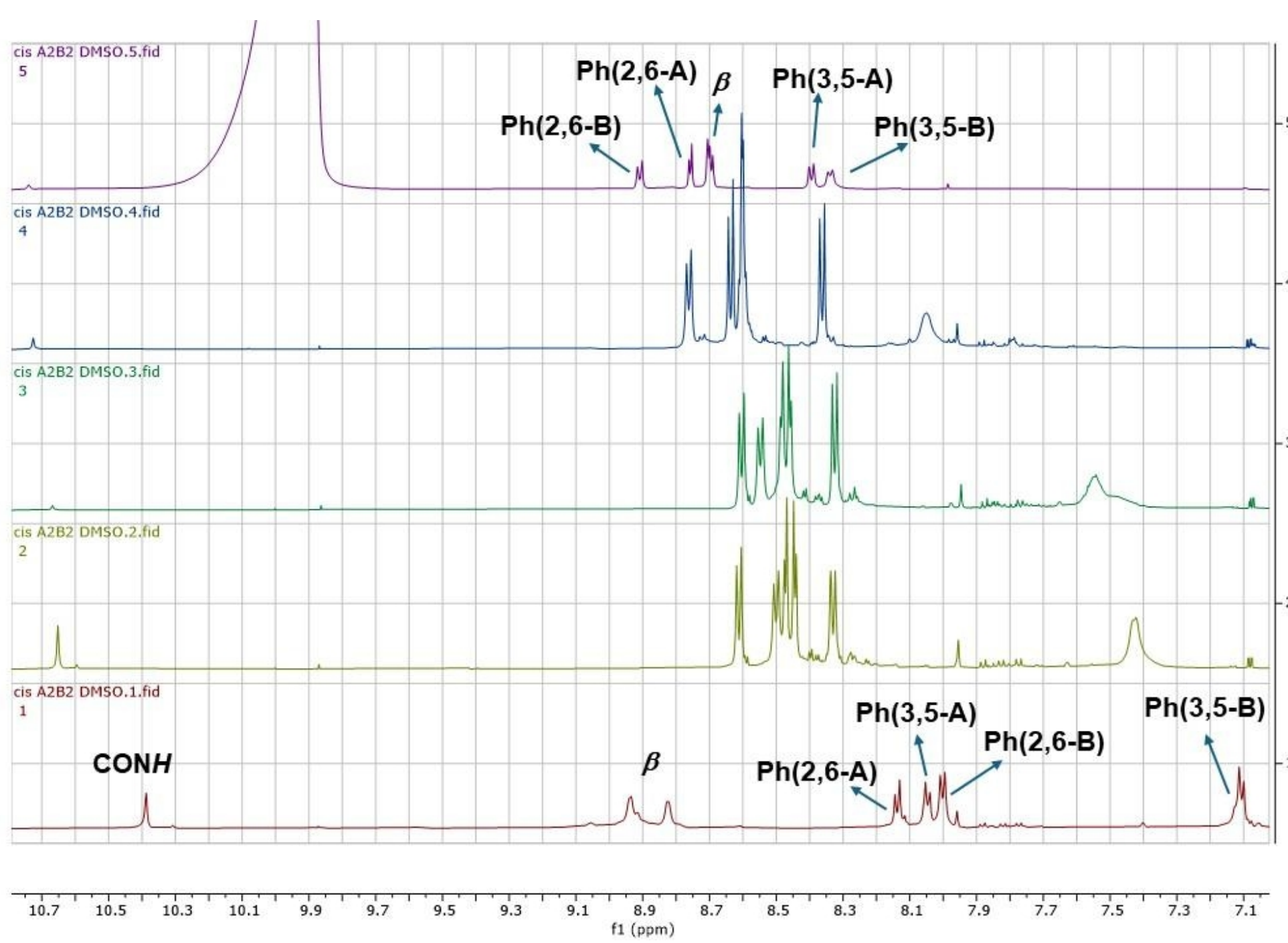

(b)

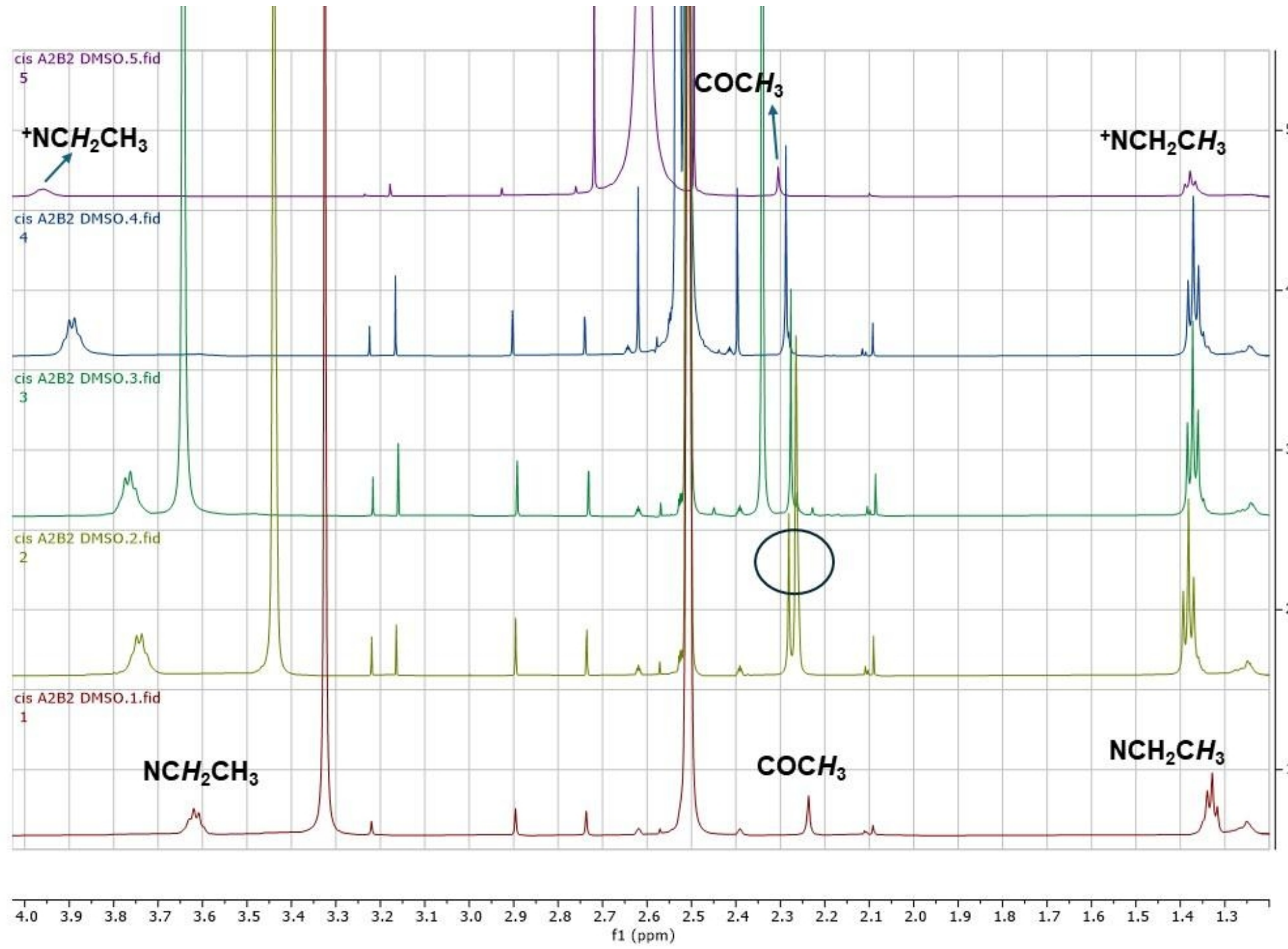

Fig. S26 ^{1}H NMR spectra (a) aromatic and (b) aliphatic region for ***cis*-A$_2$B$_2$** (6 mM) recorded in DMSO-d$_6$ after titrations with MSA. Starting volume of the porphyrin solution was 500 µL, and the volumes of MSA consecutively added were: 0 µL (1st row), 5 µL (2nd row), 10 µL (3rd), 150 µL (4th) and 750 µL (5th).

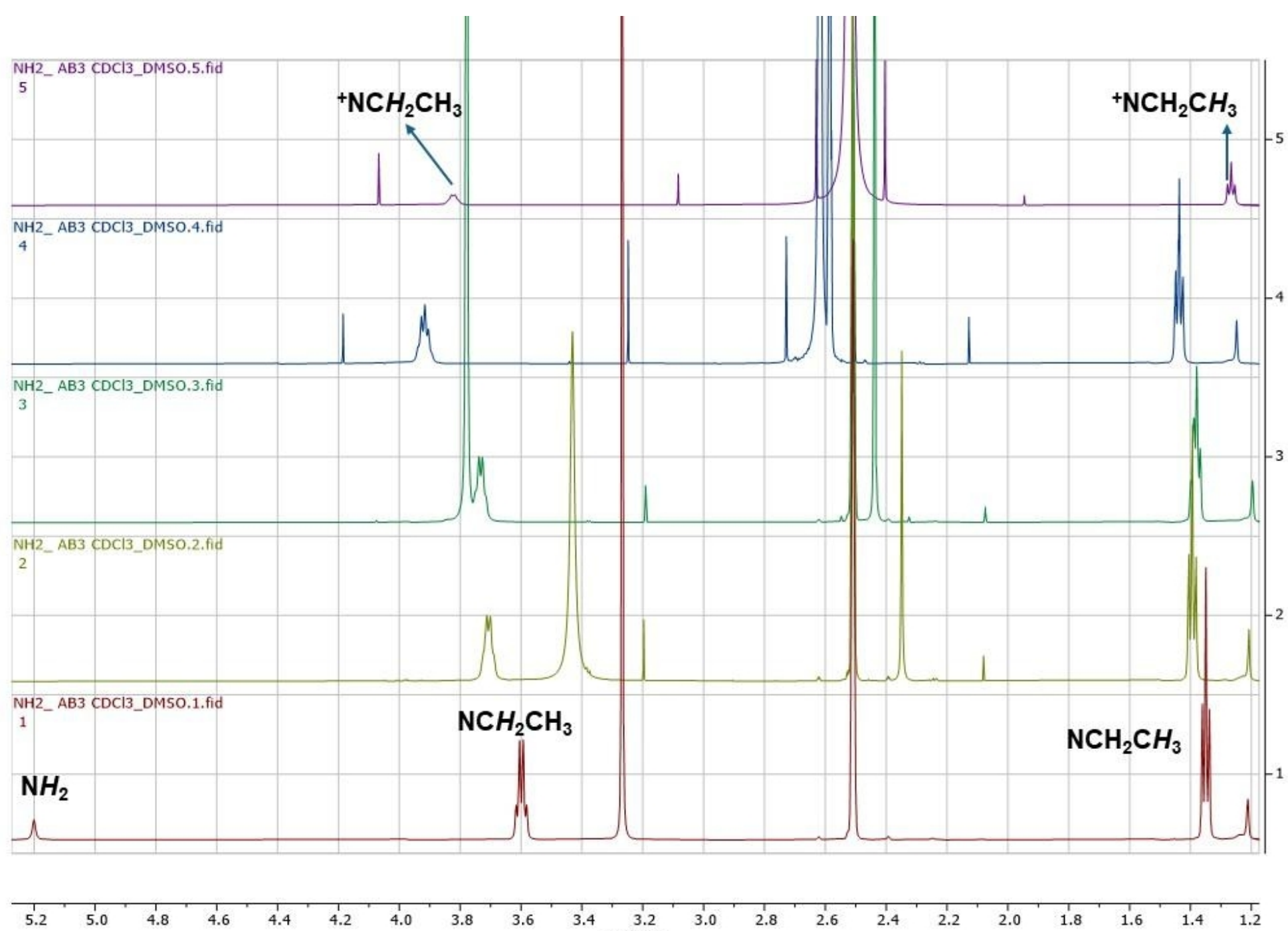

Fig. S27 ^{1}H NMR spectra (aliphatic region) for **NH_2-AB_3** (6 mM) recorded in $CDCl_3$:DMSO-d_6 (1:1) after titrations with MSA. Starting volume of the porphyrin solution was 500 µL, and the volumes of MSA consecutively added were: 0 µL (1st row), 5 µL (2nd row), 10 µL (3rd), 150 µL (4th) and 750 µL (5th).

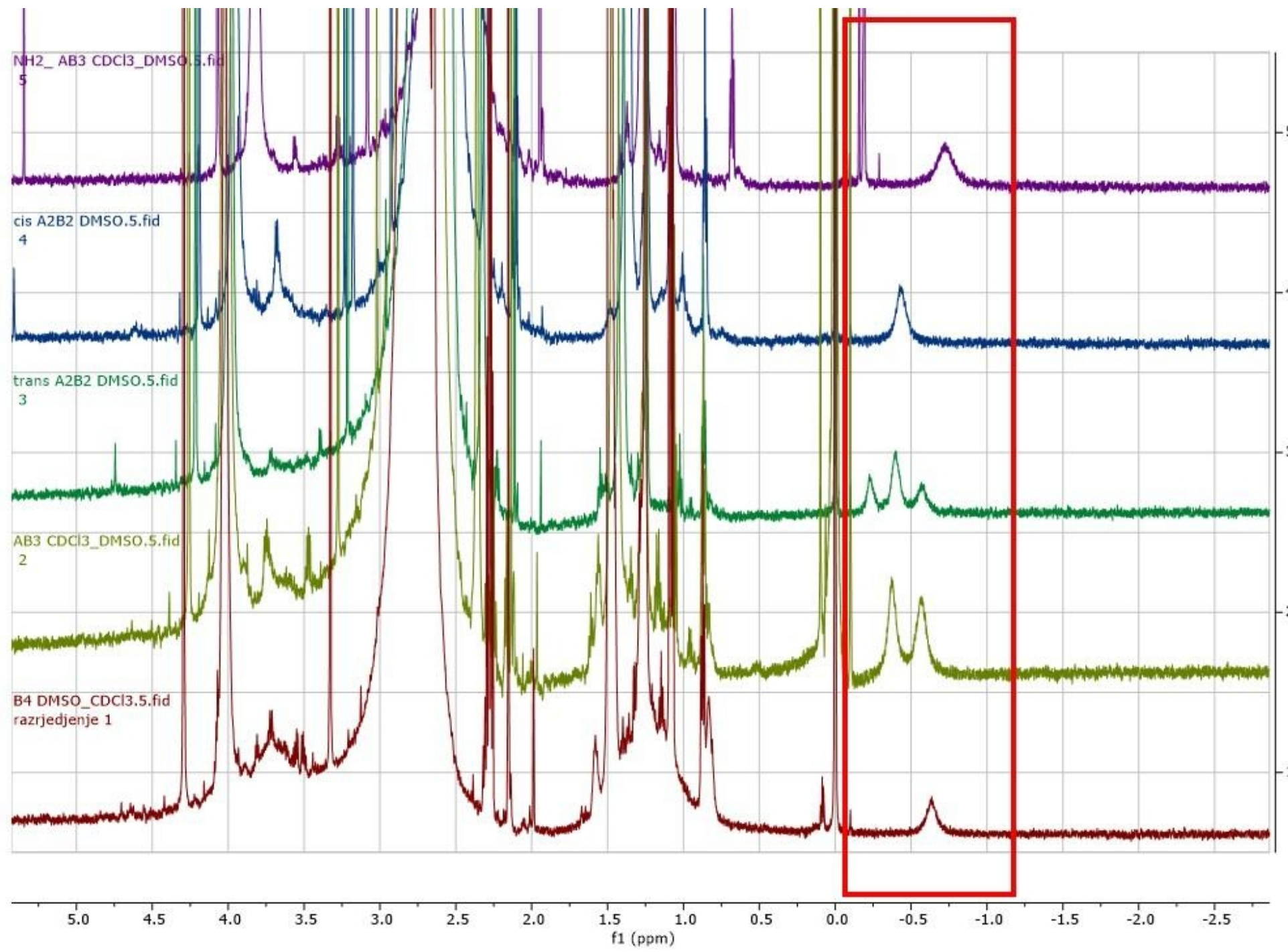

Fig. S28 Comparison of ^{1}H NMR spectra between porphyrins (from bottom to top) **B_4**, **AB_3**, ***trans*-A_2B_2**, ***cis*-A_2B_2** and **NH_2-AB_3**, after the last addition of MSA and complete porphyrin protonation (in DMSO-d_6, or $CDCl_3$:DMSO-d_6; volumes of MSA added same as described in Fig. S23−S27) showing signals for inner (pyrrole) protons (red square).

4. Hyperporphyrin resonance forms

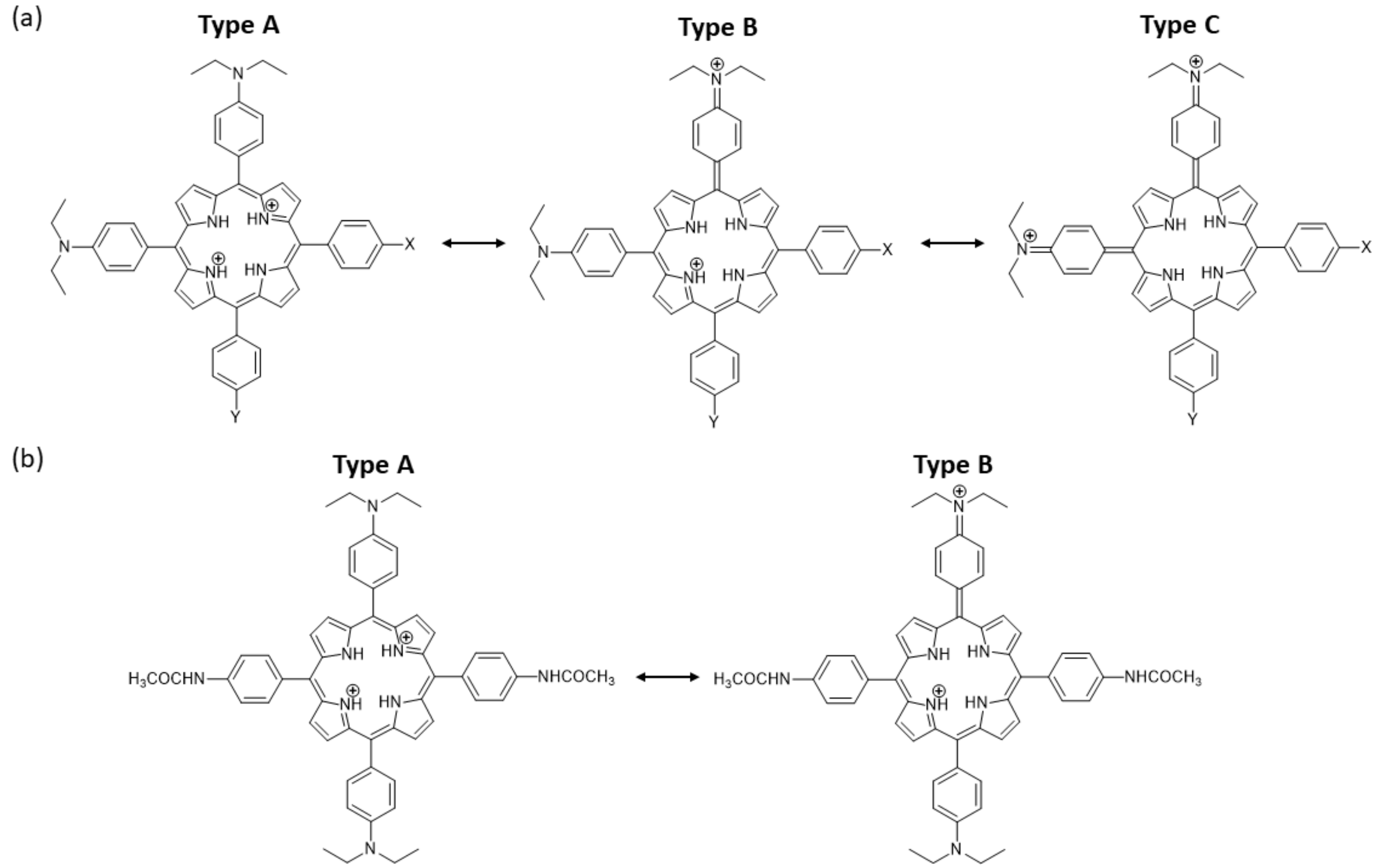

Scheme S1 Hyperporphyrin resonance forms for diprotonated porphyrins: (a) $\mathbf{B_4H_2^{2+}}$ (X,Y = NEt_2), $\mathbf{AB_3H_2^{2+}}$ (X = NEt_2,Y = $NHCOCH_3$), ***cis*-$\mathbf{A_2B_2H_2^{2+}}$** (X,Y = $NHCOCH_3$), **amino $\mathbf{AB_3H_2^{2+}}$** (X = NEt_2, Y = NH_2), and (b) ***trans*-$\mathbf{A_2B_2}$**.

Table S1 Number of resonance forms for each hyperporphyrin type for diprotonated, triprotonated and tetraprotonated porphyrins formed/detected in this study (according to [1]).

hyper-porphyrin	$B_4H_2^{2+}$	$B_4H_4^{4+}$	$AB_3H_2^{2+}$	$AB_3H_4^{4+}$	*trans*-$A_2B_2H_2^{2+}$	*trans*-$A_2B_2H_3^{3+}$	*cis*-$A_2B_2H_2^{2+}$	*cis*-$A_2B_2H_3^{3+}$	NH_2-$AB_3H_2^{2+}$	NH_2-$AB_3H_4^{4+}$
**Type B**	4	2	3	2	2	1	2	1	4	2
**Type C**	4	1	2	1	0	0	1	0	4	1

5. UV-Vis spectra of acid titrations

(a)

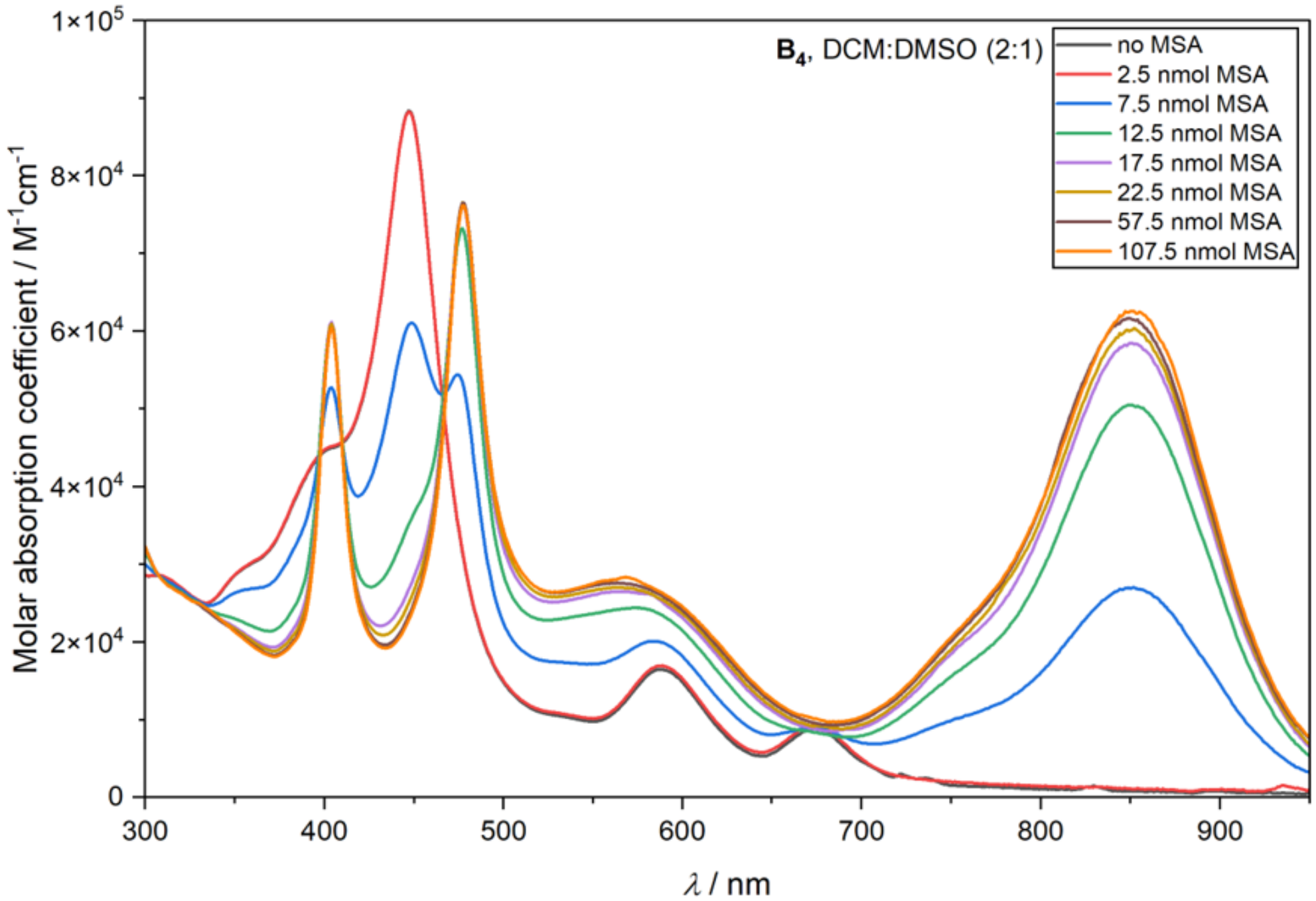

(b)

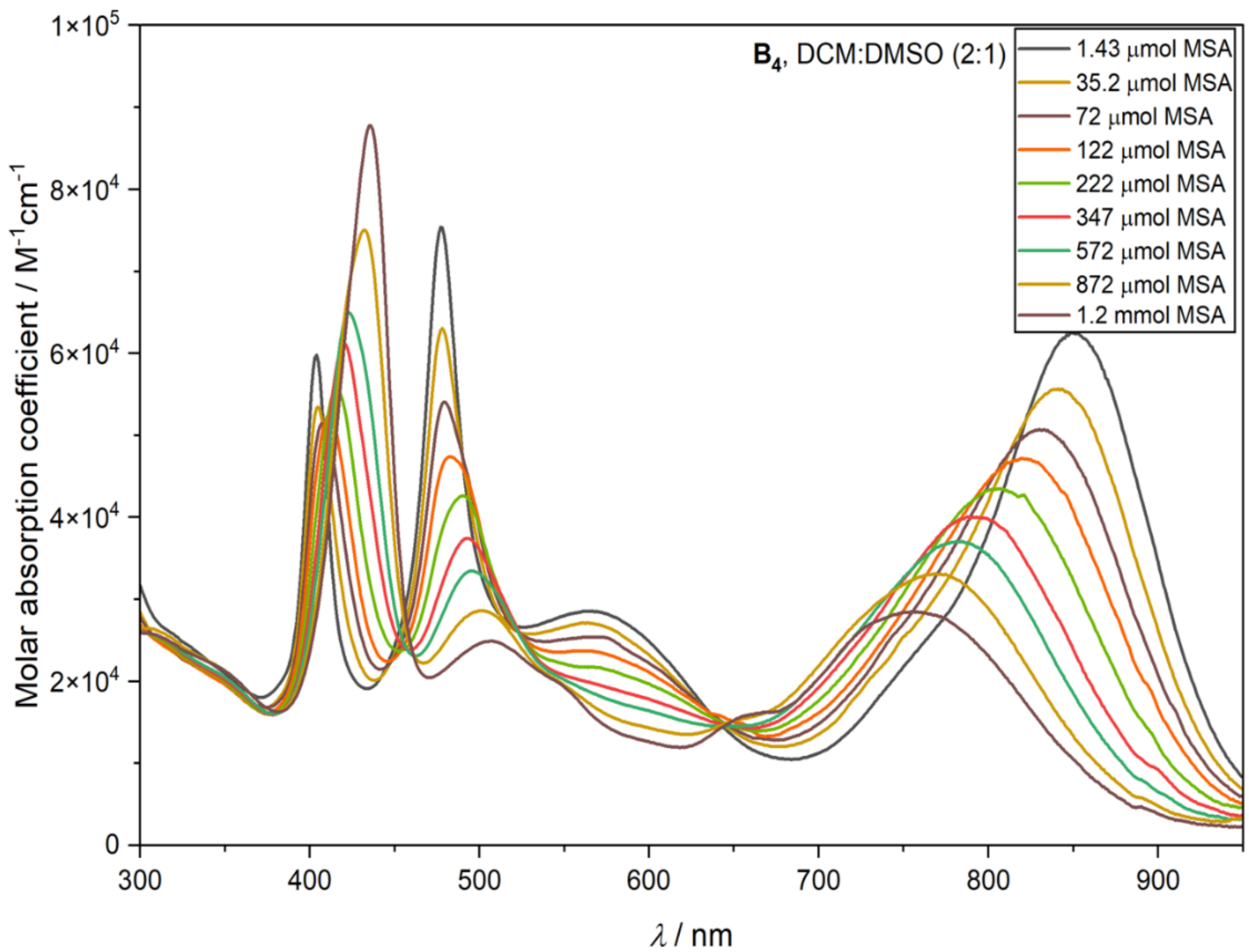

(c)

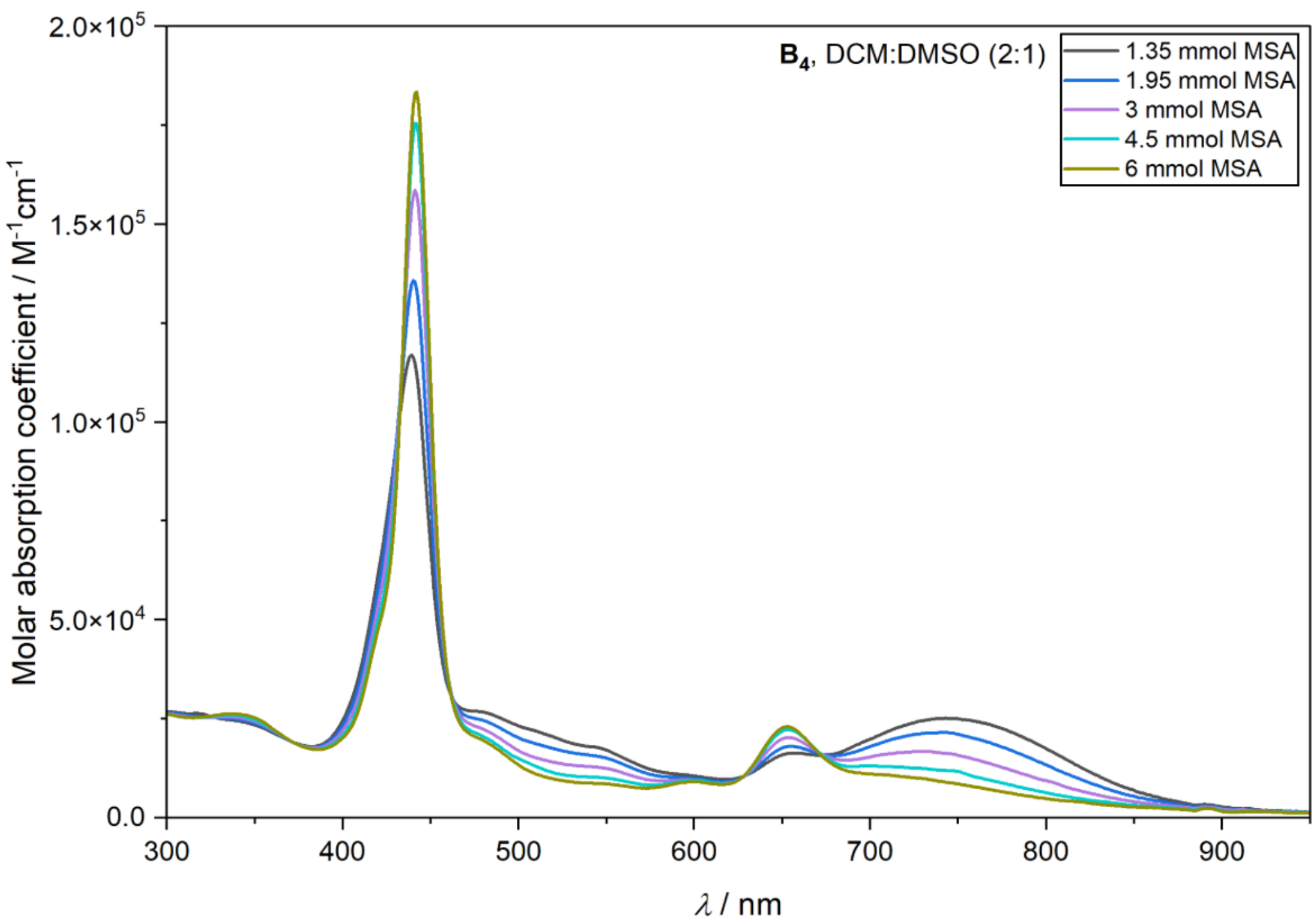

Fig. S29 Absorption spectra of **B_4** (8 µM) in DCM:DMSO (2:1) and apparent protonation stages upon addition of MSA: (a) **B_4** → **$B_4H_2^{2+}$**, 0−107.5 nmol MSA; (b) **$B_4H_2^{2+}$** → **$B_4H_4^{4+}$**, 1.43 µmol−1.22 mmol MSA; (c) **$B_4H_4^{4+}$** → **$B_4H_6^{6+}$**, 1.35−6 mmol MSA.

(a)

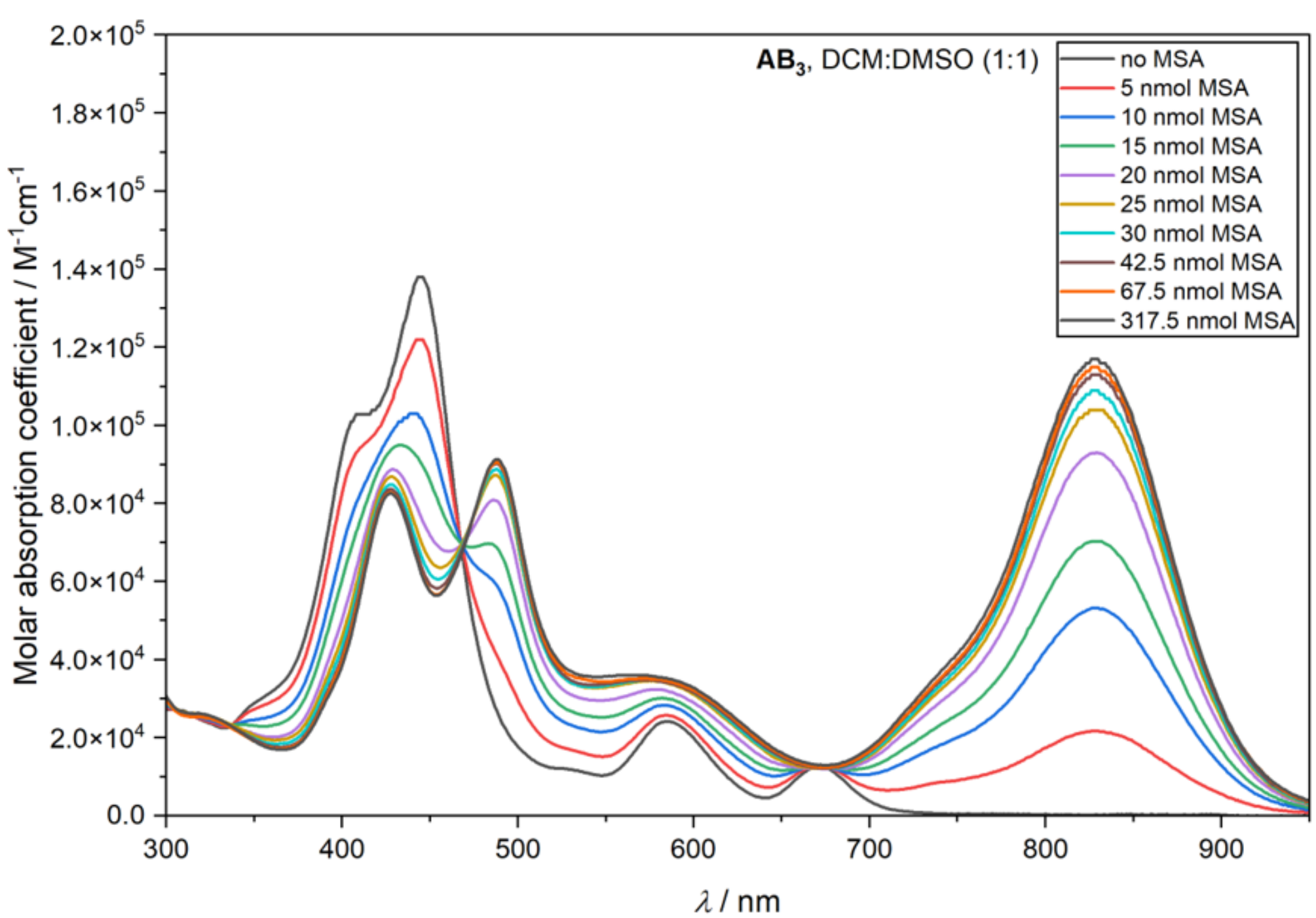

(b)

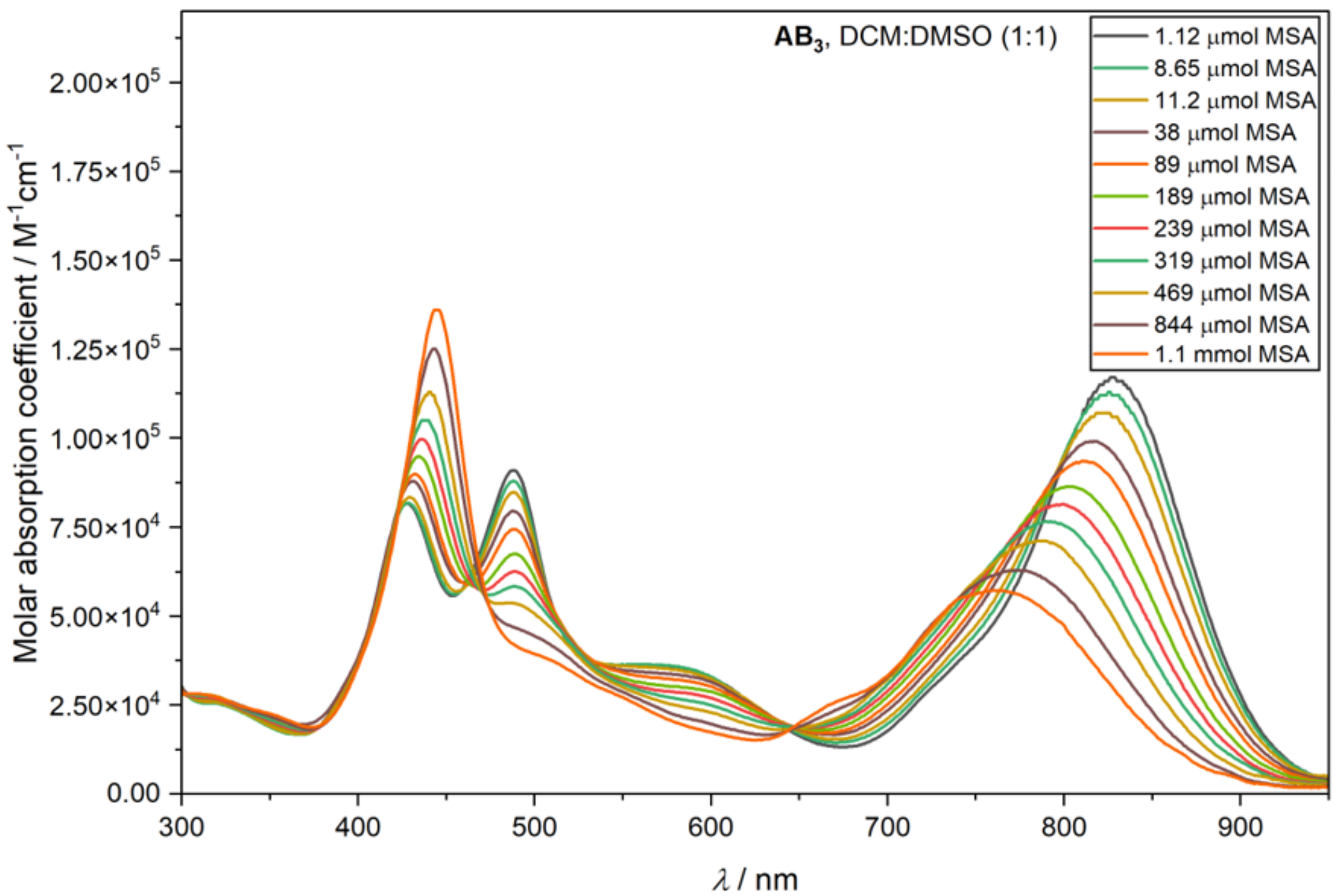

(c)

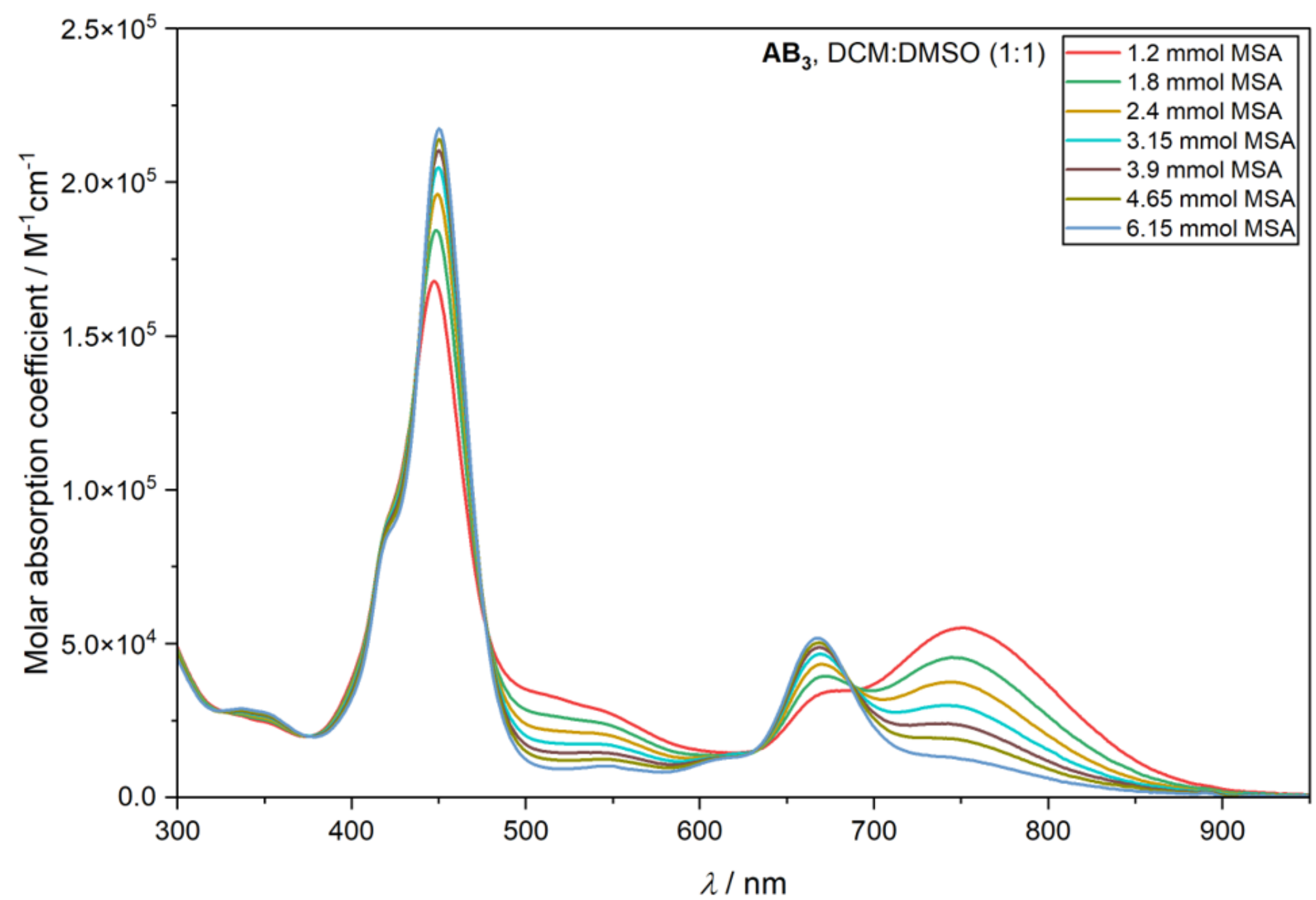

Fig. S30 Absorption spectra of $\mathbf{AB_3}$ (6 μM) in DCM:DMSO (1:1) and apparent protonation stages upon addition of MSA: (a) $\mathbf{AB_3} \rightarrow \mathbf{AB_3H_2^{2+}}$, 0−317.5 nmol MSA; (b) $\mathbf{AB_3H_2^{2+}} \rightarrow \mathbf{AB_3H_4^{4+}}$, 1.12 μmol−1.1 mmol MSA; (c) $\mathbf{AB_3H_4^{4+}} \rightarrow \mathbf{AB_3H_5^{5+}}$, 1.2−6.15 mmol MSA.

(a)

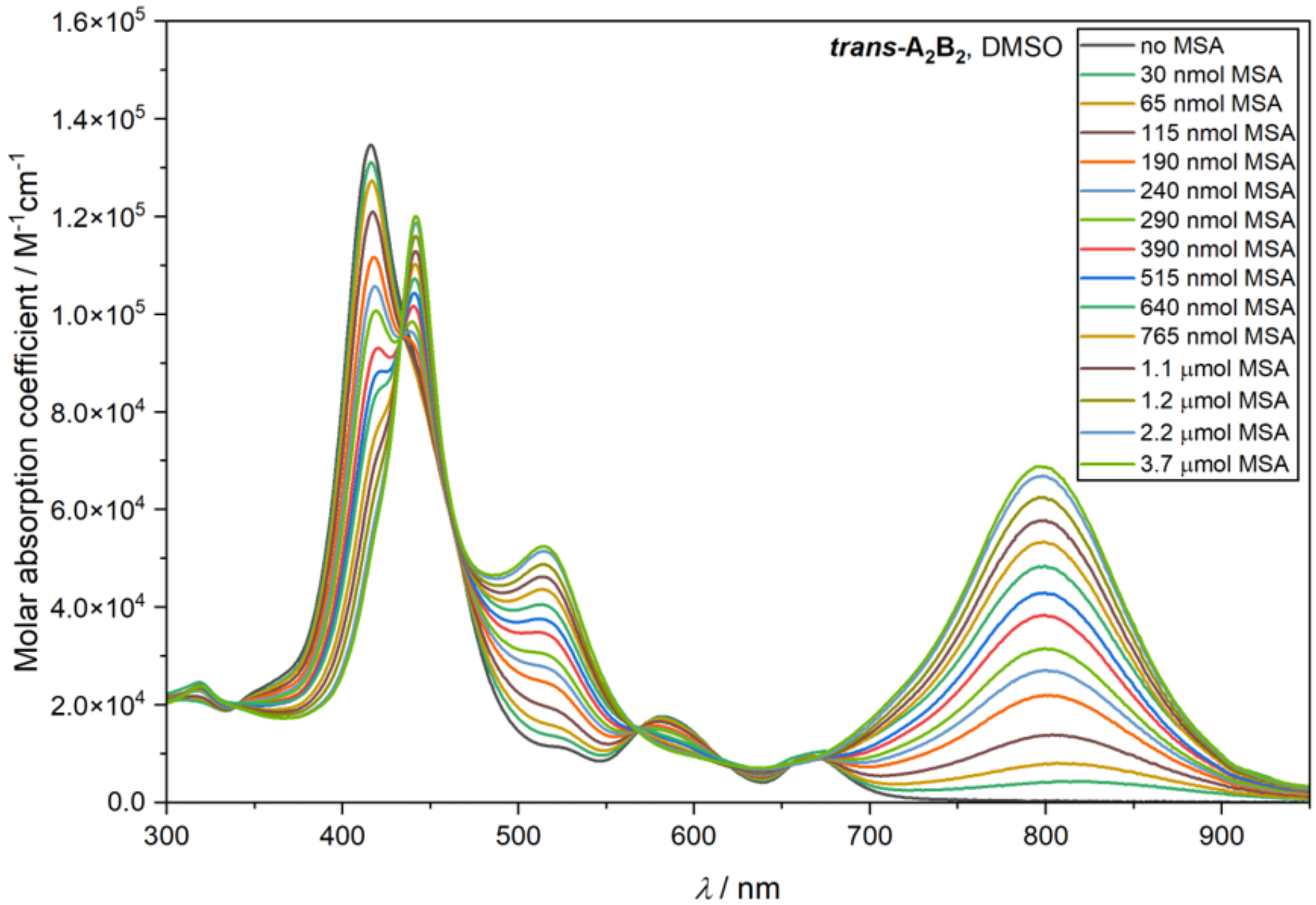

***trans*-A$_2$B$_2$**, DMSO
no MSA
30 nmol MSA
65 nmol MSA
115 nmol MSA
190 nmol MSA
240 nmol MSA
290 nmol MSA
390 nmol MSA
515 nmol MSA
640 nmol MSA
765 nmol MSA
1.1 μmol MSA
1.2 μmol MSA
2.2 μmol MSA
3.7 μmol MSA
Molar absorption coefficient / M^{-1}cm^{-1}
λ / nm

(b)

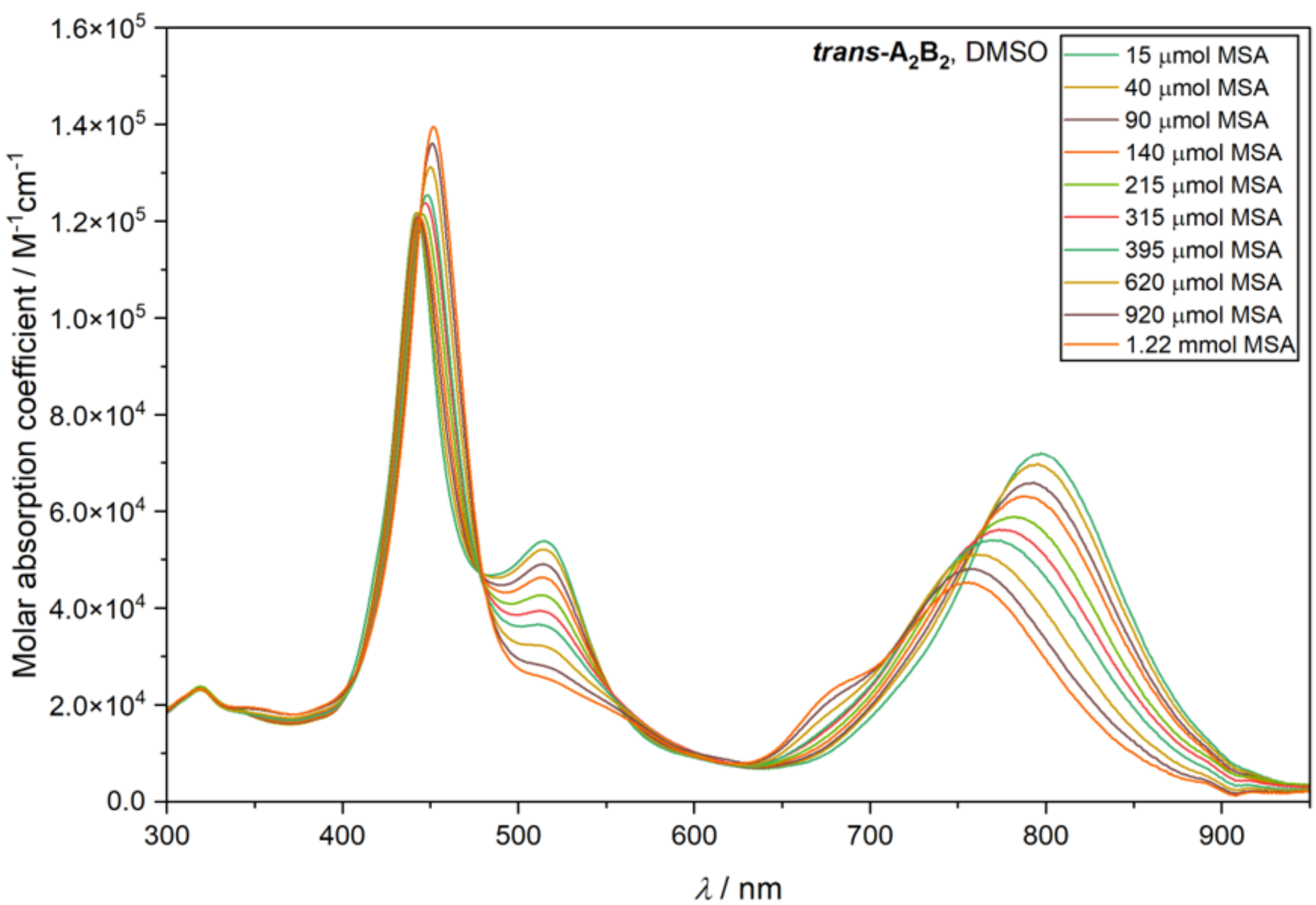

***trans*-A$_2$B$_2$**, DMSO
15 μmol MSA
40 μmol MSA
90 μmol MSA
140 μmol MSA
215 μmol MSA
315 μmol MSA
395 μmol MSA
620 μmol MSA
920 μmol MSA
1.22 mmol MSA
Molar absorption coefficient / M^{-1}cm^{-1}
λ / nm

(c)

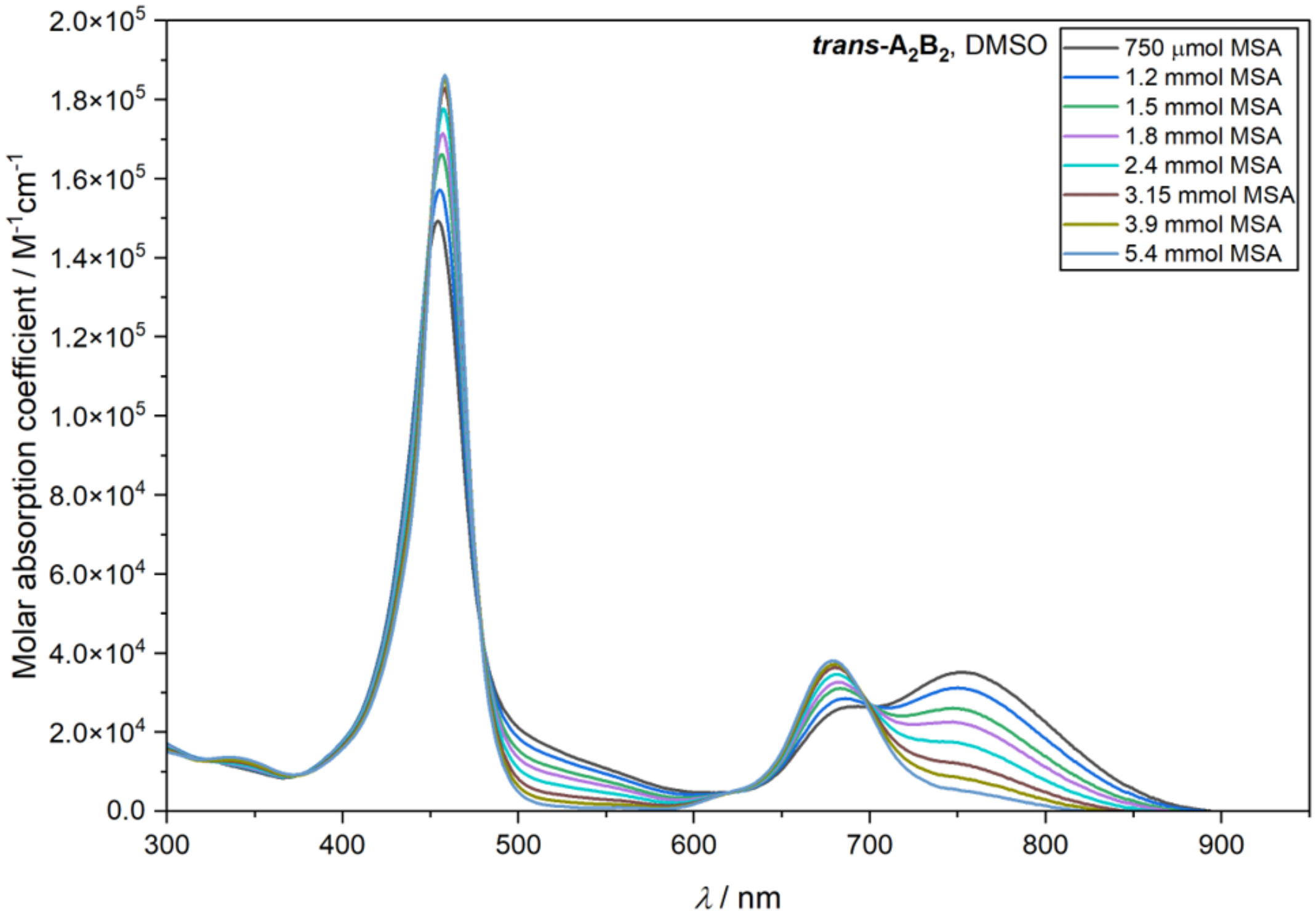

Fig. S31 Absorption spectra of ***trans*-A_2B_2** (6 µM) in DMSO and apparent protonation stages upon addition of MSA: (a) ***trans*-A_2B_2** → ***trans*-$A_2B_2H_2^{2+}$**, 0−3.7 µmol MSA; (b) ***trans*-$A_2B_2H_2^{2+}$** → ***trans*-$A_2B_2H_3^{3+}$**, 15 µmol−1.22 mmol MSA; (c) ***trans*-$A_2B_2H_3^{3+}$** → ***trans*-$A_2B_2H_4^{4+}$**, 0.75−5.4 mmol MSA.

(a)

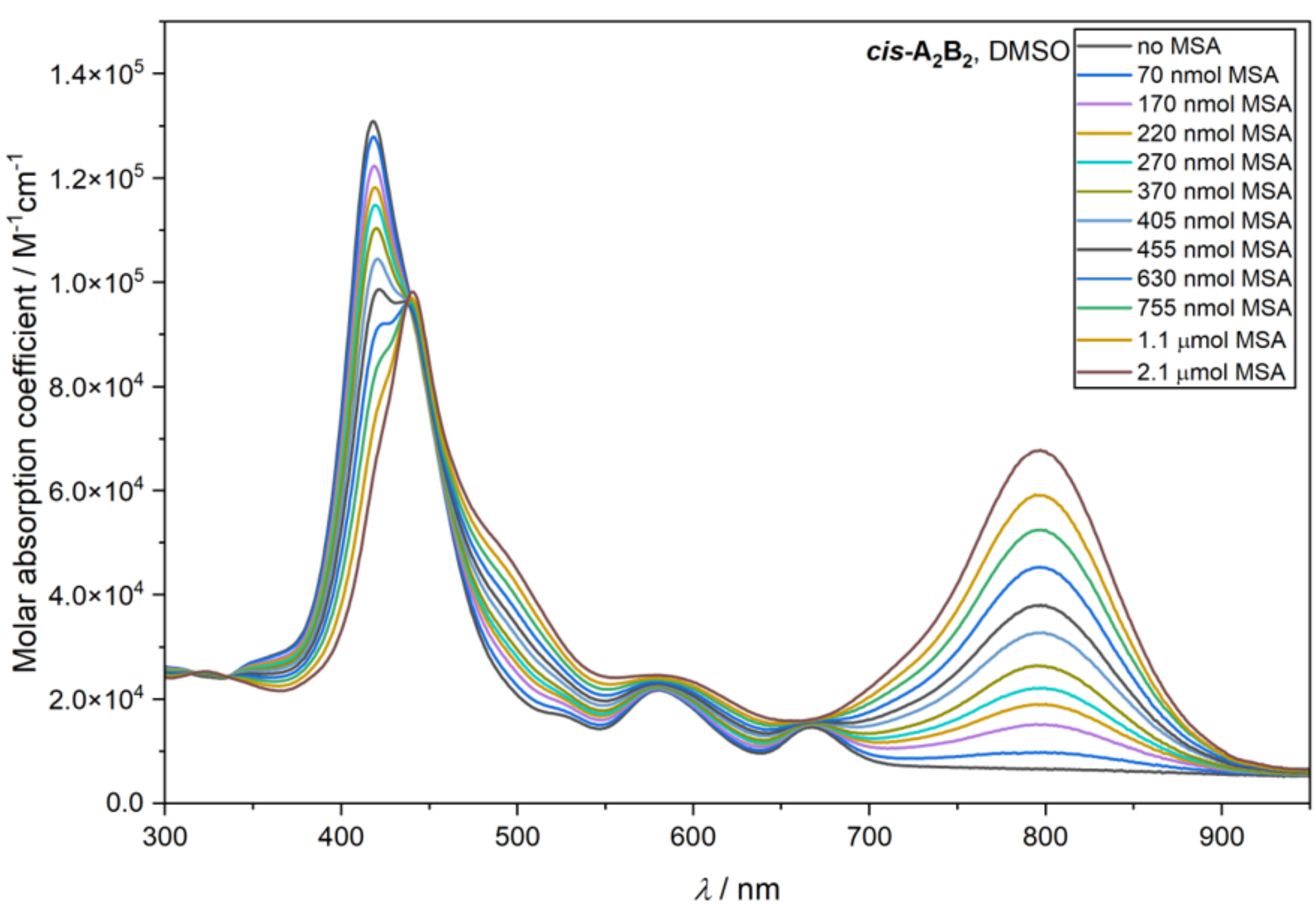

(b)

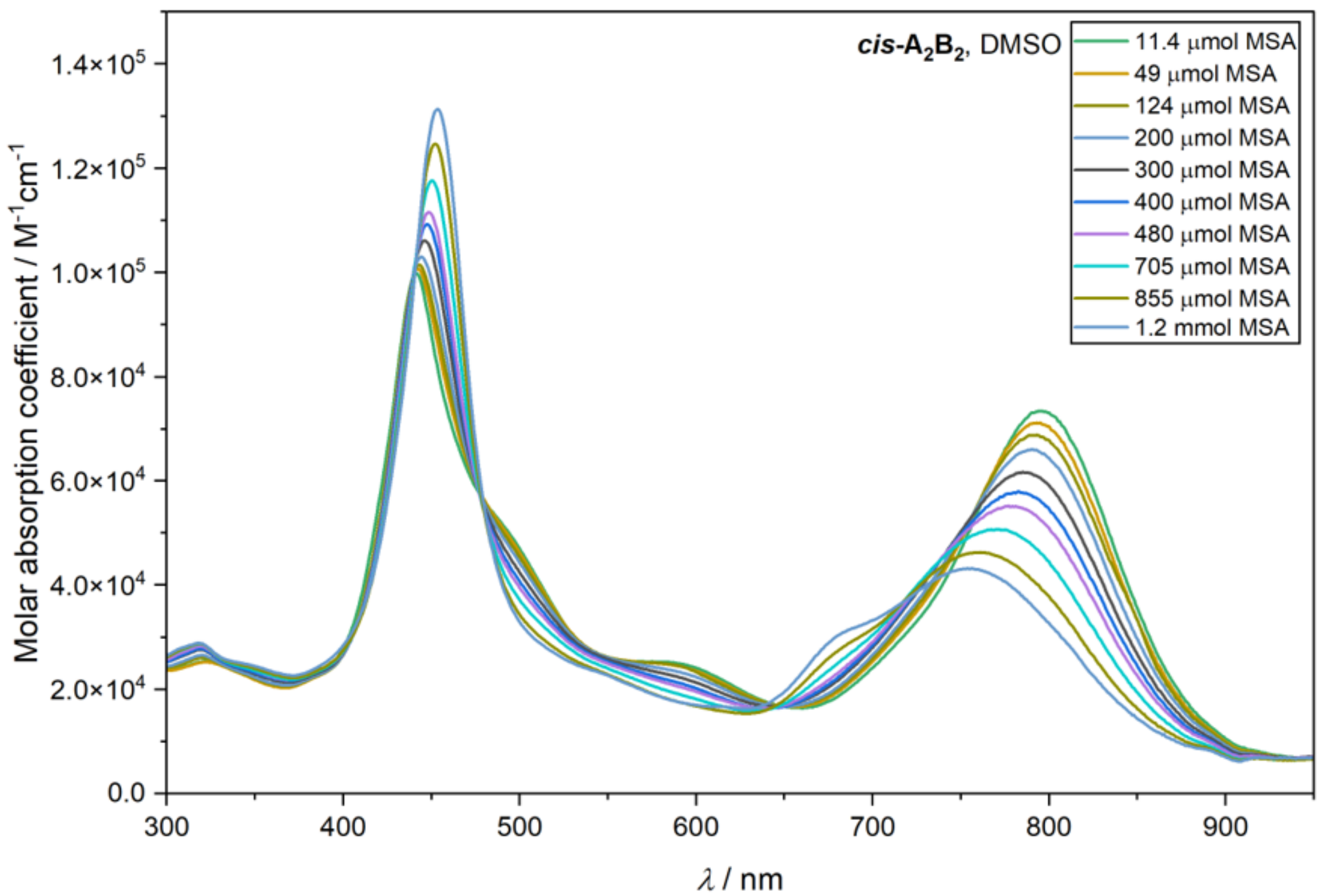

(c)

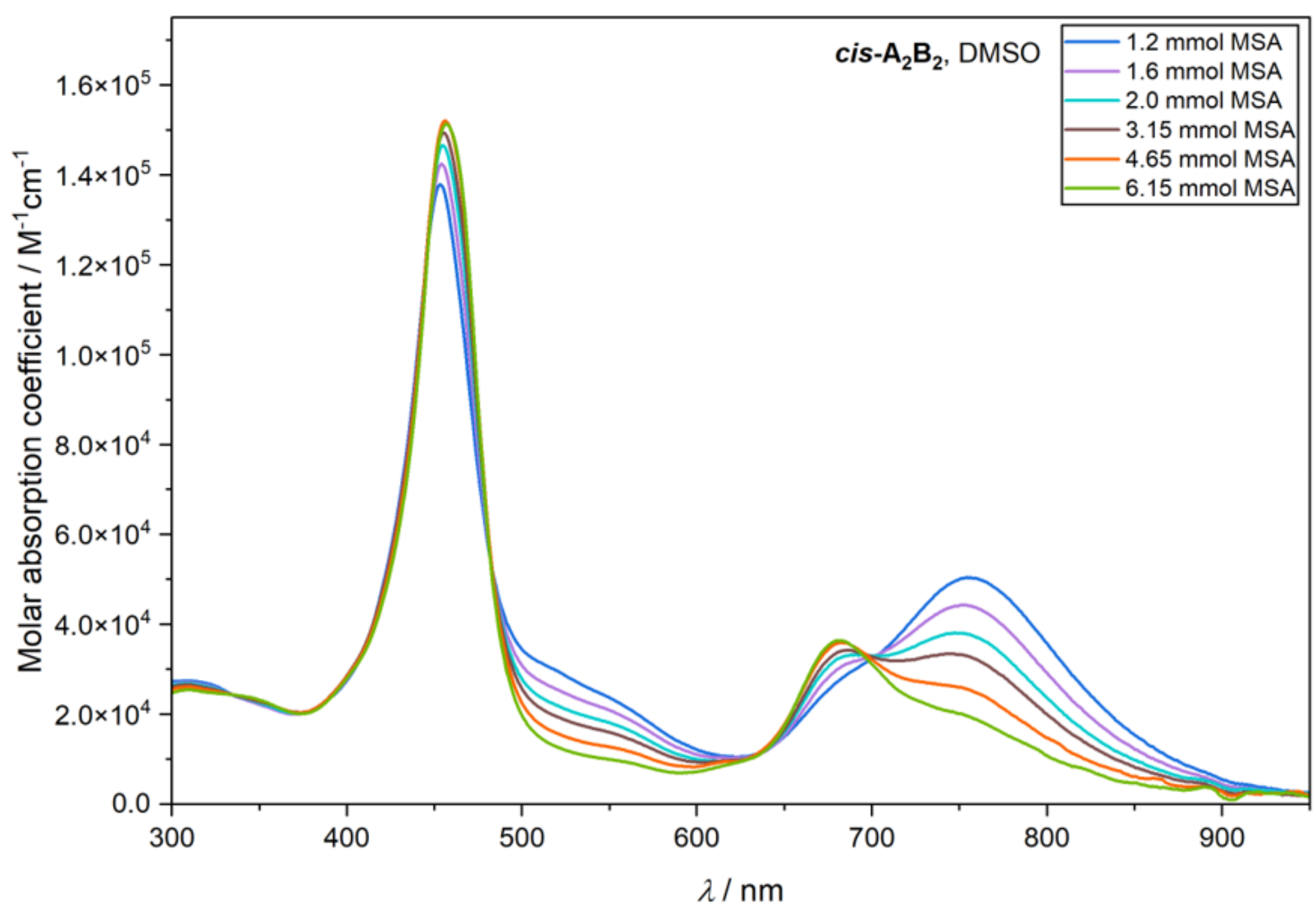

Fig. S32 Absorption spectra of ***cis*-A_2B_2** (6 μM) in DMSO and apparent protonation stages upon addition of MSA: (a) ***cis*-A_2B_2** → ***cis*-$A_2B_2H_2^{2+}$**, 0−2.1 μmol MSA; (b) ***cis*-$A_2B_2H_2^{2+}$** → ***cis*-$A_2B_2H_3^{3+}$**, 11.4 μmol−1.2 mmol MSA; (c) ***cis*-$A_2B_2H_3^{3+}$** → ***cis*-$A_2B_2H_4^{4+}$**, 1.2−6.15 mmol MSA.

(a)

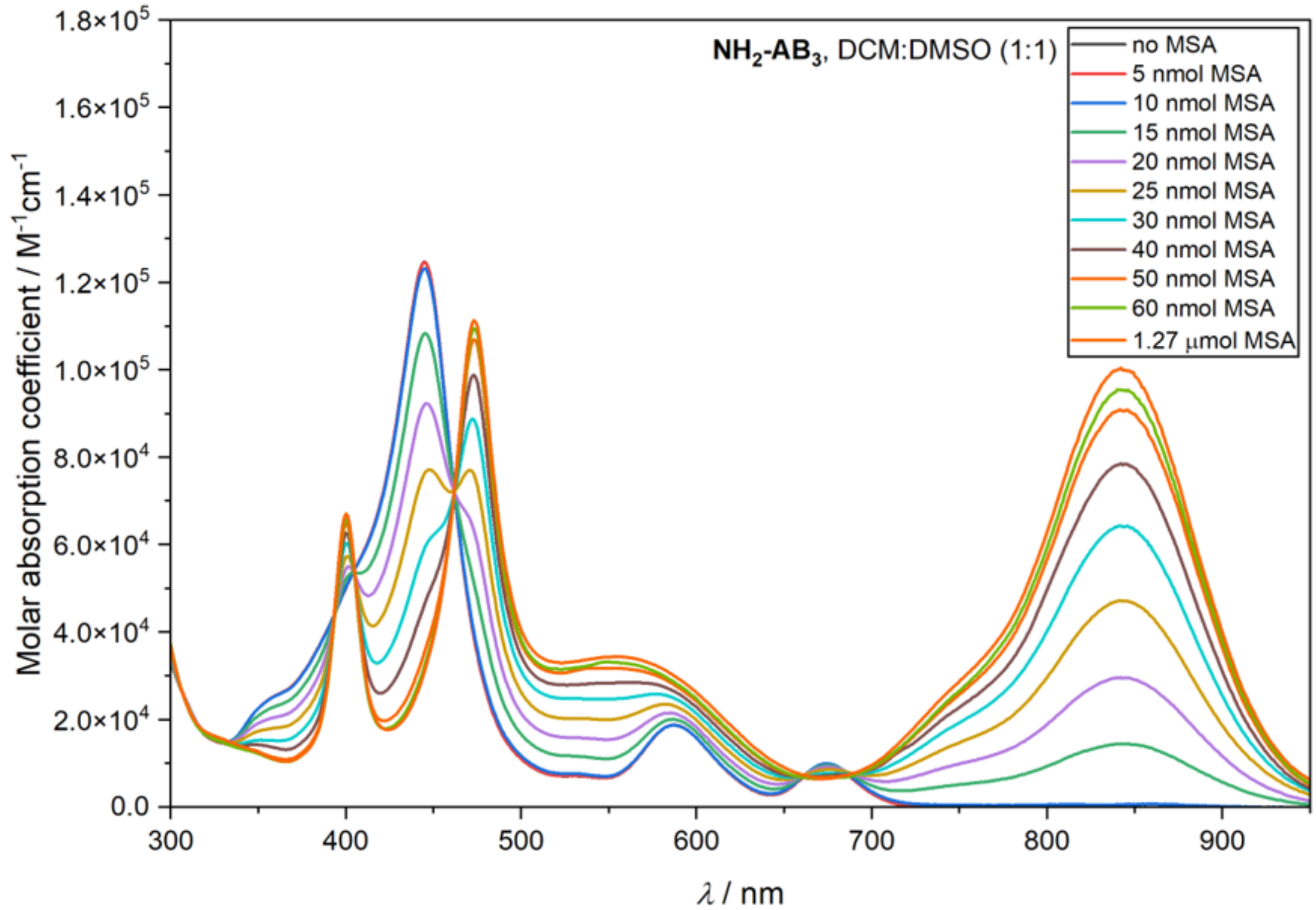

NH2-AB3, DCM:DMSO (1:1)
no MSA
5 nmol MSA
10 nmol MSA
15 nmol MSA
20 nmol MSA
25 nmol MSA
30 nmol MSA
40 nmol MSA
50 nmol MSA
60 nmol MSA
1.27 μmol MSA
Molar absorption coefficient / M-1cm-1
λ / nm

(b)

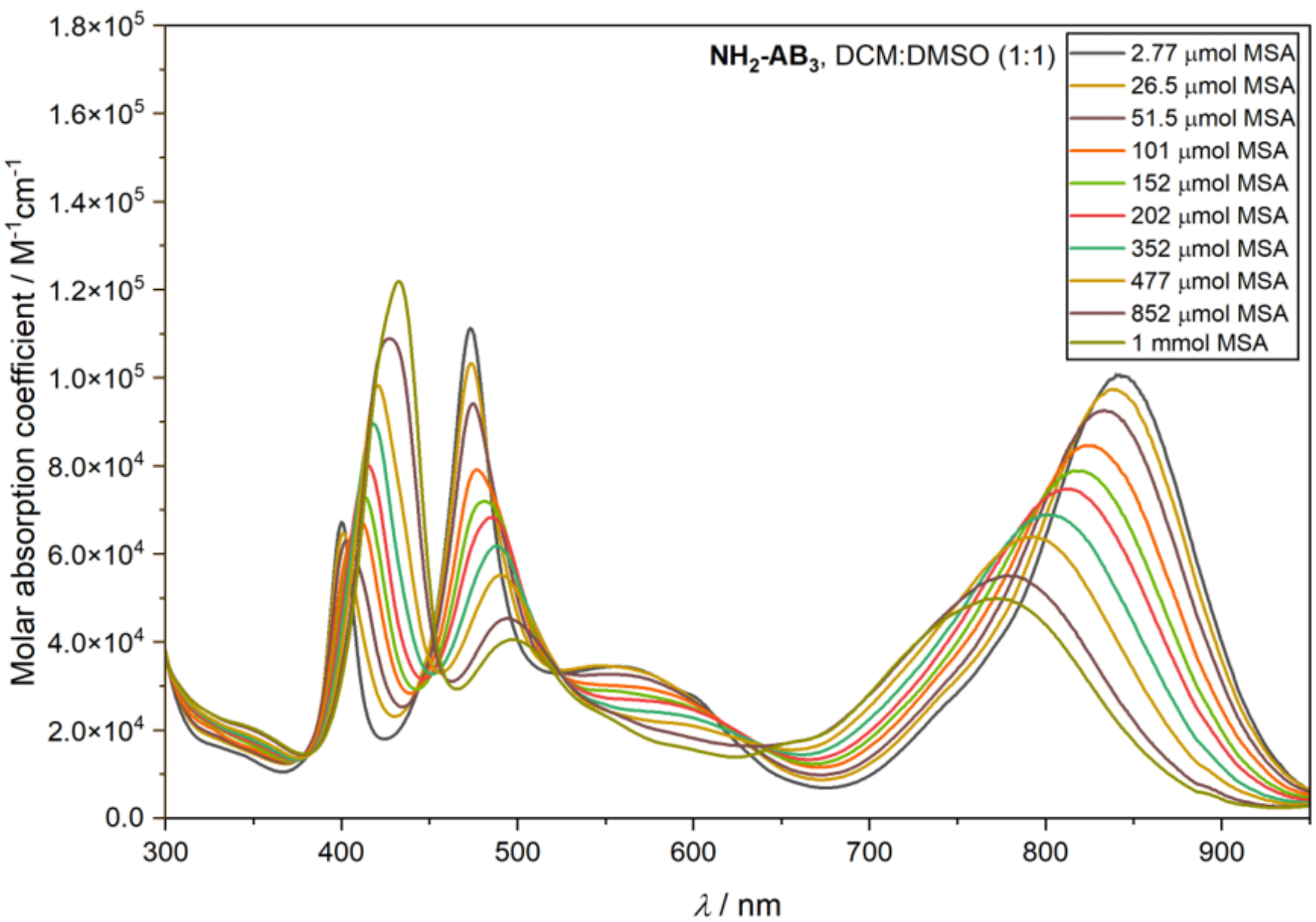

NH2-AB3, DCM:DMSO (1:1)
2.77 μmol MSA
26.5 μmol MSA
51.5 μmol MSA
101 μmol MSA
152 μmol MSA
202 μmol MSA
352 μmol MSA
477 μmol MSA
852 μmol MSA
1 mmol MSA
Molar absorption coefficient / M-1cm-1
λ / nm

(c)

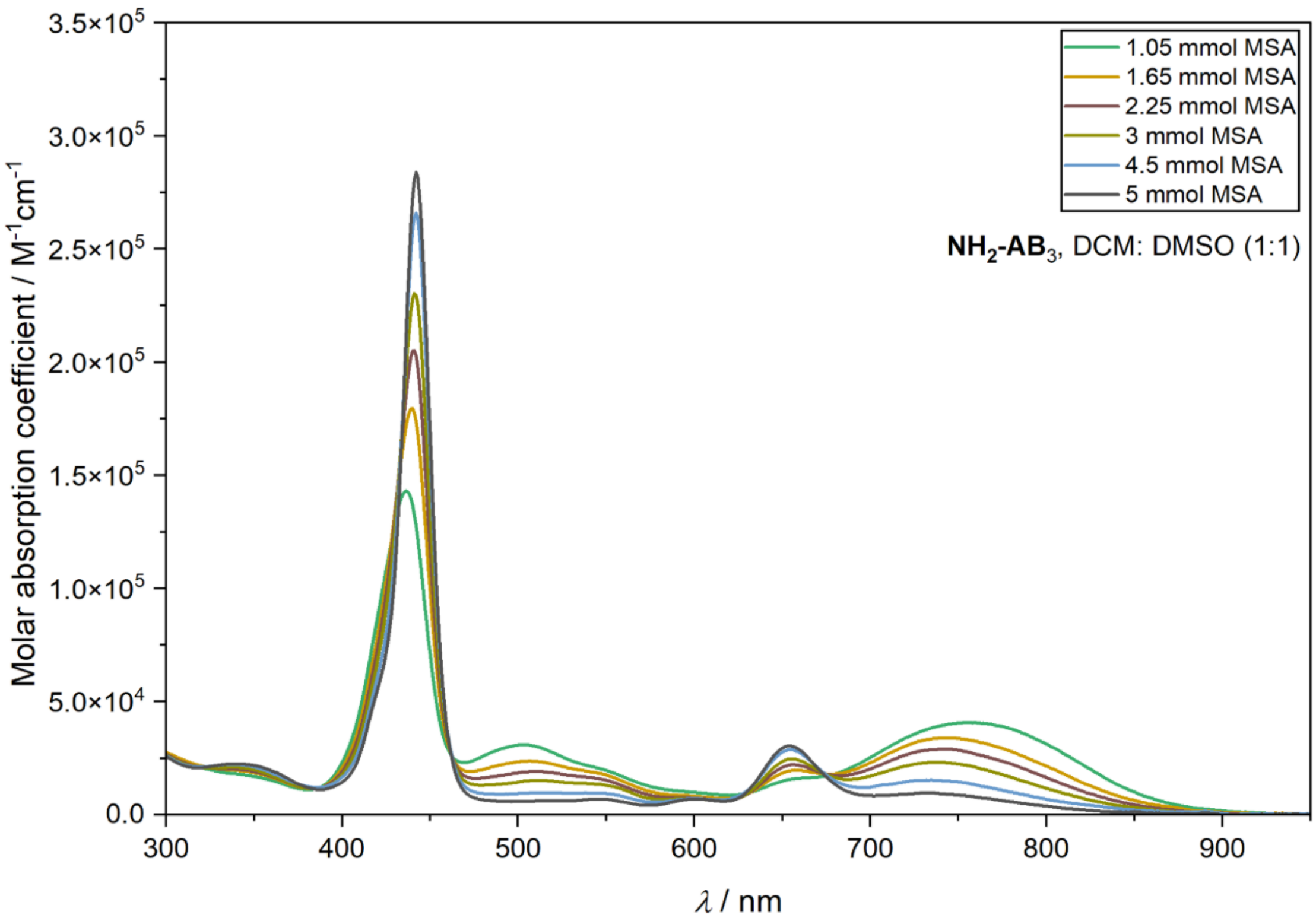

Fig. S33 Absorption spectra of **NH_2-AB_3** (6 µM) in DCM:DMSO (1:1) and apparent protonation stages upon addition of MSA: (a) **NH_2-AB_3** → **NH_2-$AB_3H_2^{2+}$**, 0−1.27 µmol MSA; (b) **NH_2-$AB_3H_2^{2+}$** → **NH_2-$AB_3H_4^{4+}$**, 2.77 µmol−1 mmol MSA; (c) **NH_2-$AB_3H_4^{4+}$** → **NH_2-$AB_3H_6^{6+}$**, 1.05−5 mmol MSA.

6. Fluorescence spectra of acid titrations

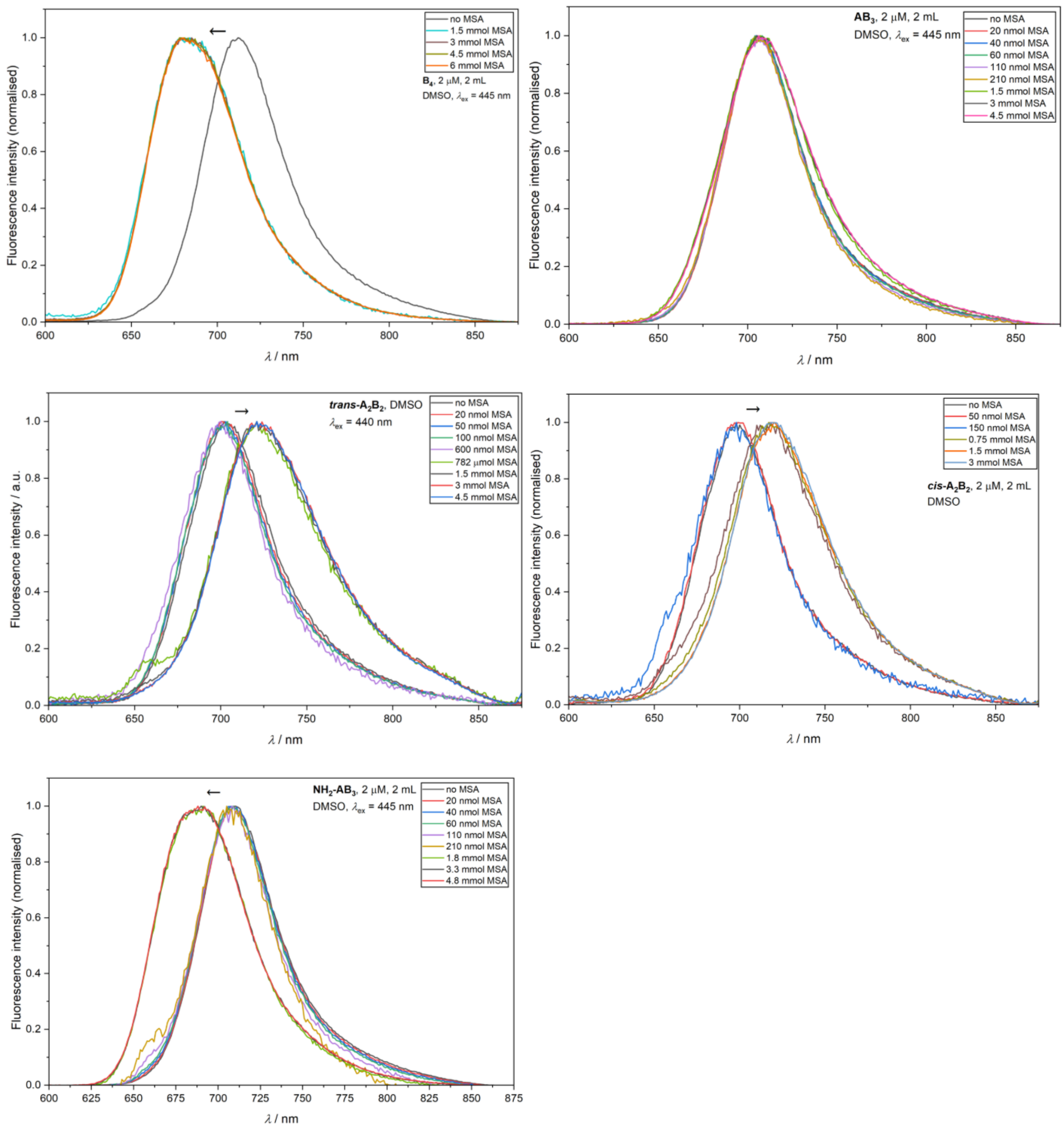

Fig. S34 Normalized fluorescence spectra of porphyrins $\mathbf{B_4}$, $\mathbf{AB_3}$, ***trans*-$\mathbf{A_2B_2}$**, ***cis*-$\mathbf{A_2B_2}$** and $\mathbf{NH_2\text{-}AB_3}$ in 2 µM concentration in DMSO upon addition of concentrated MSA in the amounts from 20 nmol to 6.

7. Light source

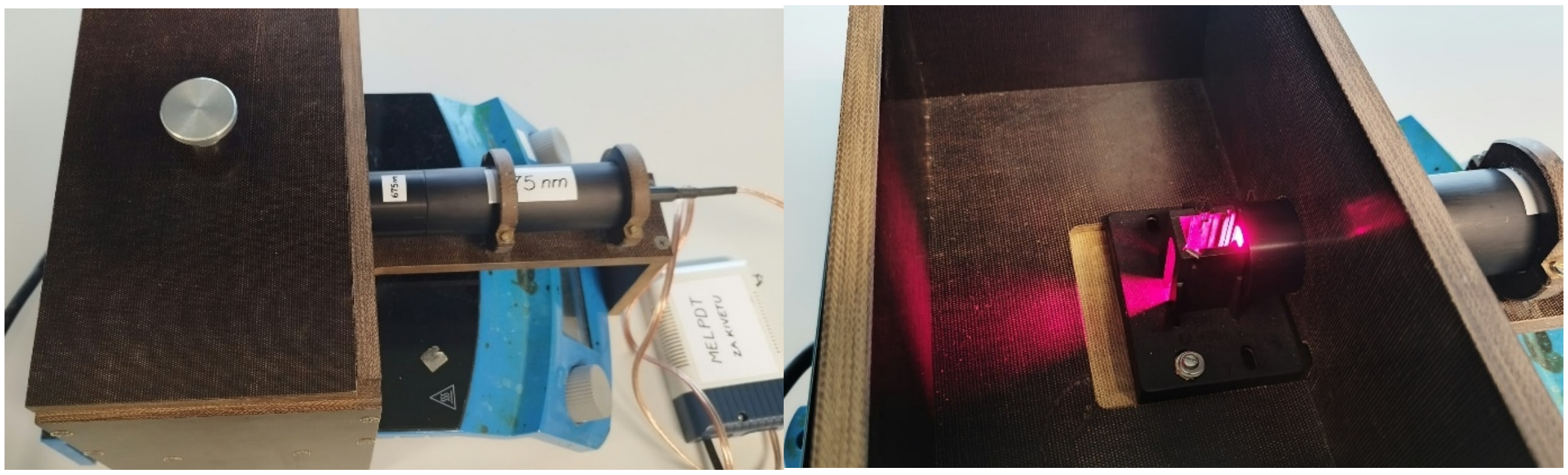

Fig. S35 Set-up with LED-based light source (λ = 675 nm, 41 mW/cm^2) used in experiments measuring ROS production by DPBF photodegradation.

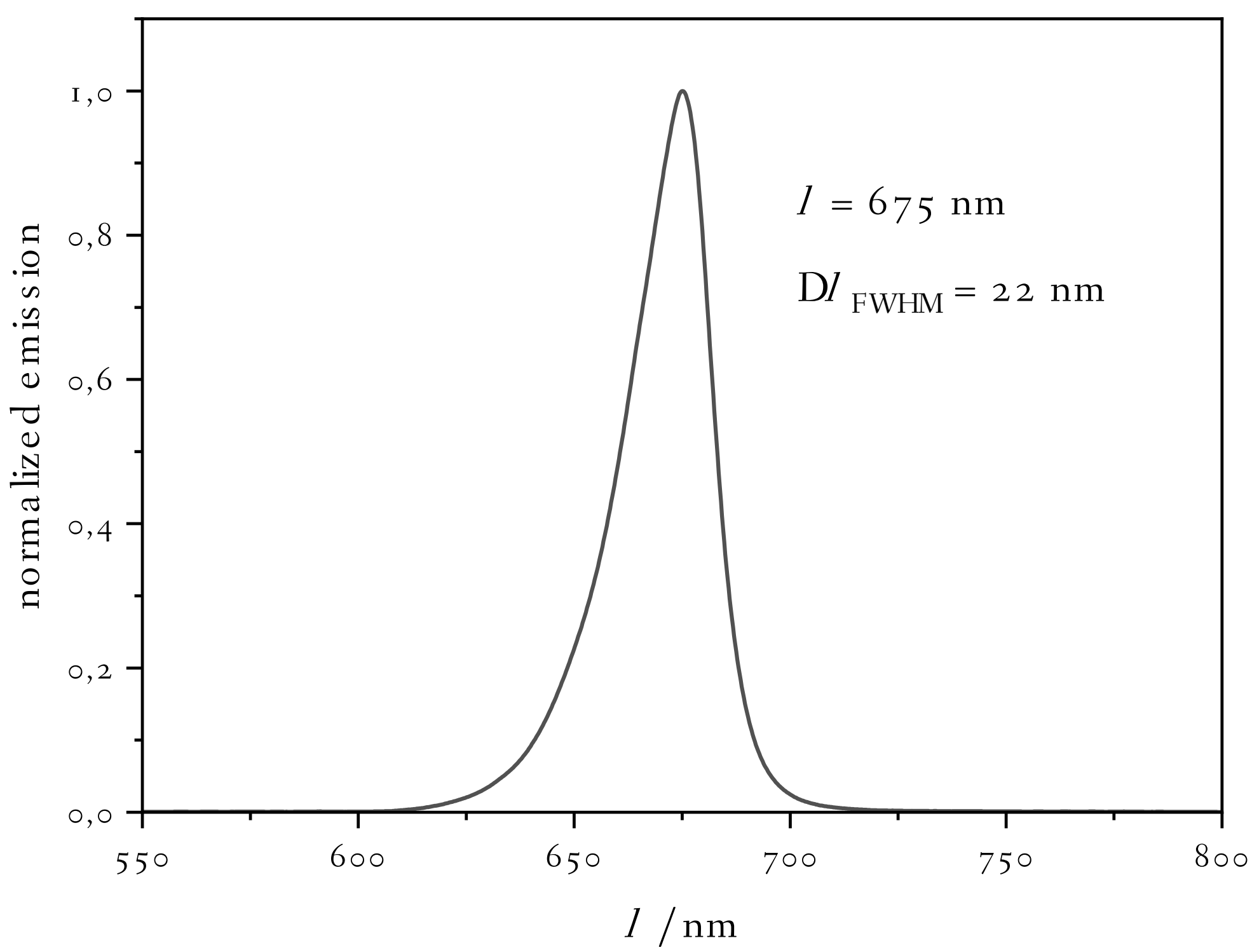

Fig. S36 Normalized emission spectrum of LED-based source of red light at 675 nm used in experiments measuring ROS production by DPBF photodegradation.

8. ROS production

Table S2 Singlet oxygen quantum yield (Φ_Δ) of standard porphyrin-type photosensitizers in the literature:

	**Solvent**	**Method of detection**	$\boldsymbol{\Phi_\Delta}$
**TPP**[1]	C_6H_6	DPBF photodegradation	0.62 [2]
	CH_3Cl		0.55 [2,4]
	toluene	oxygen depletion	0.88 [2,3]
***p*-THPP**[2]	MeOH	TRIL[6]	0.56 [4[
	EtOH	SSIL[7]	0.58 [5]
**TMAP**[3]	PB, pH = 7	TRIL	0.48 [6,2]
	H_2O/PB[9]	RNO[8]	0.69 [7]
**TMPyP4**[4]	H_2O/PB[9]	Oxygen depletion	0.74 [8,9]
**TMPyP3**[5]	H_2O/PB[9]	Oxygen depletion	0.77 [7]

[1]**TPP** = 5,10,15,20-tetraphenylporphyrin,[2]***p*-THPP** = 5,10,15,20-tetra(4-hydroxyphenyl)porphyrin, [3] **TMAP** = 5,10,15,20-tetrakis-(*N*,*N*,*N*-trimethylamoniumphenyl)porphyrin, [4]**TMPyP4** = 5,10,15,20-tetrakis-(*N*-methylpyridinium-3-yl)porphyrin, [5]**TMPyP3** = 5,10,15,20-tetrakis-(*N*-methylpyridinium-3-yl)porphyrin, [6]TRIL = time-resolved luminescence detection of $^1O_2$, [7]SSIL = steady-state luminescence detection of $^1O_2$; [8]RNO = riboflavin trapping of singlet oxygen, [9]PB = phosphate buffer

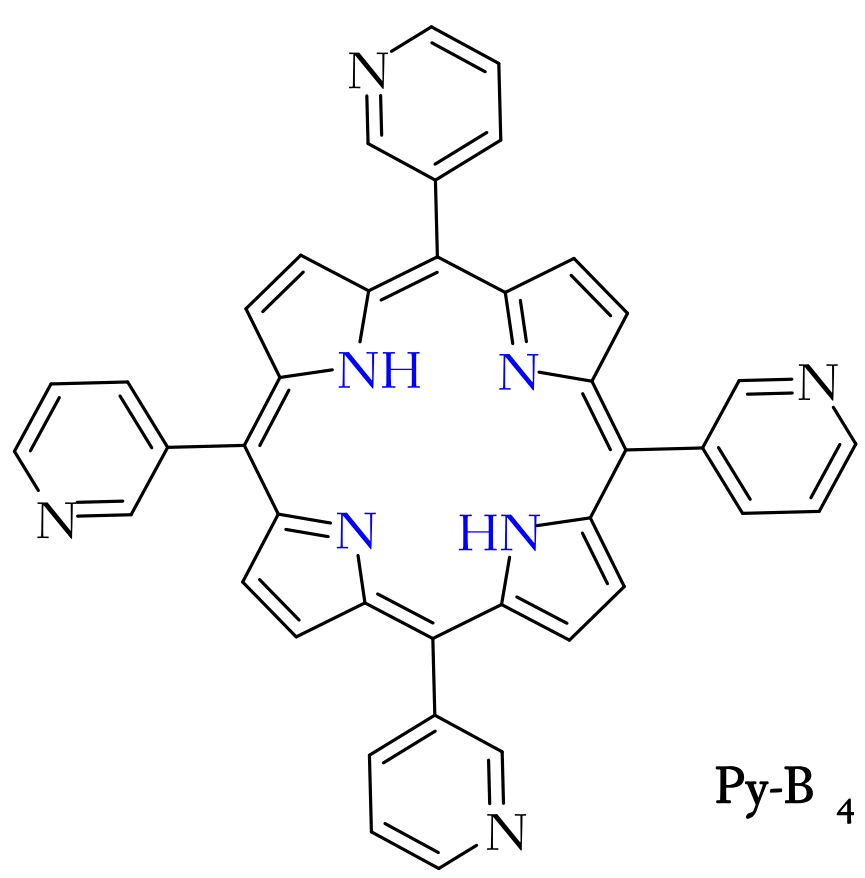

a)

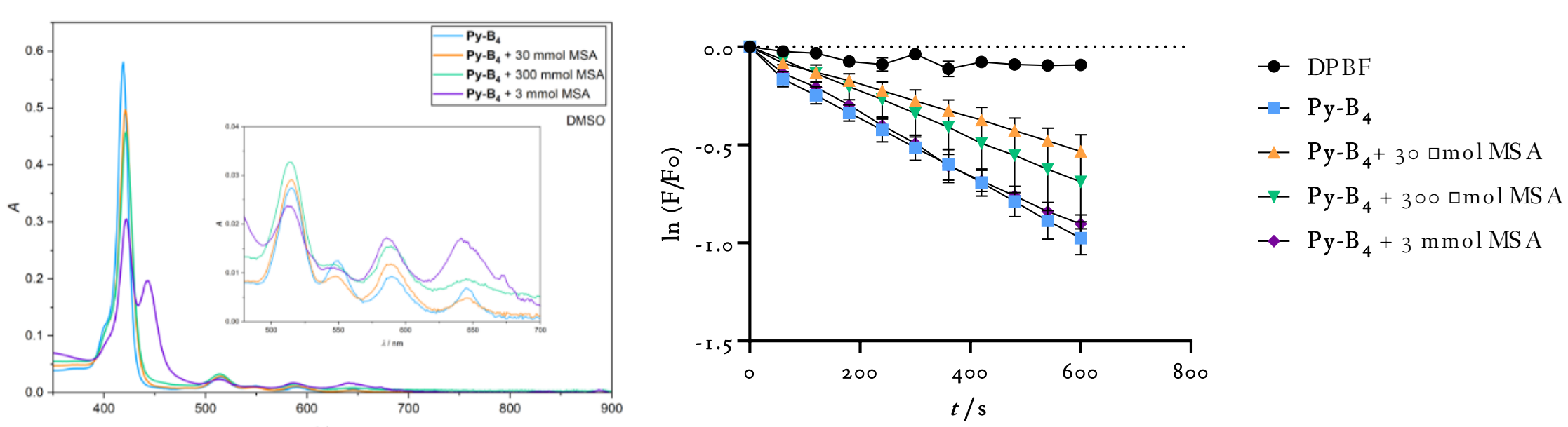

b)

Fig. S37 Photodegradation kinetics of DPBF upon $^1O_2$/ROS production by porphyrin **Py-B$_4$** (2 µM) (a) in DMSO without or with the addition of 30 µmol, 300 µmol and 3 mmol of MSA (b) on the right) at room temperature compared with the absorption spectra under the same conditions (b) on the left).

Photodegradation was measured as the decrease in fluorescence after irradiation for 10 min with red light (λ = 675 nm, 41 mW/cm^2). All results are presented as mean (2), with standard deviation in error bars.

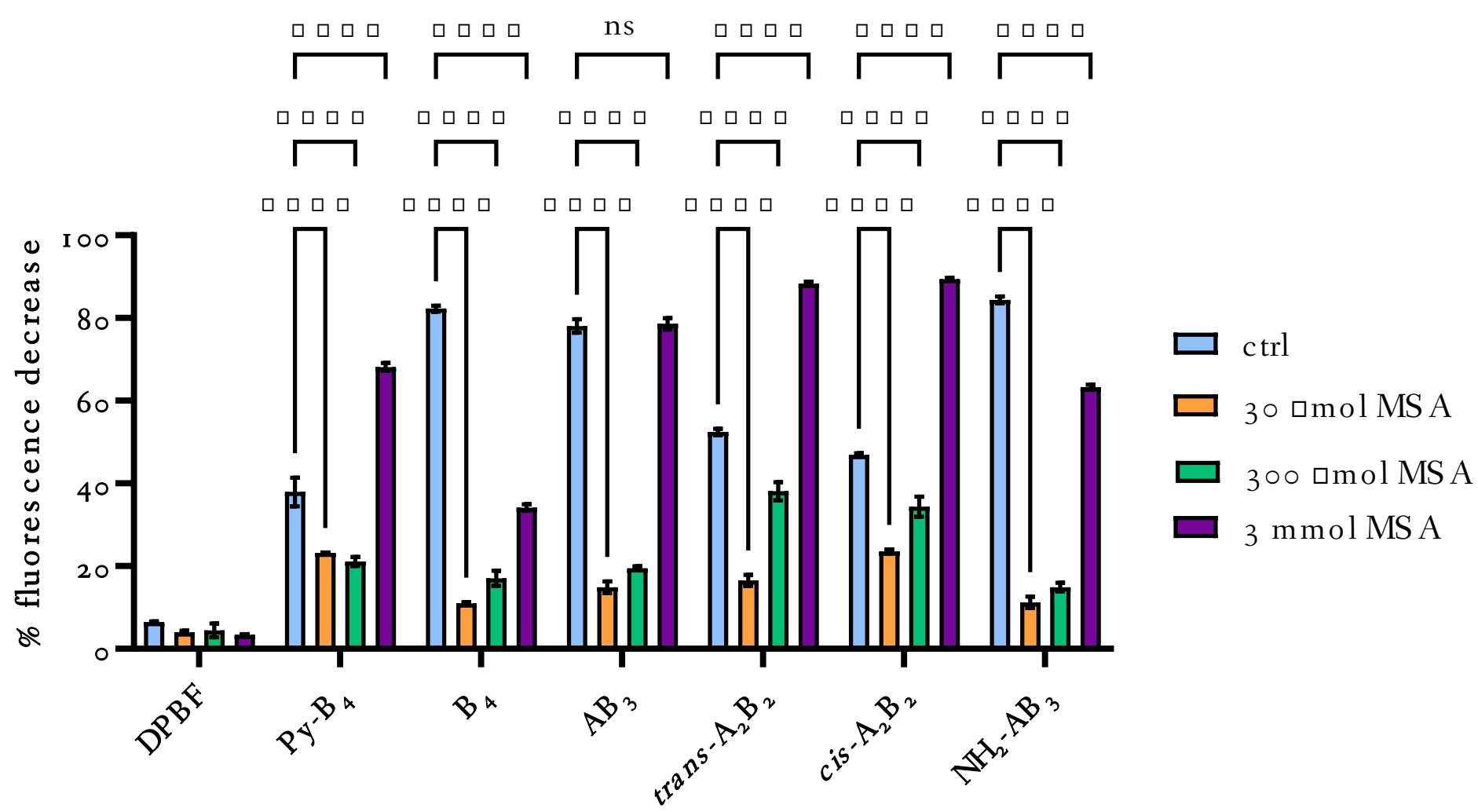

Fig. S38 Time integrated DPBF photodegradation calculated from the AUC of the normalized fluorescence decrease corresponding to $^1O_2$/ROS production of the **Py-B$_4$**, **B$_4$**, **AB$_3$**, ***trans*-A$_2$B$_2$**, ***cis*-A$_2$B$_2$** and **NH$_2$-AB$_3$** in DMSO (ctrl) and after addition of 30 µmol MSA, 300 µmol MSA and 3 mmol MSA. Samples were irradiated for 10 min with red light (λ = 675 nm, 41 mW/cm^2, 24.6 J/cm^2) at room temperature and controls represent the degradation of DPBF under the same conditions without presence of porphyrins. Results are presented as mean (2) with standard deviation in error bars.

Table S3 Number of photons (N_{ph}) absorbed by porphyrins (2 µM in DMSO) and their protonated species formed upon addition of MSA (30 µmol, 30 µmol and 3 mmol) during irradiation in the ROS production assay measured by DBPF photodegradation. Porphyrins were irradiated with red light (675 nm, 41 mW/cm^2, 24.6 J/cm^2) for 10 minutes at room temperature.

	N_{ph} (x 10^{25}) [ph/cm^2]			
	DMSO	+ 30 mmol MSA	+ 300 mmol MSA	+ 3 mmol MSA
**Py-B$_4$**	0.017	0.055	0.167	0.229
**B$_4$**	1.13	1.39	1.51	1.64
**AB$_3$**	1.01	0.78	1.07	1.99
***trans*-A$_2$B$_2$**	0.67	0.97	1.08	2.24
***cis*-A$_2$B$_2$**	0.46	0.66	0.77	2.08
**NH$_2$-AB$_3$**	0.92	0.63	0.87	1.34

References:

[1] C. Wang and C. C. Wamser, *J. Phys. Chem*. **2014**, 118, 3605-3615;

[2] F. Wilkinson, W. P. Helman and A. B. Ross, *J. Phys. Chem. Ref. Data* **1993**, 22, 113–262;

[3] C. Tanielian, L. Golder and C. Wolff, *J. Photochem*. **1984**, 25, 117-125;

[4] R. Bonnet, D. J. McGarvey, A. Harriman, E. J. Land, T. G.Truscott and U.-J. Winfield, *Photochem. Photobiol*. **1988**, 48, 27 1-27;

[5] B. Roeder, D. Naether, T. Lewald, M. Braune. C. Nowak and W. Freyer, *Biophys. Chem.* **1990**, 35, 303-312;

[6] C. R. Lambert, E. Reddi, J. D. Spikes, M. A. J. Rodgers and G. Jori, *Photochem. Photobiol*. **1990**, 44, 59541;

[7] J. B. Verlhac, A. Gaudemer and I. Kraljic, *Now. J. Chim.* **1984**, 8, 401406;

[8] J. W. Snyder, J. D. C. Lambert and P. R. Ogilby, *Photochem. Photobiol.* **2006**, 82, 177-184;

[9] M. Bregnhøj and P. R. Ogilby, *Phys. Chem. Chem. Phys.,* **2026**, 28, 15768.
